# Comparison of Peripheral Blood Regulatory T Cells and Functional Subsets Between Ocular and Generalized Myasthenia Gravis

**DOI:** 10.3389/fmed.2022.851808

**Published:** 2022-06-09

**Authors:** Jie Rao, Siyu Li, Xinyu Wang, Qi Cheng, Yu Ji, Wenwen Fu, Hui Huang, Ling Shi, Xiaorong Wu

**Affiliations:** Department of Ophthalmology, The First Affiliated Hospital of Nanchang University, Nanchang, China

**Keywords:** ocular myasthenia gravis, regulatory T cell, subset, CD45RA, Foxp3

## Abstract

**Purpose:**

This study aims to discuss the function mechanism of regulatory T cells and its subsets in the pathogenic process of myasthenia gravis by contracting the activation levels of those cells in peripheral blood among healthy people, patients with ocular myasthenia gravis (oMG) and patients with generalized myasthenia gravis (gMG).

**Method:**

Healthy people, newly diagnosed oMG patients, and gMG patients were enrolled in this study. The percentage of the CD3^+^CD4^+^CD25^+^ Treg cells, CD3^+^CD4^+^CD25^+^Foxp3^+^ Treg cells, CD3^+^CD4^+^CD25^+^Foxp3^hi^ CD45RA^–^aTreg cells, CD3^+^CD4^+^CD25^+^Foxp3^lo^CD45RA^–^n-sTreg cells, and CD3^+^CD4^+^CD25^+^ Foxp3^lo^CD45RA^+^rTreg cells in the peripheral blood were examined by flow cytometry. And then analyzed the differences of Treg cells and its subsets among the study members.

**Results:**

The percentage of the CD4^+^CD25^+^Treg cells in the peripheral blood of oMG patients and gMG patients were both lower than that of healthy people (*p* < 0.05), the percentage of patients with oMG had no distinct difference with that of patients with gMG (*p* = 0.475), however. Also, the percentage of CD3^+^CD4^+^CD25^+^Foxp3^+^Treg cells in the oMG and gMG patients’ group were both lower than that of healthy group. And the percentage of CD25^+^Foxp3^+^Treg cells in the peripheral blood of patients with oMG and healthy people were both higher than that of patients with gMG (*p* < 0.05). The percentage of rTreg in the CD3^+^CD4^+^CD25^+^Treg of the peripheral blood for both gMG and oMG patients’ group were lower than healthy group (*p* < 0.05), but there was no statistical significance between the oMG and gMG patients’ group (*p* = 0.232). The percentage of the aTreg cells in the CD3^+^CD4^+^CD25^+^Treg cells of the peripheral blood for the oMG patients was higher than that of gMG patients (*p* < 0.05), but both of them were lower than healthy group (*p* < 0.05). The percentage of n-sTreg cells in the peripheral blood descended among the gMG patients’ group, oMG patients’ group, and healthy group (*p* < 0.05).

**Conclusion:**

The changes in the number and function of Treg cells and its subsets can cause the impairment of negative immune regulation, which may mediate the triggering of oMG and its progression to gMG.

## Introduction

Myasthenia gravis (MG) is an autoimmune disorder presenting with neuromuscular transmission impairment and fatigable muscle weakness, which antibodies cause morphological and functional alterations of the neuromuscular junction membrane (1). There are some studies reporting that the worldwide prevalence of MG is 40–180 per million people, and the annual incidence is 4–12 per million people. Meanwhile, prevalence and incidence tend to be higher than older ones because of widespread autoantibody testing (2). Ocular myasthenia gravis (oMG), whose weakness is restricted to the ocular muscle, including extra ocular, bulbur, limb, and axial muscles, has the manifestation of ptosis and diplopia. Besides with eye muscle weakness, generalized myasthenia gravis (gMG) can involve with pharynx, skeletal, and even respiratory muscle weakness. As the different stages of MG, oMG patients, with purely ocular weakness, are at risk of developing generalized myasthenia gravis, especially early in the disease. 90% of those who have had the ocular form for more than 2 years will progress to gMG and remain in this subgroup (3).

The pathogenesis of MG is mediated by multiple pathogenic antibodies, such as acetylcholine receptor antibody (AChR-Ab), muscle-specific kinase antibody (Musk-Ab), low density lipoprotein receptor-related protein 4 antibody (LRP4-Ab), different cytokines, and complement activation pathways (4). Under the condition of imbalanced immune homeostasis, stimulated by cytokines secreted by CD4^+^ T helper cell (Th), B cells secrete pathogenic antibodies with high affinity through proliferation and differential differentiation, and activate complement cascade reaction to form membrane attack complex and destroy acetylcholine receptor on NMJ endplate membrane (5). The dysregulated immune response prevents the effective transmission of neuroexcitatory transmitters, leading to clinical symptoms such as ptosis, muscle fatigue, and weakness, and even dyspnea in patients with MG (6).

CD4^+^ CD25^+^ Foxp3^+^ regulatory T cells (Tregs), are produced in the thymus or generates from conventional CD4^+^ T cells in peripheral sites, suppress the activation and proliferation of autoreactive cells to control the immune response to antigens and play a fundamental role in the maintenance of immune homeostasis, which have been found numerically and functionally deficient in development and progression of some autoimmune diseases (7). Besides B-cells, which rely on the action of CD4^+^ T helper (Th) cells, could produce pathogenic autoantibodies to destruct the neuromuscular endplate, Tregs were also necessary in pathogenesis of MG (8). Although some studies differ concerning the existence of a deficit in Treg cell numbers, Treg cells have been shown to be functional defects and numerical changes in patients with MG (9).

Human CD4^+^ CD25^+^ Foxp3^+^ T cells can be divided into three phenotypically and functionally distinct subpopulations: CD45RA^+^ Foxp3^lo^ resting Treg cells (rTreg cells), CD45RA^–^Foxp3^hi^ activated Treg cells (aTreg cells), and CD45RA^–^Foxp3^lo^ non-suppressive T cells (n-s Treg cells) (10, 11). Changes in the number of Treg subsets can be observed in peripheral blood of patients with autoimmune diseases, such as systemic lupus erythematosus (12), idiopathic thrombocytopenic purpura (13), and Type 1 diabetes (14). Therefore, the imbalance of the proportion of Treg subsets may be an important factor in the pathogenesis of many immune-related diseases. In addition, studies have found that the frequency of rTreg subsets in peripheral blood of MG patients is lower than that of the normal population, and the proportion of Treg subsets changes after immunosuppression or thymotomy (15). However, there are few studies on Treg cell subsets in oMG at present, and their specific mechanism of action has not been clarified in detail. This project intends to explore the possible roles and differences of Treg cells and their functional subsets in the occurrence and progression of oMG in gMG by detecting the expression levels of Treg cells and subsets in peripheral blood of healthy patients, oMG and gMG patients.

## Materials and Methods

### Patients and Healthy Controls

From February 2020 to 2021, this study enrolled patients with newly diagnosed oMG and gMG according to MGFA (myastheniagravis foundation of America) clinical classification in the Ophthalmology and Neurology Outpatient and Inpatient Departments of the First Affiliated Hospital of Nanchang University. Meanwhile, enrolled patients were meeting the following criteria: (1) clinical diagnosis of MG (fluctuating muscle weakness and fatigue, positive neostigmine test, and/or neurophysiological abnormalities in low frequency repetitive nerve electrical stimulation test and single fiber electromyography); (2) AChR-Ab, Musk-Ab, LPR4-Ab, or Titin-antibody positive.

Exclusion criteria were as follows: (1) received glucocorticoid, immunosuppressive drugs, gamma globulin, and plasmapheresis within 3 months before to the study; (2) a history of thymoma and received thymic medical or surgical treatment; (3) coexisting autoimmune or inflammatory diseases; (4) hereditary or metabolic myasthenia; (5) congenital or traumatic ptosis and muscle weakness.

Healthy control (HC) volunteers were recruited at the Health Examination Center of the First Affiliated Hospital of Nanchang University; all HCs had no history of autoimmune diseases, treatment with immunosuppressants in the past 2 years, and exhibited normal neurological and systemic medical findings. All patients and HCs were provided written informed consent before enrollment. The study was approved by the Ethics Committee of the First Affiliated Hospital of Nanchang University.

### Flow Cytometry Analysis

Peripheral blood mononuclear cells (PBMCs) (1 × 10^6^) were stained with CD4-FITC; CD45RA-PE-Cyanine7, CD3-PE-CF594, and CD25-PE-Cyanine5. Cells samples were incubated for 15 min at room temperature, followed by washing twice with physiological saline. Intracellular staining was performed by using Foxp3/Transcription Factor Staining Buffer Set and Foxp3-PE. All antibodies and reagents were purchased from Biolegend, BD Biosciences, Thermo Fisher Scientific and used according to the instructions. Samples were acquired on a FC-500-MCL/MPL flow cytometer (Beckman Coulter Inc., Brea, CA, United States) and analyzed using CXP Analysis software.

### Statistical Methods

All analyses were performed with SPSS 19.0 statistical software (IBM Corp., Armonk, NY, United States) and Graph Pad Prism 6.0 (GraphPad, La Jolla, CA, United States). Measurement data were expressed as Mean ± SD. Shapiro–Wilk *W* test was used to assess normality. When multiple groups were compared, 1 Way ANOVA and Kruskal–Wallis *H* test were used for data fulfilled normal distribution and for those did not, respectively. Tukey test and Games–Howell test were used for data fulfilled homogeneity of variance and for those did not, respectively. Description of count data adoption rate (%), χ2 test was used for comparison among groups, Pearson analysis was used to evaluate the correlation between groups. The two-sided *p*-values < 0.05 was considered statistically significant for all tests (*p < 0.05, ^**^p < 0.001).

## Results

The modified Osserman score was used to divide 39 patients with MG into 2 groups of 3. oMG patients group (only restricted to the ocular muscle, 9 women and 9 men, mean ages 46.11 ± 13.51 years) and gMG patients group (not restricted to the ocular muscle, 11 women and 10 men, mean ages 43.29 ± 12.22 years) in this study. About nineteen patients with HC were enrolled (10 women and 9 men, mean ages 43.74 ± 11.35 years). The age and sex among the three groups were comparable (*p* > 0.05). Detail characteristics were listed in Table 1.

**TABLE 1:** Clinical characteristics of patients.

	HC group	oMG group	gMG group	*p*
Number	19	18	21	N/A
Sex (M/F)	9/10	9/9	10/11	0.977
Age (Years)	43.74 ± 11.352	46.11 ± 13.508	43.29 ± 12.215	0.754

### Changes in the Proportions of Treg and Function Cells

**FIGURE 1:**
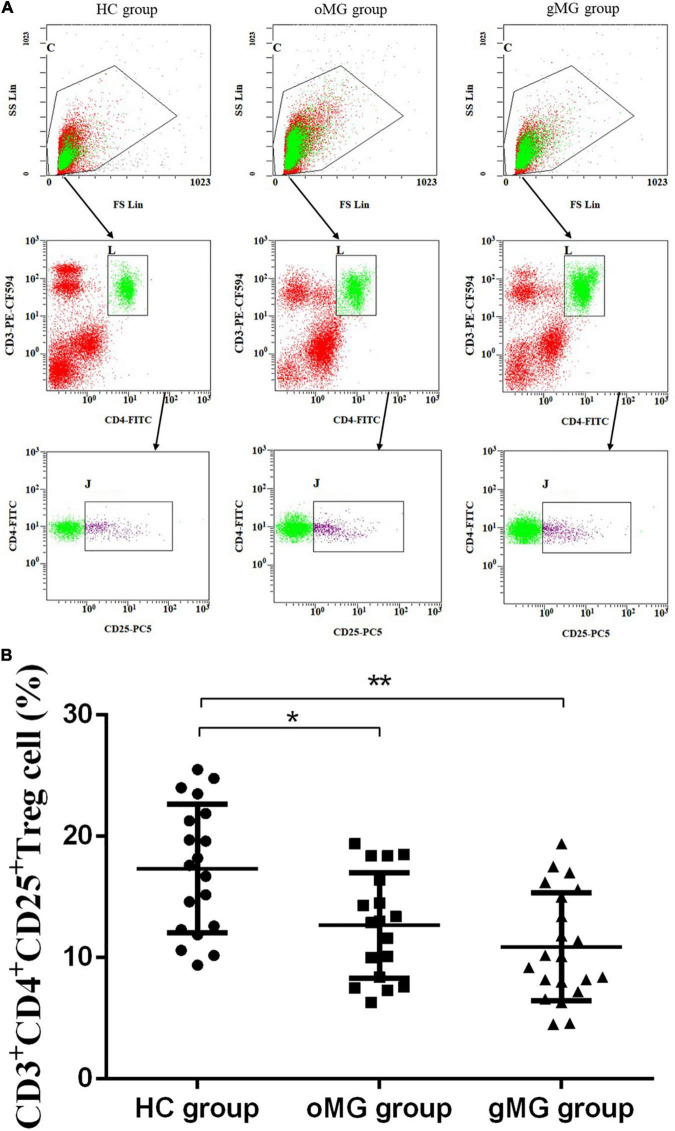
Different expression of CD3^+^CD4^+^CD25^+^ Treg cells in peripheral blood of MG patients (oMG: *n* = 18, gMG: *n* = 21) and HCs (*n* = 19). **(A)** Representative flow cytometry plots of CD3^+^CD4^+^CD25^+^ Treg cells in MG patients and HCs. **(B)** Comparison of the frequency of CD3^+^CD4^+^CD25^+^ Treg cells between MG patients and HCs. The results are expressed as the Mean ± SD of three independent experiments (*p < 0.05, **p < 0.001).

Based on the above CD3^+^CD4^+^ Tlymphocytes gates, we analyzed CD3^+^CD4^+^CD25^+^ Treg cells. As shown in Figure 1, the percentages of CD4^+^CD25^+^Treg cells in the HC group, the oMG group and the gMG group were 17.35 ± 5.28, 12.67 ± 4.34, and 10.90 ± 4.44%. The percentage of Treg cells in peripheral blood CD3^+^CD4^+^T lymphocytes of oMG patients and gMG patients were lower than that of healthy controls (*p* < 0.05), but there was no significant difference between patients with oMG and patients with gMG (*p* = 0.475).

**FIGURE 2:**
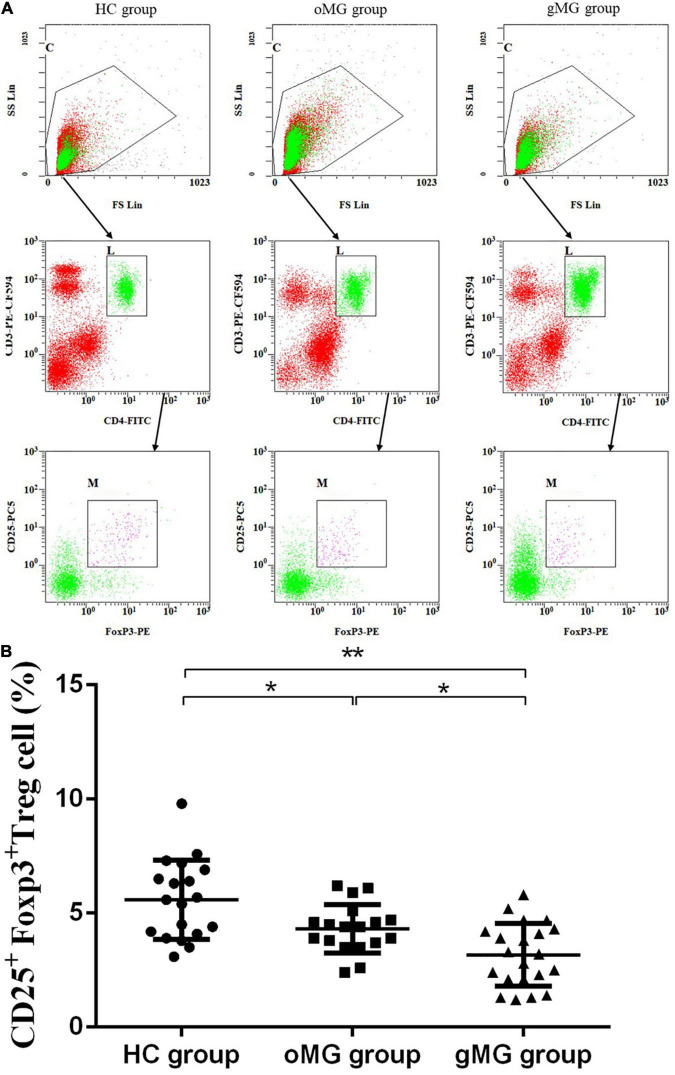
Different expression of CD3^+^CD4^+^CD25^+^Foxp3^+^ Treg cells in peripheral blood of MG patients (oMG: *n* = 18, gMG: *n* = 21) and HCs (*n* = 19). **(A)** Representative flow cytometry plots of CD3^+^CD4^+^CD25^+^Foxp3^+^ Treg cells in MG patients and HCs. **(B)** Comparison of the frequency of CD3^+^CD4^+^CD25^+^Foxp3^+^ Treg cells between MG patients and HCs. The results are expressed as the Mean ± SD of three independent experiments (*p < 0.05, **p < 0.001).

Meanwhile, the percentage of CD25^+^Foxp3^+^ Treg cells in peripheral blood of HCs, oMG patients, and gMG patients were 5.589 ± 1.74, 4.32 ± 1.06, and 3.17 ± 1.38%, respectively. The proportions of CD25^+^ Foxp3^+^Treg cells in the gMG and oMG group were lower than that in the HC group (*p* < 0.05). Both patients with oMG and HCs were higher than gMG patients (*p* < 0.05) (Figure 2). This result suggested that the proportions of Treg and its function cells were decreased with the progression of MG.

### Different Changes in Treg Functional Subsets

**FIGURE 3:**
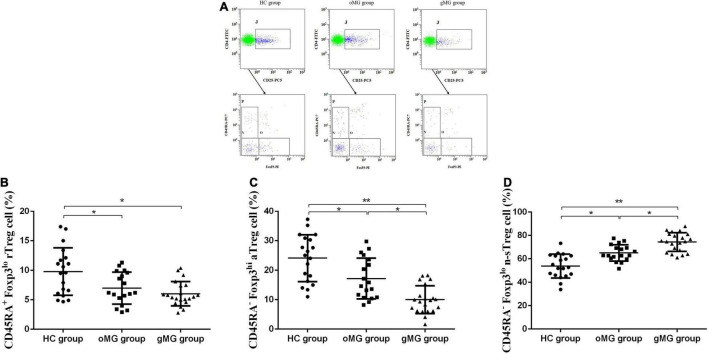
Different expression of Treg functional subsets in peripheral blood of patients with MG (oMG: *n* = 18, gMG: *n* = 21) and HCs (*n* = 19). **(A)** Representative flow cytometry plots of Treg functional subsets in HCs and MG patients. **(B)** Comparison of the frequency of CD3^+^CD4^+^CD25^+^Foxp3^lo^CD45RA^+^rTreg cells between HCs and MG patients. **(C)** Comparison of the frequency of CD3^+^CD4^+^CD25^+^Foxp3^hi^CD45RA^–^aTreg cells between MG patients and HCs. **(D)** Representative flow cytometry plots of CD3^+^CD4^+^CD25^+^Foxp3^lo^CD45RA^–^ n-sTreg cells in HCs and MG patients. The results are expressed as the Mean ± SD of three independent experiments (*p < 0.05, **p < 0.001).

We compared the percentage of Treg subsets in CD4^+^CD25^+^T lymphocytes of PBMCs in peripheral blood of the oMG group, gMG group, and HC group (Figure 3A). The results showed that the proportion of CD45RA^+^Foxp3^lo^rTreg of the HC group, the oMG group, and the gMG group were 9.80 ± 4.01, 6.99 ± 2.72, and 6.02 ± 2.06%. For patients with oMG and gMG, rTreg cells were decreased as compared to HC group (*p* < 0.05), but there was no statistically significant difference between oMG patients and gMG patients (*p* = 0.232) (Figure 3B).

For CD45RA^–^Foxp3^hi^aTreg cells, the percentage of the HC group, the oMG group, and the gMG group were 24.07 ± 7.96, 17.18 ± 6.91, and 10.00 ± 4.73%. The proportion of aTreg cells in patients with oMG was higher than that in gMG patients (*p* < 0.05), and both groups were lower than that in the HC group (*p* < 0.05) (Figure 3C).

On the other hand, the proportion of CD45RA^–^Foxp3^lo^ n-sTreg cells in the HC group, the oMG group and the gMG group were 53.89 ± 10.15, 65.02 ± 7.01, and 74.34 ± 8.04%. The proportion of n-sTreg cells in PBMCs of patients with gMG was higher than that of HC and oMG patients (*p* < 0.05), and the proportion in oMG patients’ group was higher than the HC group (*p* < 0.05) (Figure 3D).

## Discussion

Myasthenia gravis is an acquired autoimmune disease with NMJ involvement mediated by pathogenic autoimmune antibodies. The occurrence and progression of MG involves a complex immune regulatory network, including multiple immune cell interactions, cytokines, and different complement activation pathways, and CD4^+^ T lymphocytes play an important role in the synthesis of MG pathogenic antibodies (16). Hu et al. found that the imbalance of immune response mechanism, which caused by the difference in activation level of T lymphocyte subsets, plays a key role in triggering oMG and inducing gMG production (17).

Treg cells are a type of T cell subgroup with immunosuppressive regulation function, which regulate the immune response by inhibiting the functional expression of effector CD4^+^T cells. Once Treg cells are declined in number or dysfunctional, the imbalance of homeostasis of the immune system and the failure of peripheral immune tolerance can be caused. This is a key factor in the occurrence and development of autoimmune diseases such as multiple sclerosis (18), systemic lupus erythematosus (19), and type 1 diabetes (14). Studies have found that Treg cells are closely related to the triggering and progression of MG, but there are differences on the number of Treg cells in peripheral blood of patients with MG. For example, it has been reported that the number of CD4^+^CD25^+^Treg cells in peripheral blood of HCs is significantly higher than patients with MG without any treatment (20). In addition, it was also reported that the proportion of Treg cells in peripheral blood of MG patients was not significantly altered (21, 22). Our study found that CD4^+^CD25^+^Treg cells in PBMCs of patients with oMG and patients with gMG were lower than those of HCs and the difference between oMG patients and gMG patients was not statistically significant, suggesting that the onset of MG was related to the reduction of Treg cells. At the same time, we noticed that more studies have confirmed that the deficiency of immunomodulatory function of Treg cells is essential in the pathogenesis of MG (23, 24). As a key transcription factor of CD4^+^CD25^+^Treg cells, mutation or deletion of Foxp3 gene can lead to congenital deletion of Treg cell differentiation and immunomodulation function.

Foxp3 can join in the synthesis and secretion of Tregs’ pro-inflammatory cytokines such as IL-2, IL-4, IL-17A, and IFN-γ (25). It can also mediate the inhibitory immunomodulatory function of Treg cells to maintain peripheral immune tolerance, by binding with transcription factors like nuclear factor -κB (NF-κB), activator protein 1 (AP1), retinoic acid orphan receptor-α (ROR-α) (26). Our study found that the proportion of CD3^+^CD4^+^CD25^+^Foxp3^+^Treg cells in PBMCs in patients with oMG group was lower than HCs, and that of both patients with oMG and HCs were higher in patients with gMG. Balandina et al. also observed that CD4^+^CD25^+^Treg cells in MG patients had serious defects in immune regulation function and the expression of intracellular transcription factor Foxp3 was significantly reduced (27), which is represented in our experiments. Consequently, besides of the changes in the number of Treg cells, the impairment of immunosuppressive function of Treg cells caused by the decline of Treg cell function was also a key factor for the triggering of oMG and its progression into gMG.

At present, studies have divided Treg cells in PBMCs into some subsets by intracellular Foxp3 and cell surface CD45RA molecular expression levels. Hoffmann et al. found CD45RA^+^ Treg cells were the Treg cell subsets with the strongest inhibitory ability through *in vitro* experiments (28). Meanwhile, the initial Treg cell subsets expressing CD45RA could continuously express Foxp3 protein, but Long et al. found the immunosuppressive ability of CD4^+^CD25^hi^CD45RA^+^ Treg cells decreased after differentiation (29). The initial type of Treg enters the peripheral immune system from the thymus and is activated and differentiated into CD45RA^–^Foxp3^high^Treg (aTreg) cells after antigen stimulation, while the unstimulated type is differentiated into CD45RA^+^Foxp3^low^Treg (rTreg) cells. Both CD45RA^+^Foxp3^low^Treg cells and CD45RA^–^Foxp3^high^ Treg cells have strong inhibitory ability.

rTreg cells can be activated and transformed into a Treg cells under antigen stimulation. Otherwise, the proliferation of aTreg cells can also negatively inhibit the proliferation and activation of rTreg cells and further transform into aTreg cells (30). What’s more, aTreg cells will die soon after they are activated to exert inhibitory effect *in vivo*, which will promote rTreg cells to proliferate and transform into aTreg cells, so the two groups of cells can be in dynamic balance (30). Mohammad et al. found that the reduction of immunosuppressive regulation function after kidney transplantation was closely related to acute immune rejection, and compared with healthy control group and transplantation stable group, the proportion of aTreg cells and rTreg cells in peripheral blood of patients with immune function of immune rejection was significantly reduced (31). It is speculated that aTreg cells and rTreg cells are important reasons for the decreased immunosuppression ability of the body. Kohler et al. observed a significant decrease in CD45RA^+^Foxp3^low^ Treg cells in peripheral blood of MG patients compared with healthy volunteers, but no further subgroup analysis was performed (15). In this study, we found that the proportion of rTreg in CD3^+^CD4^+^CD25^+^Treg of PBMCs in both patients with gMG and oMG was lower than that in HCs, but the difference between patients with patients and gMG was not statistically significant. So, we found that the pathogenicity of rTreg lymphocytes and MG may be related to participating in the triggering process of oMG, but its association with oMG progression as the pathogenicity mechanism of gMG needs further study.

Miyara et al. has reported that both aTreg cells and rTregcells can play immunosuppressive function *in vitro* experiments, while only aTreg cells have immunosuppressive activity *in vivo* (10). Cheng et al. found in a clinical study that the percentage of aTreg cells in peripheral blood was increased ITP patients treated with glucocorticoids, and ITP patients with a higher percentage of aTreg cells newly diagnosed were more sensitive to glucocorticoids (32). Our study observed that the proportion of CD3^+^CD4^+^CD25^+^Treg in PBMCs of patients with oMG was higher than gMG patients, and the ratio of aTreg in both of the above-mentioned two groups was lower than HC group. These results suggest that aTreg lymphocytes are a significant factor in the triggering of oMG and its progression into immunosuppressive dysfunction of gMG pathogenesis.

CD45RA^–^Foxp3^low^Treg (n-s Treg) cells are a group of lymphocytes with no immunosuppressive function, but can secrete a variety of effector Tregs’ cytokines like IL-2, IL-4, and IL-17, which are closely related to the disease progression of oMG and gMG (26). It was found in this study that the proportion of n-sTreg cells in PBMCs of gMG patients was higher than HCs and oMG patients, and the proportion of n-sTreg cells in patients with oMG was higher than HCs. So, we thought that n-sTreg cells play an important role in the pathogenesis of MG and may be involved in the progression of oMG to gMG. In addition, pro-inflammatory Th17 cells affect the production of MG autoantibodies by changing the balance of cytokines (9). The previous studies have found that Treg cells are unstable in inflammatory state and can differentiate into the characteristic phenotype of effect or CD4^+^T cells (33). The over expression of CD4^+^ effect or T cells can lead to the occurrence and development of oMG disease. At the same time, some studies suggested that there was a correlation between n-sTreg cells and Th17 cells (10). Therefore, it is of great significance to further explore the role of the interaction between n-sTreg cells and Th17 cells in the triggering of oMG and its progression to gMG.

## Data Availability Statement

The original contributions presented in this study are included in the article/Supplementary Material, further inquiries can be directed to the corresponding author.

## Ethics Statement

The studies involving human participants were reviewed and approved by the Institutional Review Board of the First Affiliated Hospital of Nanchang University. The patients/participants provided their written informed consent to participate in this study.

## Author Contributions

XWu conceived and designed the experiments. JR wrote the manuscript. XWa, QC, and YJ performed the data analyses. WF and HH collected data and information. SL and LS helped to perform the analysis and followed up patients. All authors listed have made a substantial, direct, and intellectual contribution to the work and approved it for publication.

## Conflict of Interest

The authors declare that the research was conducted in the absence of any commercial or financial relationships that could be construed as a potential conflict of interest.

## Publisher’s Note

All claims expressed in this article are solely those of the authors and do not necessarily represent those of their affiliated organizations, or those of the publisher, the editors and the reviewers. Any product that may be evaluated in this article, or claim that may be made by its manufacturer, is not guaranteed or endorsed by the publisher.
