# Corrigendum: Comparison of peripheral blood regulatory T cells and functional subsets between ocular and generalized myasthenia gravis

**DOI:** 10.3389/fmed.2023.1235861

**Published:** 2023-06-15

**Authors:** Jie Rao, Siyu Li, Xinyu Wang, Qi Cheng, Yu Ji, Wenwen Fu, Hui Huang, Ling Shi, Xiaorong Wu

**Affiliations:** Department of Ophthalmology, The First Affiliated Hospital of Nanchang University, Nanchang, China

**Keywords:** ocular myasthenia gravis, regulatory T cell, subset, CD45RA, Foxp3

In the published article, an author name was incorrectly written as Qiyu Wang. The correct spelling is Xinyu Wang.

In the published article, there was an error. The author's initials in the Author contributions section were written incorrectly.

A correction has been made to Author contributions. This section previously stated:

XW conceived and designed the experiments. JR wrote the manuscript. QW, QC, and YJ performed the data analyses. WF and HH collected data and information. SL and LS helped to perform the analysis and followed up patients. All authors listed have made a substantial, direct, and intellectual contribution to the work, and approved it for publication.

The corrected section appears below:

## Author contributions

XWu conceived and designed the experiments. JR wrote the manuscript. XWa, QC, and YJ performed the data analyses. WF and HH collected data and information. SL and LS helped to perform the analysis and followed up patients. All authors listed have made a substantial, direct, and intellectual contribution to the work and approved it for publication.

The authors apologize for these errors and state that this does not change the scientific conclusions of the article in any way. The original article has been updated.

