# Supplementary material for: Comparison of Peripheral Blood Regulatory T Cells and Functional Subsets Between Ocular and Generalized Myasthenia Gravis
Source: Front Med (Lausanne). 2022 Jun 9;9:851808. doi: 10.3389/fmed.2022.851808 (PMC9218215; doi:10.3389/fmed.2022.851808)
Supplement: Supplementary file 2 [file Data_Sheet_2.PDF]

```
GET DATA
  /TYPE=XLS
  /FILE='C:\Users\10622\Desktop\Treg\\source data.xls'
  /SHEET=name 'Sheet1'
  /CELLRANGE=full
  /READNAMES=on
  /ASSUMEDSTRWIDTH=32767.
EXECUTE.
DATASET NAME amp;nbsp;WINDOW=FRONT.
EXAMINE VARIABLES=age BY group
  /PLOT BOXPLOT NPPLOT
  /COMPARE GROUPS
  /STATISTICS NONE
  /CINTERVAL 95
  /MISSING LISTWISE
  /NOTOTAL.
```

	16-1-2022 215826
	1
	<none>
	<none>
	<none>
N	58
	EXAMINE VARIABLES=age BY group<br>/PLOT BOXPLOT NPPLOT<br>/COMPARE GROUPS<br>/STATISTICS NONE<br>/CINTERVAL 95<br>/MISSING LISTWISE<br>/NOTOTAL.
	00 00:00:01.610
	00 00:00:01.099

[1]

group

	group	N		N		N	
age	HC group	19	100.0%	0	.0%	19	100.0%
	oMG group	18	100.0%	0	.0%	18	100.0%
	gMG group	21	100.0%	0	.0%	21	100.0%

		Kolmogorov-Smirnov [a]			Shapiro-Wilk		
	group		df	Sig.		df	Sig.
age	HC group	.090	19	.200*	.979	19	.924
	oMG group	.130	18	.200*	.959	18	.585
	gMG group	.107	21	.200*	.976	21	.861

a. Lilliefors
* .

# age

Q - Q

age Q-Q

group= HC group

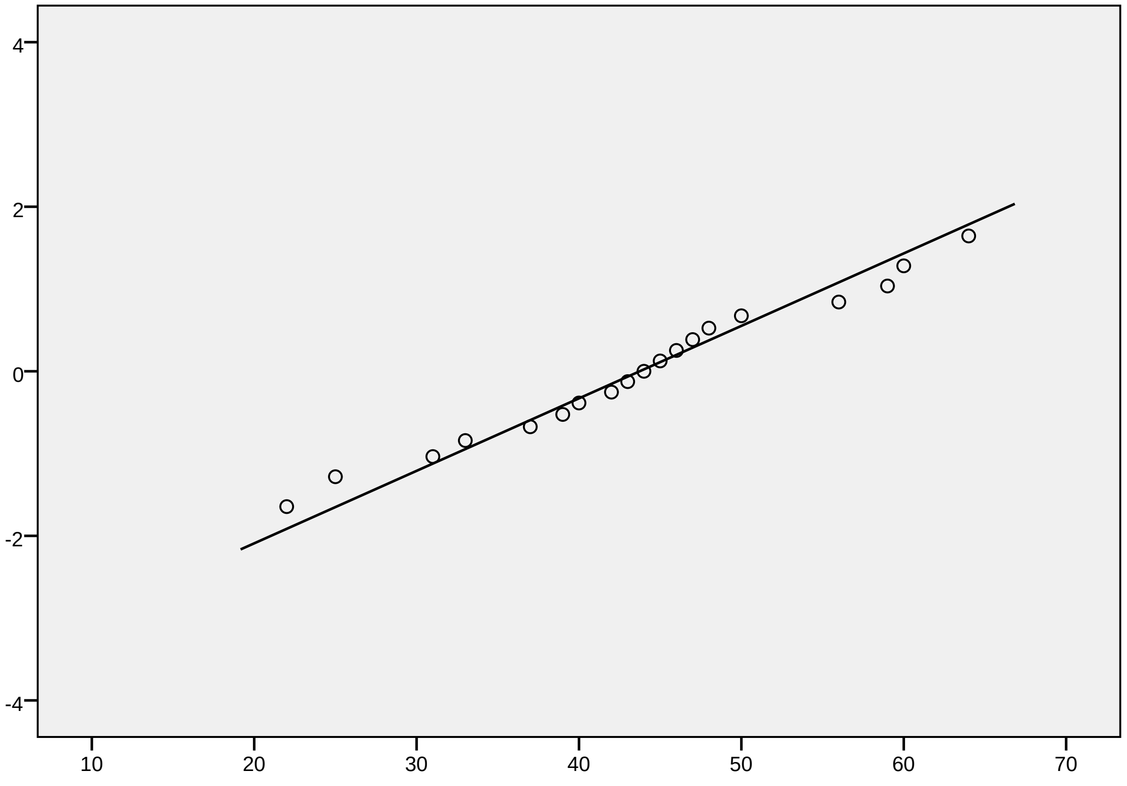

a g e   Q - Q

group= oMG group

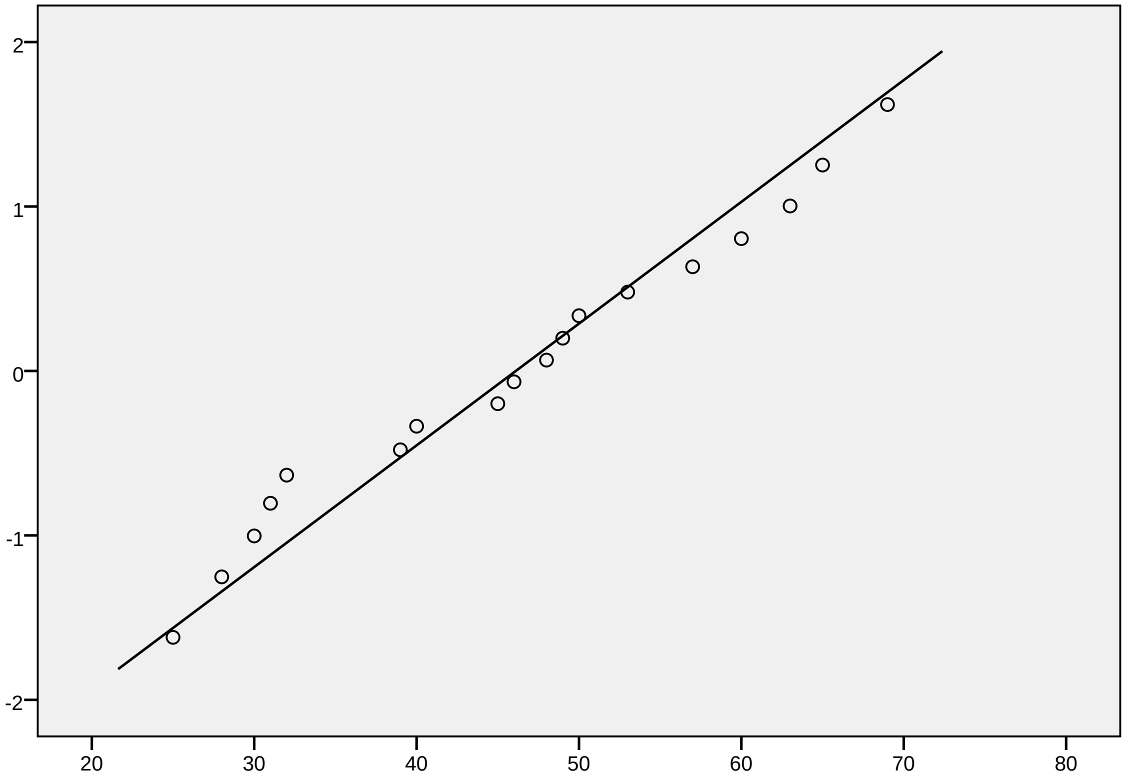

age Q-Q

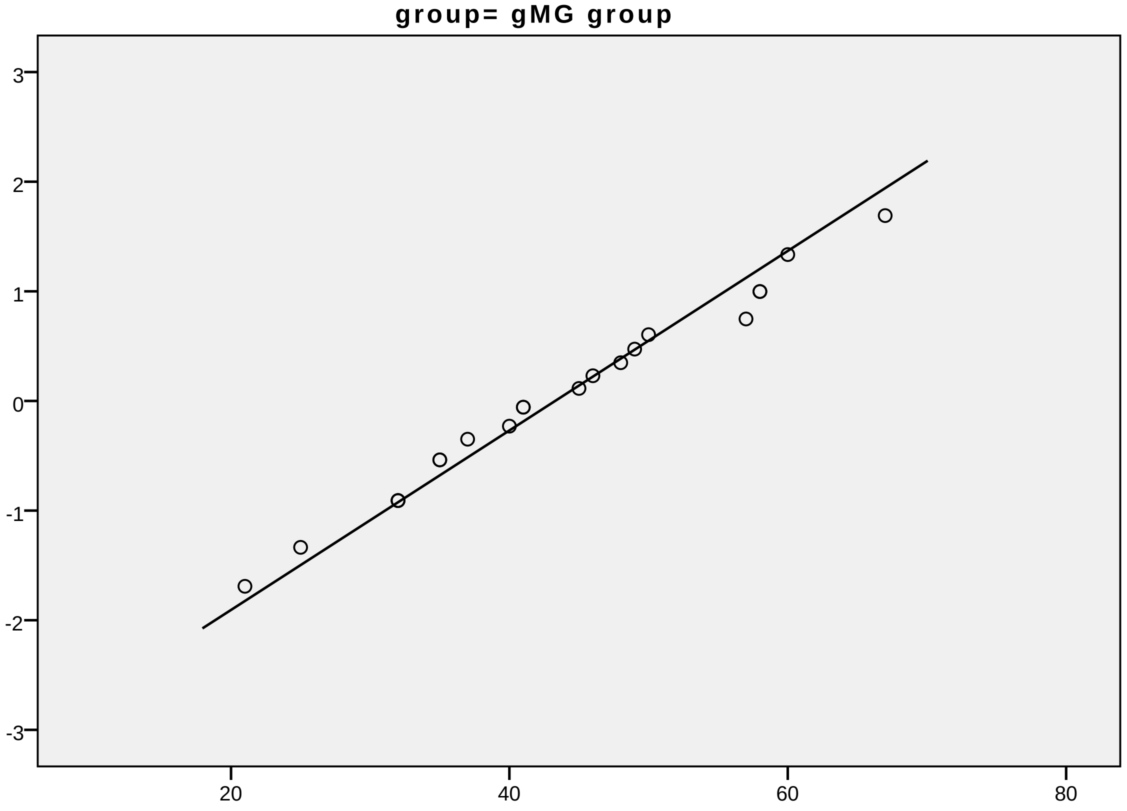

Q - Q

a g e Q - Q

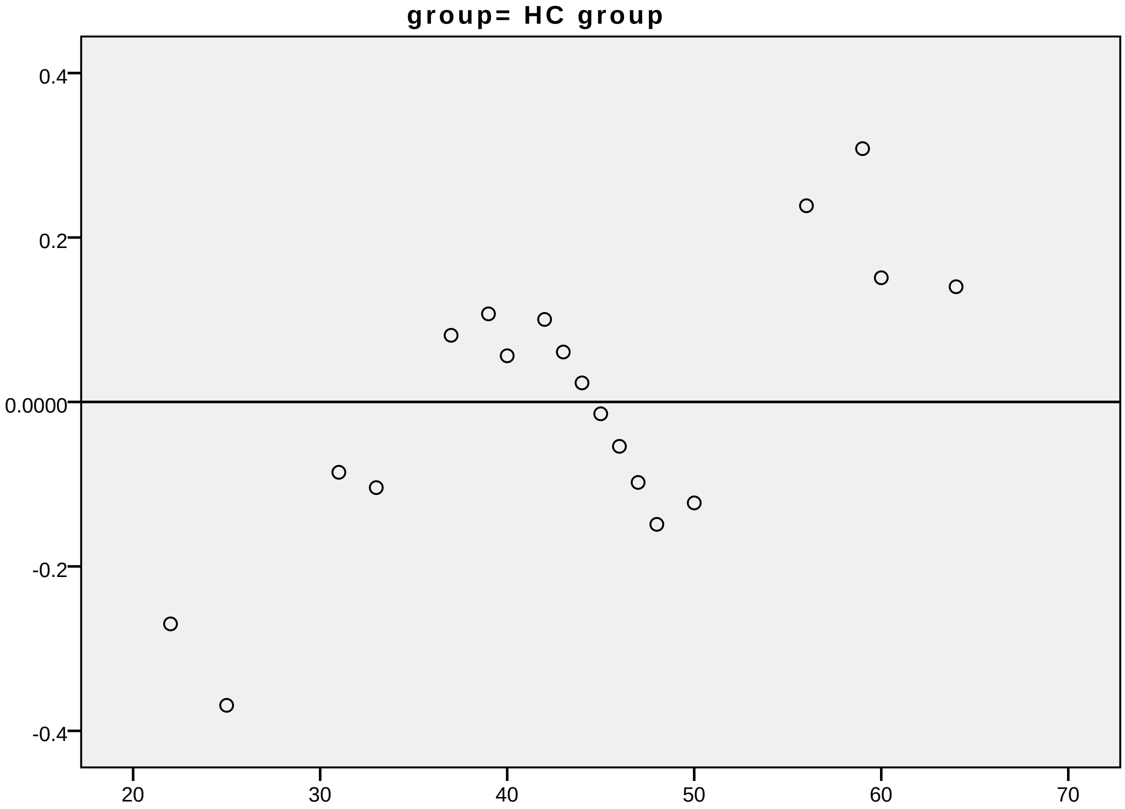

age Q-Q

group= oMG group

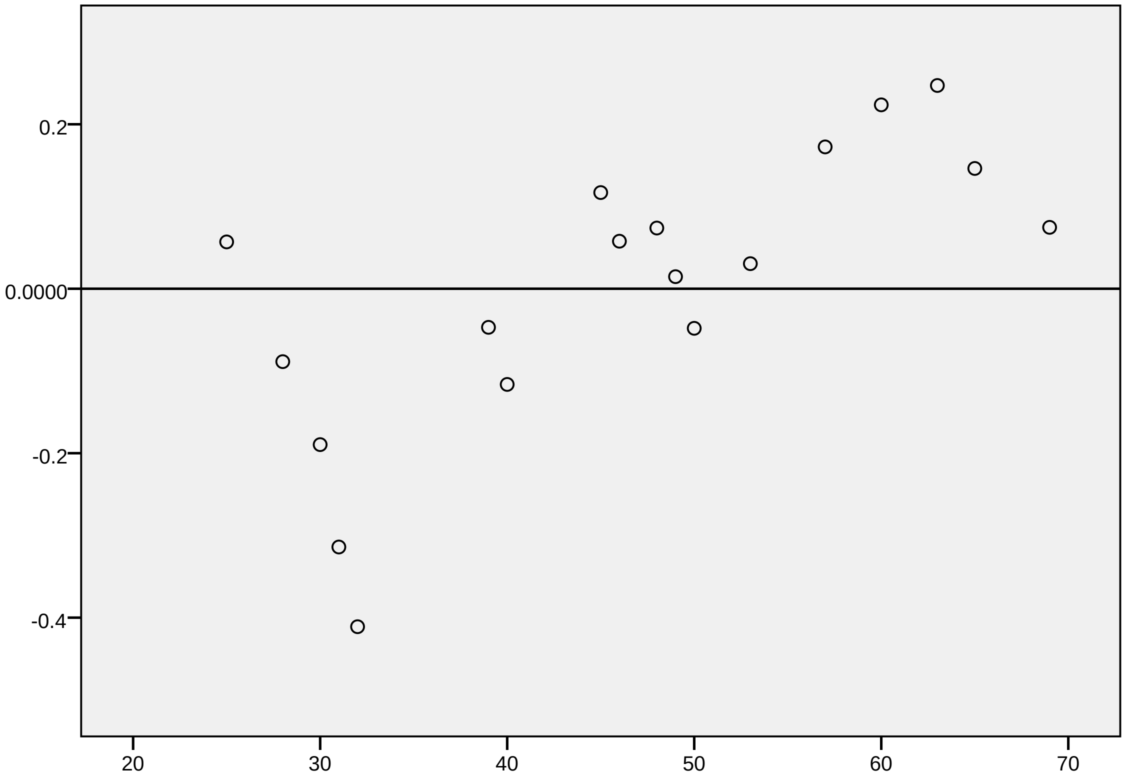

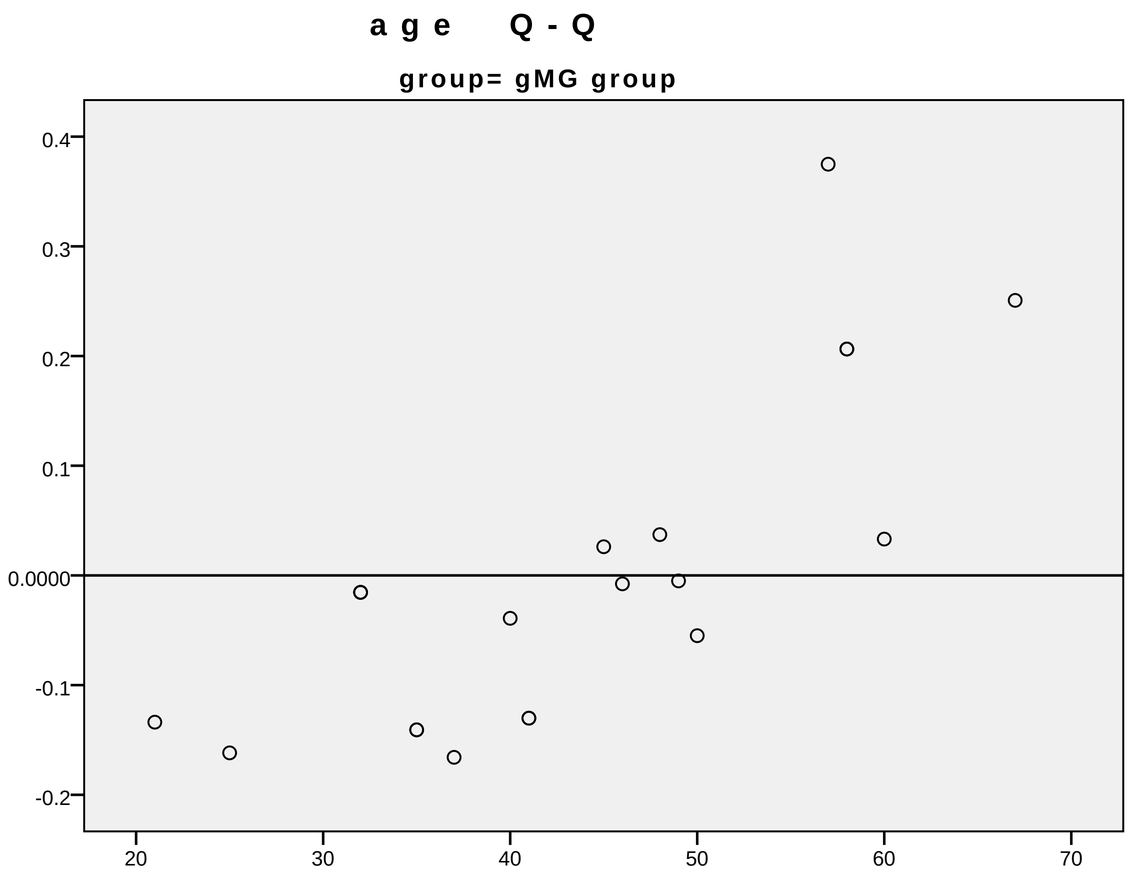
a g e Q - Q
group= gMG group
0.4
0.3
0.2
0.1
0.0000
-0.1
-0.2
20
30
40
50
60
70

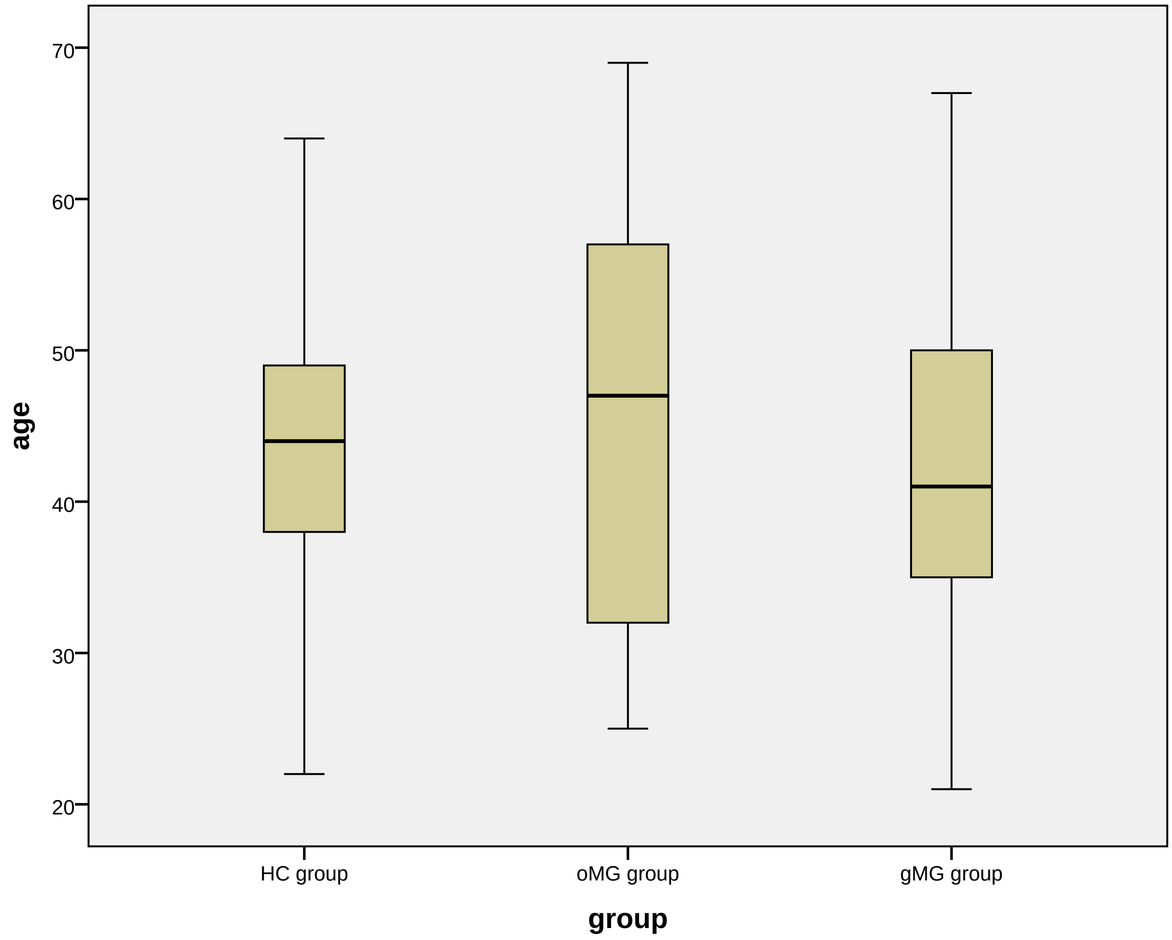


```
ONEWAY age BY group
  /STATISTICS DESCRIPTIVES HOMOGENEITY WELCH
  /PLOT MEANS
  /MISSING ANALYSIS
  /POSTHOC=TUKEY GH ALPHA(0.05).
```

	16-1-2022 215855
	1
	<none>
	<none>
	<none>
N	58
	ONEWAY age BY group<br>/STATISTICS DESCRIPTIVES HOMOGENEITY WELCH<br>/PLOT MEANS<br>/MISSING ANALYSIS<br>/POSTHOC=TUKEY GH ALPHA (0.05).
	00 00:00:00.125
	00 00:00:00.131

[1]

age

	N				9 5 %	
HC group	19	43.74	11.352	2.604	38.27	49.21
oMG group	18	46.11	13.508	3.184	39.39	52.83
gMG group	21	43.29	12.215	2.666	37.73	48.85
	58	44.31	12.206	1.603	41.10	47.52

age

HC group	22	64
oMG group	25	69
gMG group	21	67
	21	69

age

Levene	df1	df2	
.550	2	55	.580

ANOVA

age

		df		F	
	86.666	2	43.333	.284	.754
	8405.748	55	152.832		
	8492.414	57			

age

	[a]	df1	df2	
Welch	.250	2	35.959	.780

a . F

: a g e

	(I) group	(J) group	(I - J)		
Tukey HSD	HC group	oMG group	-2.374	4.066	.829
		gMG group	.451	3.914	.993
	oMG group	HC group	2.374	4.066	.829
		gMG group	2.825	3.971	.758
	gMG group	HC group	-.451	3.914	.993
		oMG group	-2.825	3.971	.758
Games-Howell	HC group	oMG group	-2.374	4.113	.833
		gMG group	.451	3.727	.992
	oMG group	HC group	2.374	4.113	.833
		gMG group	2.825	4.152	.776
	gMG group	HC group	-.451	3.727	.992
		oMG group	-2.825	4.152	.776

: a g e

	(I) group	(J) group	9 5 %	
Tukey HSD	HC group	oMG group	-12.17	7.42
		gMG group	-8.98	9.88
	oMG group	HC group	-7.42	12.17
		gMG group	-6.74	12.39
	gMG group	HC group	-9.88	8.98
		oMG group	-12.39	6.74
Games-Howell	HC group	oMG group	-12.46	7.71
		gMG group	-8.64	9.54
	oMG group	HC group	-7.71	12.46
		gMG group	-7.34	12.99
	gMG group	HC group	-9.54	8.64
		oMG group	-12.99	7.34

age

	group	N	alpha = 0.05<br>1
Tukey HSD[a,b]	gMG group	21	43.29
	HC group	19	43.74
	oMG group	18	46.11
			.759

a . = 1 9 . 2 5 5
b .
I

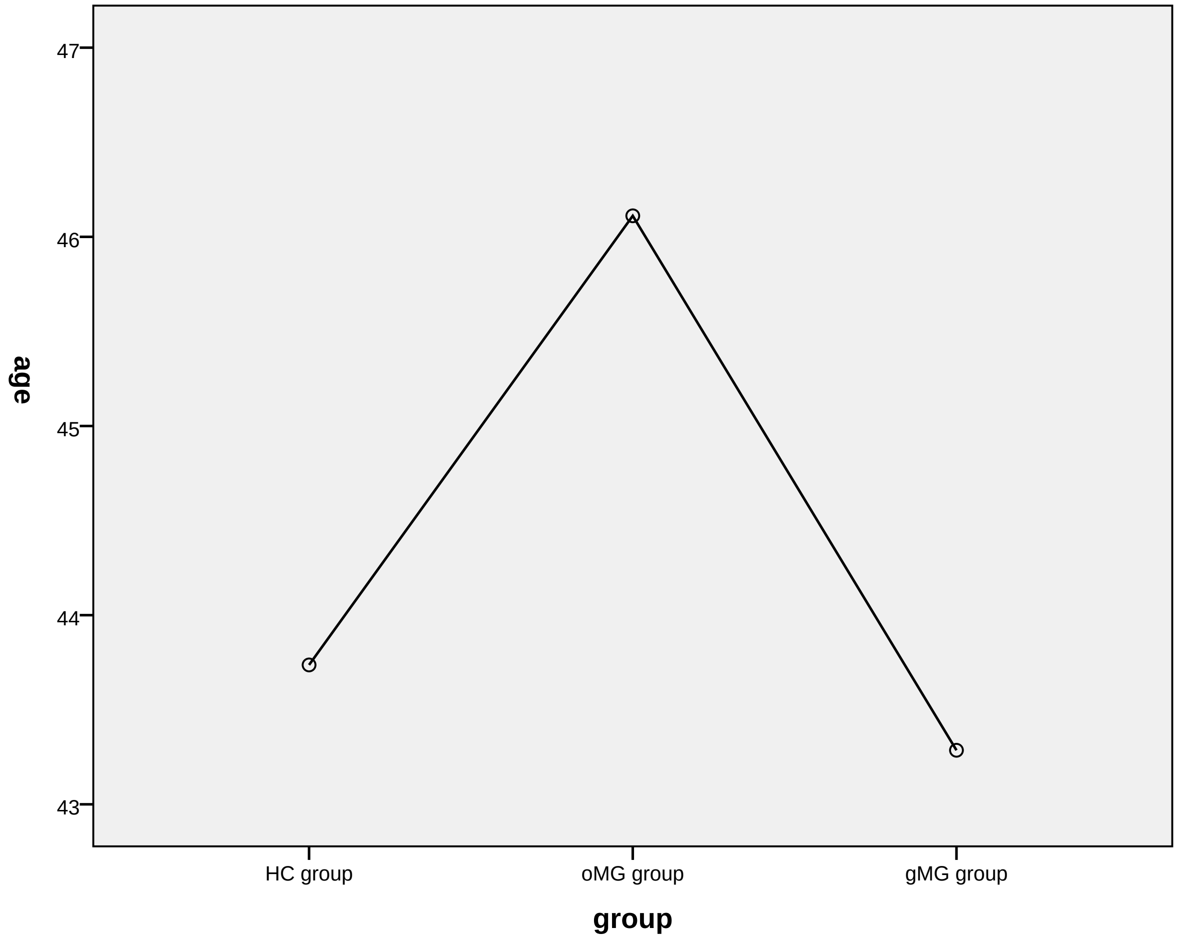


```
EXAMINE VARIABLES=CD3CD4 BY group
  /PLOT BOXPLOT NPPLOT
  /COMPARE GROUPS
  /STATISTICS NONE
  /CINTERVAL 95
  /MISSING LISTWISE
  /NOTOTAL.
```

	16-1-2022 215913
	1
	<none>
	<none>
	<none>
N	58
	EXAMINE VARIABLES=CD3CD4 BY group /PLOT BOXPLOT NPPLOT /COMPARE GROUPS /STATISTICS NONE /CINTERVAL 95 /MISSING LISTWISE /NOTOTAL.
	00 00:00:00.782
	00 00:00:00.750

[1]

group

	group	N		N		N	
CD3CD4	HC group	19	100.0%	0	.0%	19	100.0%
	oMG group	18	100.0%	0	.0%	18	100.0%
	gMG group	21	100.0%	0	.0%	21	100.0%

		Kolmogorov-Smirnov[a]			Shapiro-Wilk		
	group		df	Sig.		df	Sig.
CD3CD4	HC group	.140	19	.200*	.971	19	.798
	oMG group	.113	18	.200*	.976	18	.902
	gMG group	.144	21	.200*	.959	21	.499

a. Lilliefors
* .

CD3CD4

Q - Q

CD3CD4 Q-Q

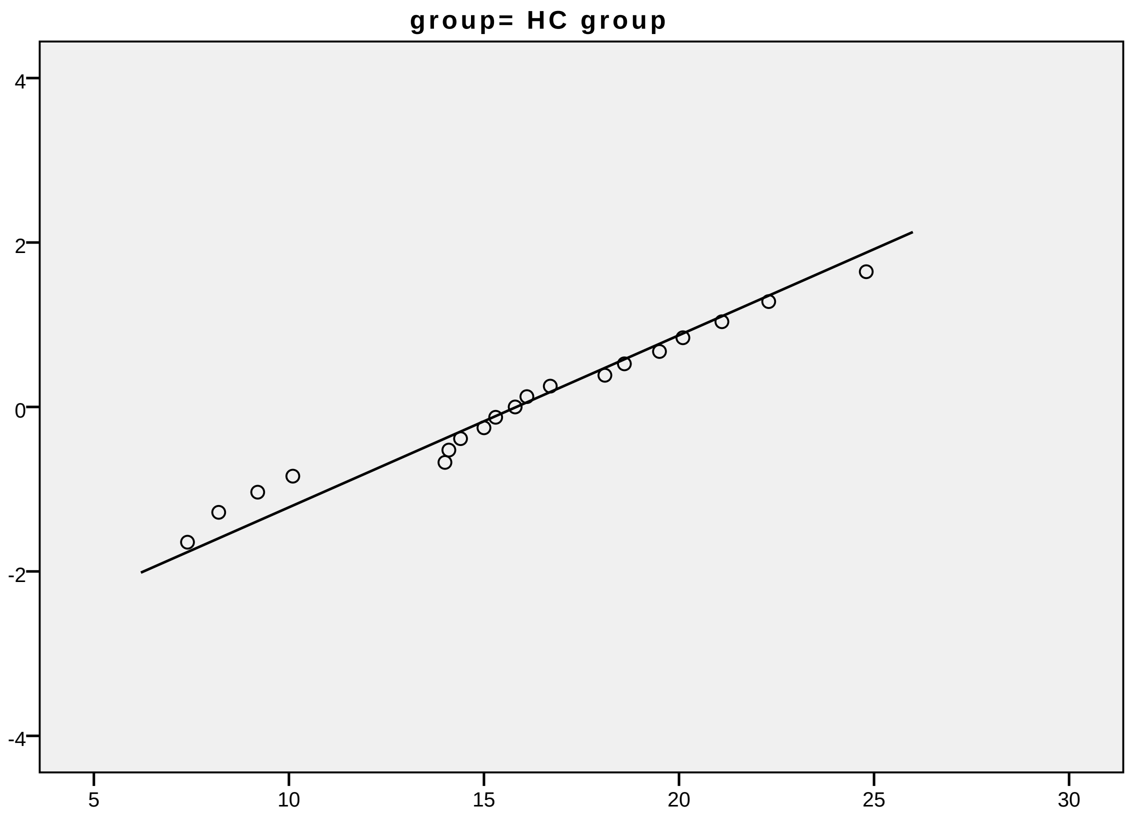

C D 3 C D 4 Q - Q

group= oMG group

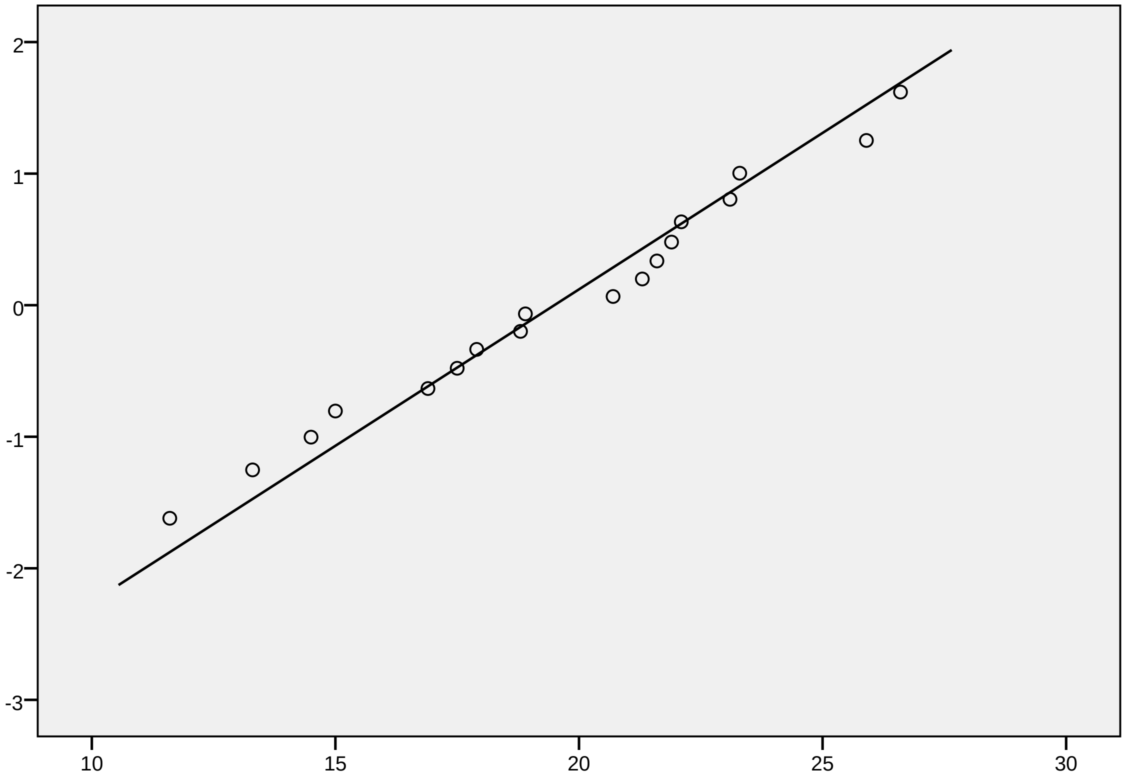

C D 3 C D 4   Q - Q

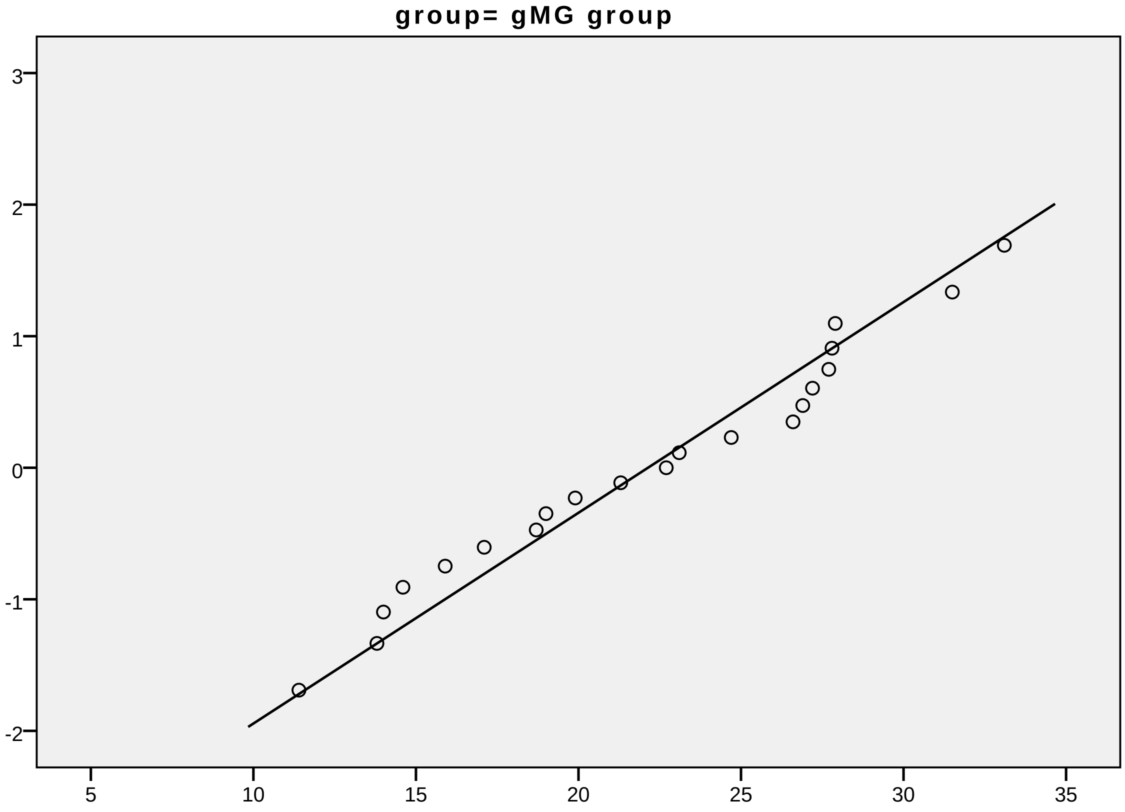

Q - Q

C D 3 C D 4 Q - Q

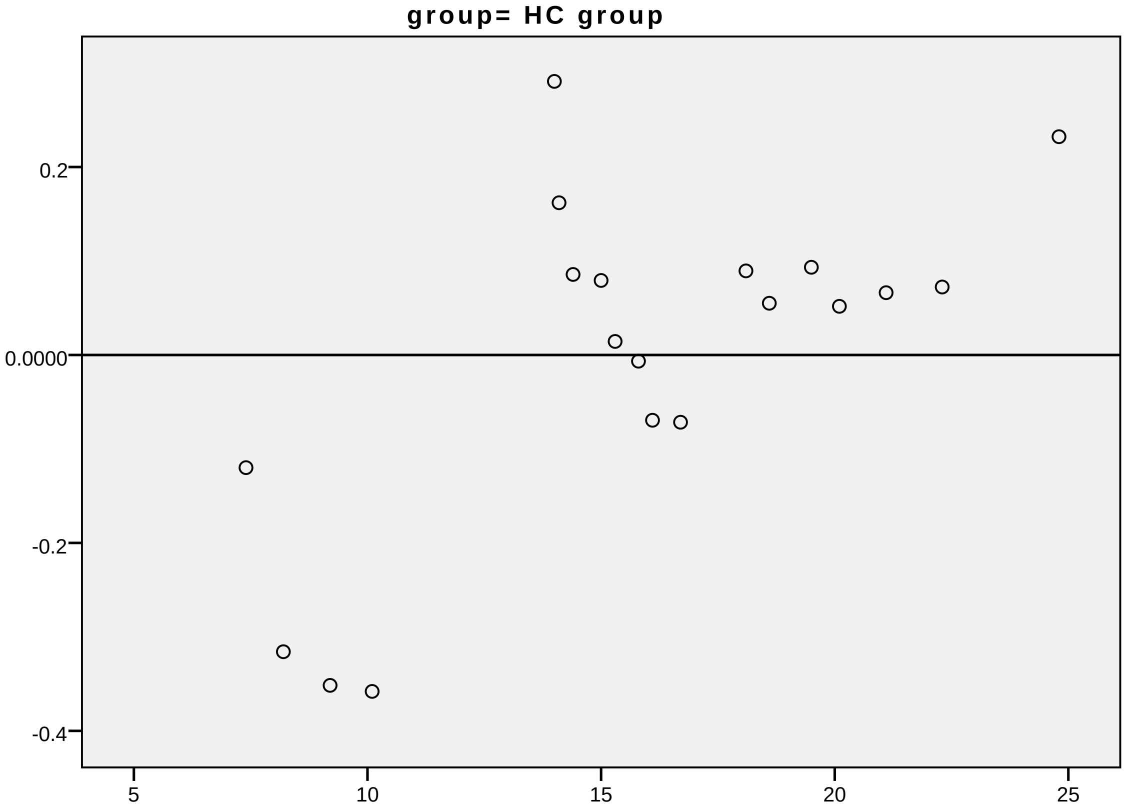

C D 3 C D 4 Q - Q

group= oMG group

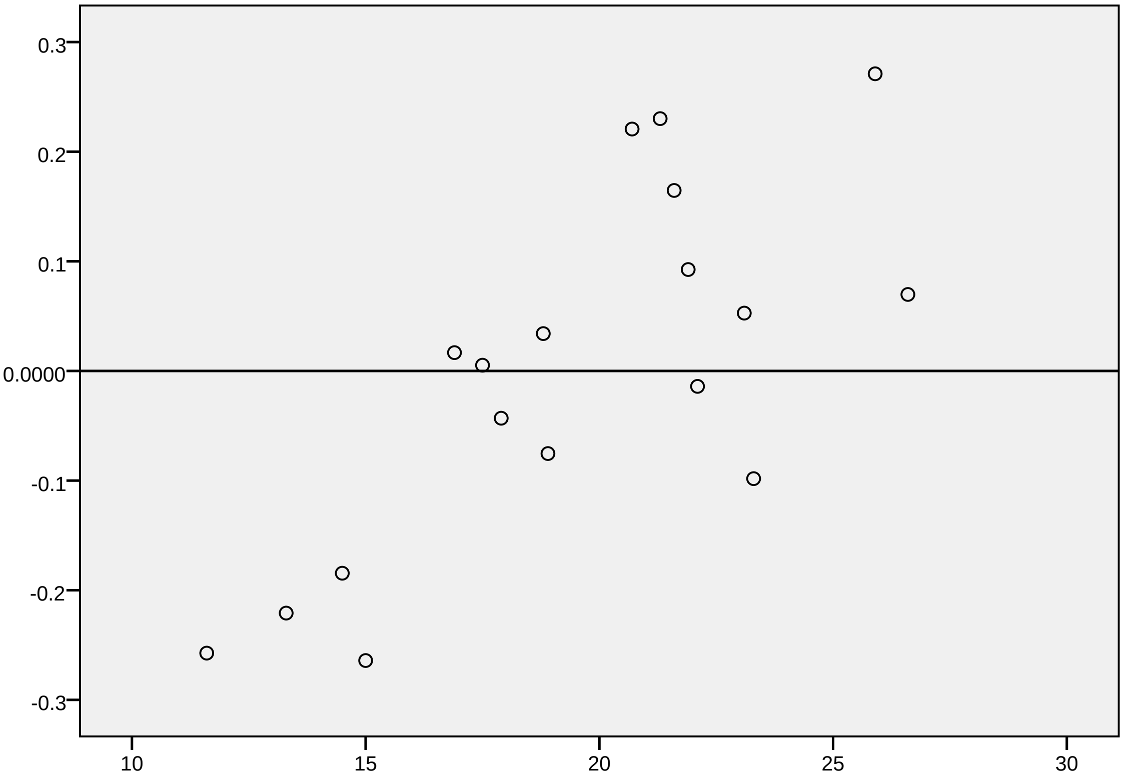

C D 3 C D 4 Q - Q

group= gMG group

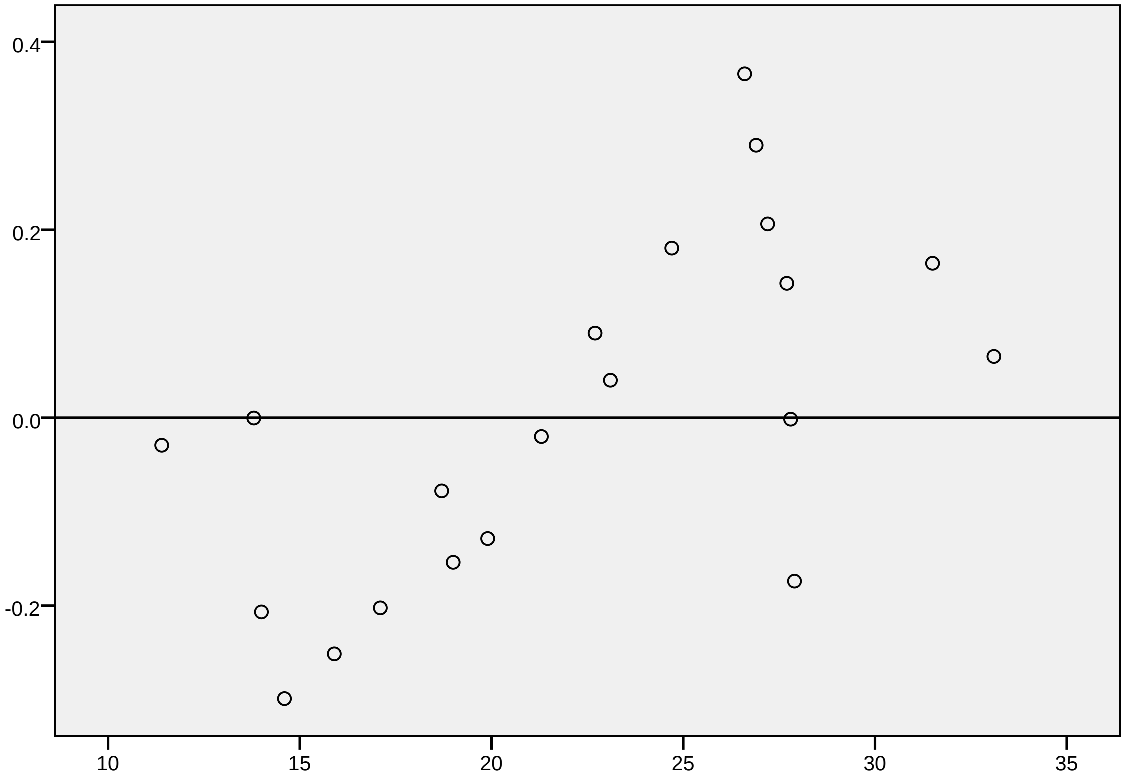

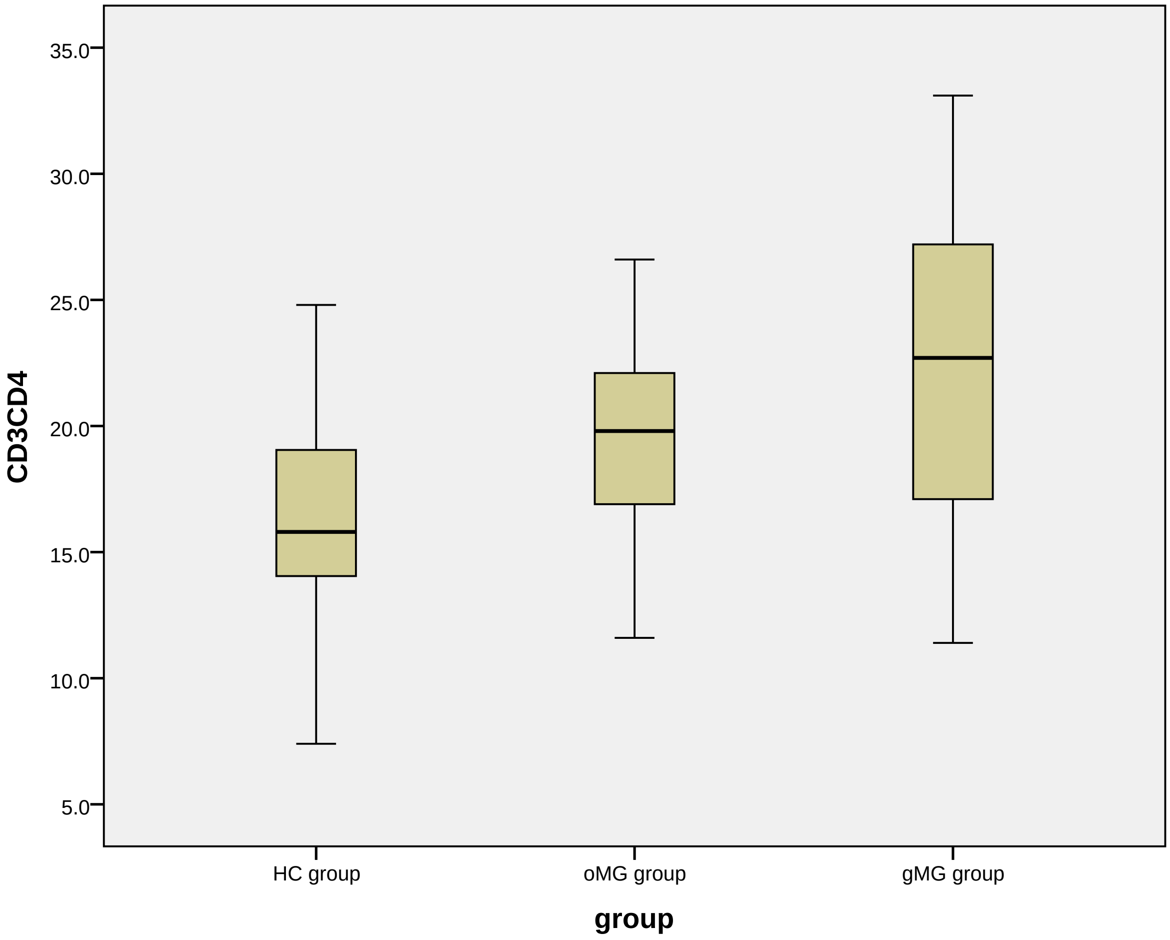


```
ONEWAY CD3CD4 BY group
  /STATISTICS DESCRIPTIVES HOMOGENEITY WELCH
  /PLOT MEANS
  /MISSING ANALYSIS
  /POSTHOC=TUKEY GH ALPHA(0.05).
```

	16-1-2022 215920
	1
	<none>
	<none>
	<none>
N	58
	ONEWAY CD3CD4 BY group /STATISTICS DESCRIPTIVES HOMOGENEITY WELCH /PLOT MEANS /MISSING ANALYSIS /POSTHOC=TUKEY GH ALPHA (0.05).
	00 00:00:00.125
	00 00:00:00.125

[1]

CD3CD4

	N				95%	
HC group	19	15.832	4.7777	1.0961	13.529	18.134
oMG group	18	19.494	4.2054	.9912	17.403	21.586
gMG group	21	22.138	6.2433	1.3624	19.296	24.980
	58	19.252	5.7573	.7560	17.738	20.766

CD3CD4

HC group	7.4	24.8
oMG group	11.6	26.6
gMG group	11.4	33.1
	7.4	33.1

CD3CD4

Levene	df1	df2	
2.622	2	55	.082

ANOVA

CD3CD4

		df		F	
	398.265	2	199.132	7.345	.001
	1491.100	55	27.111		
	1889.365	57			

CD3CD4

	[a]	df1	df2	
Welch	6.790	2	36.553	.003

a . F

: C D 3 C D 4

	(I) group	(J) group	(I - J)		
Tukey HSD	HC group	oMG group	-3.6629	1.7126	.091
		gMG group	-6.3065*	1.6486	.001
	oMG group	HC group	3.6629	1.7126	.091
		gMG group	-2.6437	1.6725	.262
	gMG group	HC group	6.3065*	1.6486	.001
		oMG group	2.6437	1.6725	.262
Games-Howell	HC group	oMG group	-3.6629*	1.4778	.047
		gMG group	-6.3065*	1.7486	.003
	oMG group	HC group	3.6629*	1.4778	.047
		gMG group	-2.6437	1.6848	.272
	gMG group	HC group	6.3065*	1.7486	.003
		oMG group	2.6437	1.6848	.272

: C D 3 C D 4

	(I) group	(J) group	9 5 %	
Tukey HSD	HC group	oMG group	-7.788	.462
		gMG group	-10.278	-2.335
	oMG group	HC group	-.462	7.788
		gMG group	-6.672	1.385
	gMG group	HC group	2.335	10.278
		oMG group	-1.385	6.672
Games-Howell	HC group	oMG group	-7.280	-.045
		gMG group	-10.575	-2.038
	oMG group	HC group	.045	7.280
		gMG group	-6.766	1.479
	gMG group	HC group	2.038	10.575
		oMG group	-1.479	6.766

* . 0 . 0 5

CD3CD4

	group	N	alpha = 0.05	
			1	2
Tukey HSD[a,b]	HC group	19	15.832	
	oMG group	18	19.494	19.494
	gMG group	21		22.138
			.083	.265

a . = 1 9 . 2 5 5
b . I

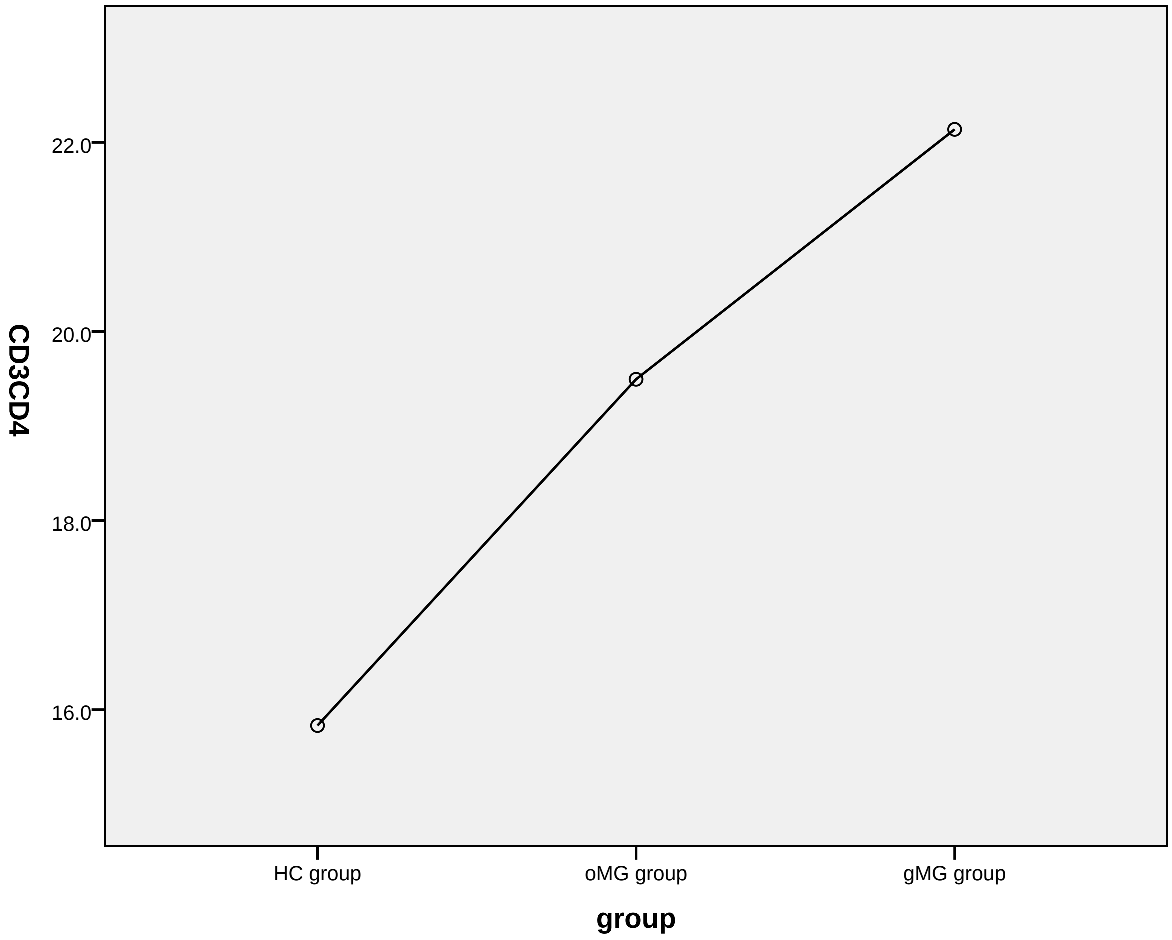


```
EXAMINE VARIABLES=CD4Cd25 BY group
  /PLOT BOXPLOT NPPLOT
  /COMPARE GROUPS
  /STATISTICS NONE
  /CINTERVAL 95
  /MISSING LISTWISE
  /NOTOTAL.
```

	16-1-2022 215936
	1
	<none>
	<none>
	<none>
N	58
	EXAMINE VARIABLES=CD4Cd25 BY group<br>/PLOT BOXPLOT NPPLOT<br>/COMPARE GROUPS<br>/STATISTICS NONE<br>/CINTERVAL 95<br>/MISSING LISTWISE<br>/NOTOTAL.
	00 00:00:00.750
	00 00:00:00.729

[1]

group

	group	N		N		N	
CD4Cd25	HC group	19	100.0%	0	.0%	19	100.0%
	oMG group	18	100.0%	0	.0%	18	100.0%
	gMG group	21	100.0%	0	.0%	21	100.0%

		Kolmogorov-Smirnov[a]			Shapiro-Wilk		
	group		df	Sig.		df	Sig.
CD4Cd25	HC group	.131	19	.200*	.942	19	.292
	oMG group	.129	18	.200*	.930	18	.194
	gMG group	.141	21	.200*	.943	21	.245

a. Lilliefors
* .

CD4Cd25

Q - Q

CD4Cd25 Q-Q

group= HC group

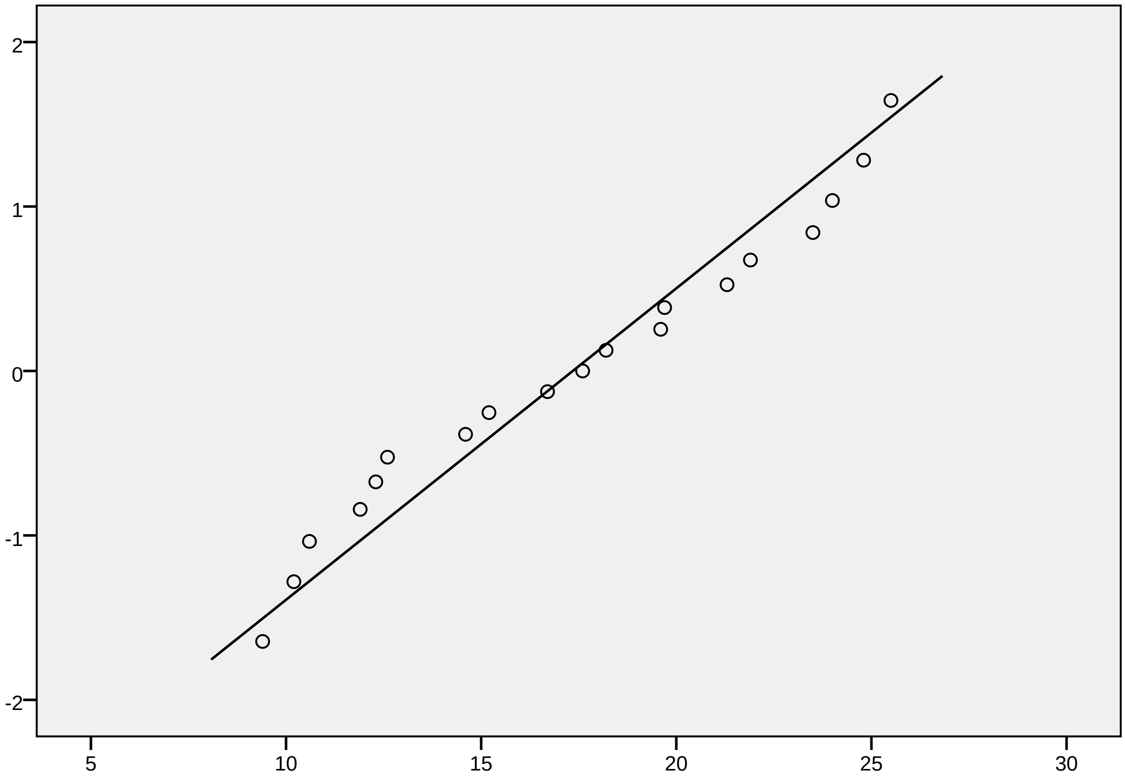

CD4Cd25 Q-Q

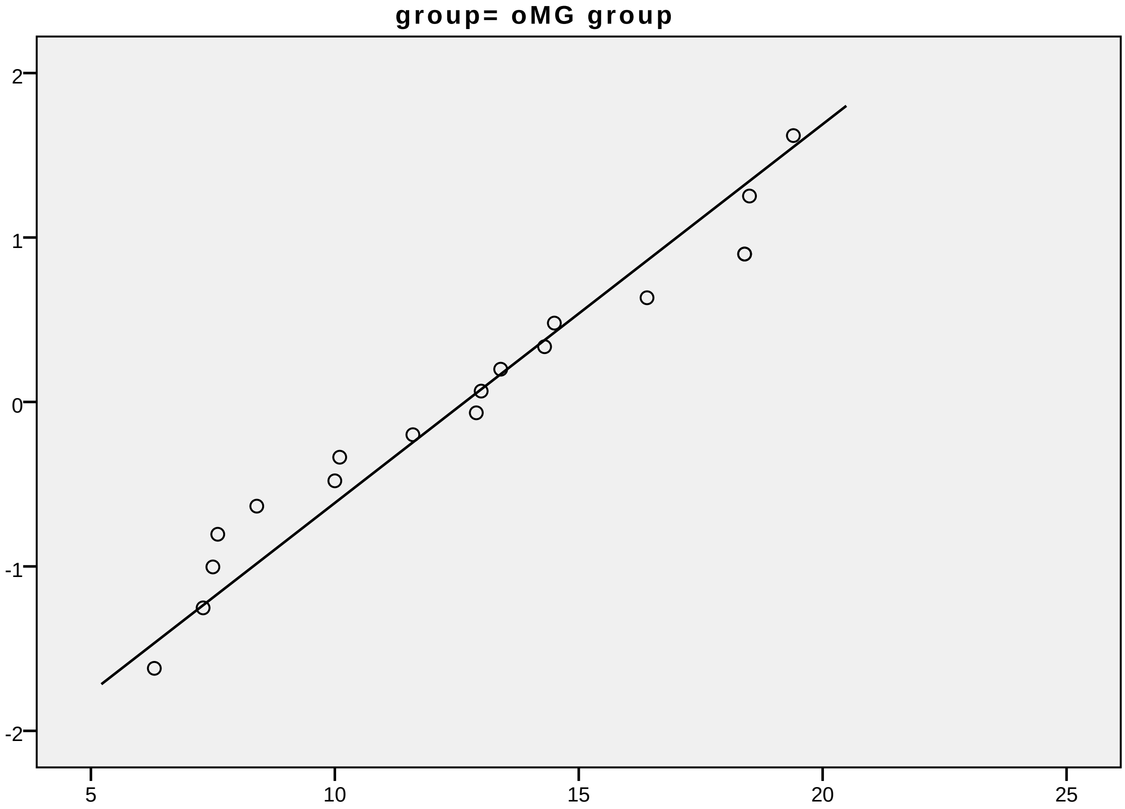

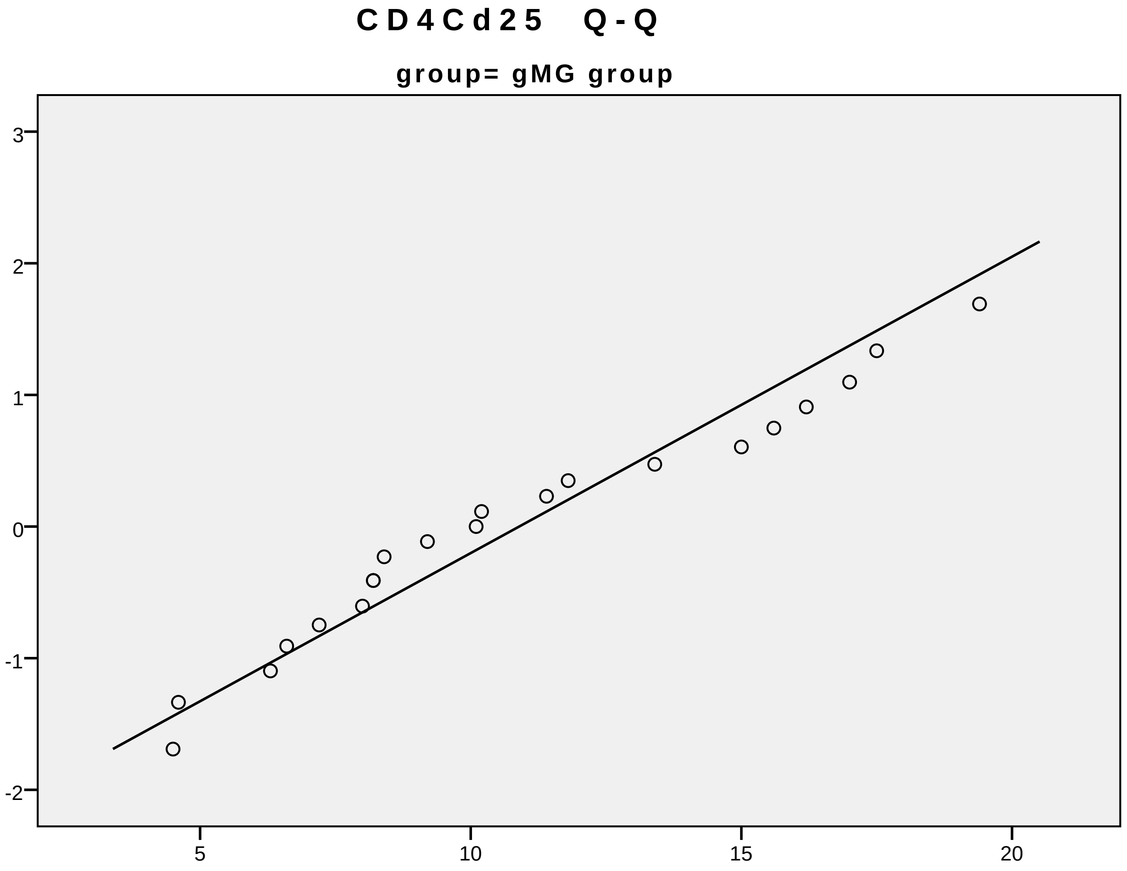

Q - Q

CD4Cd25 Q-Q

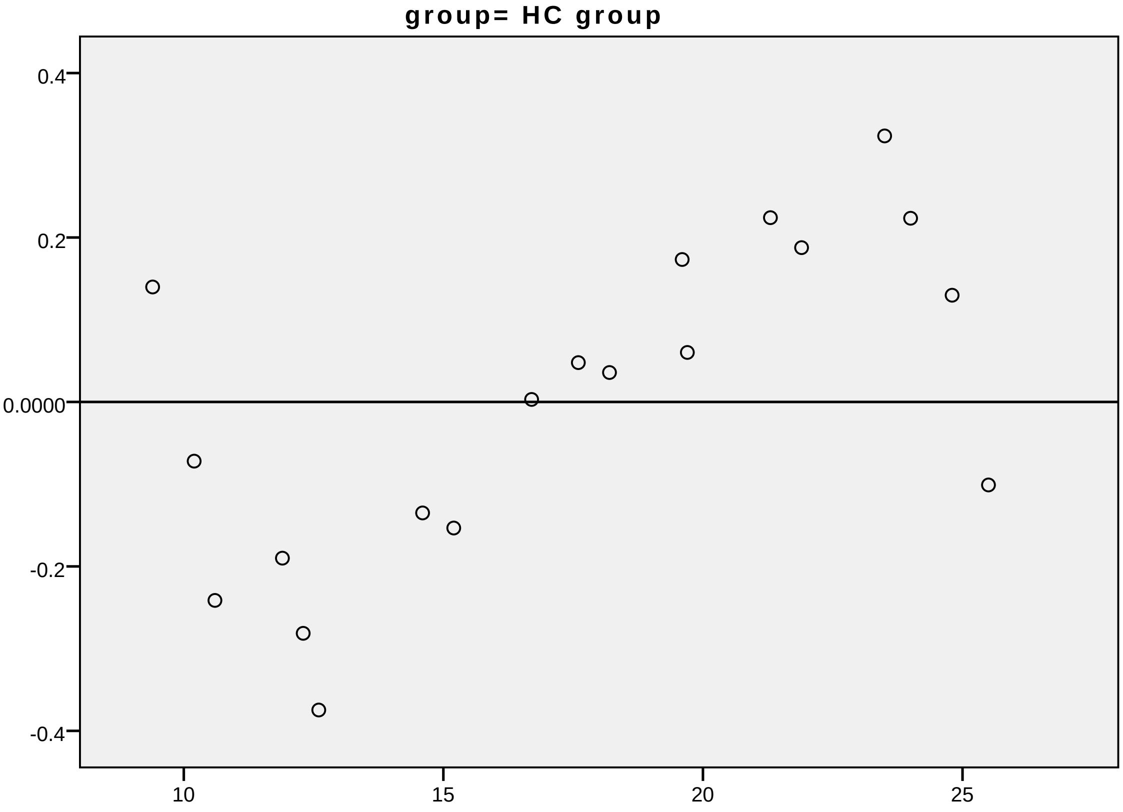

C D 4 C d 2 5 Q - Q

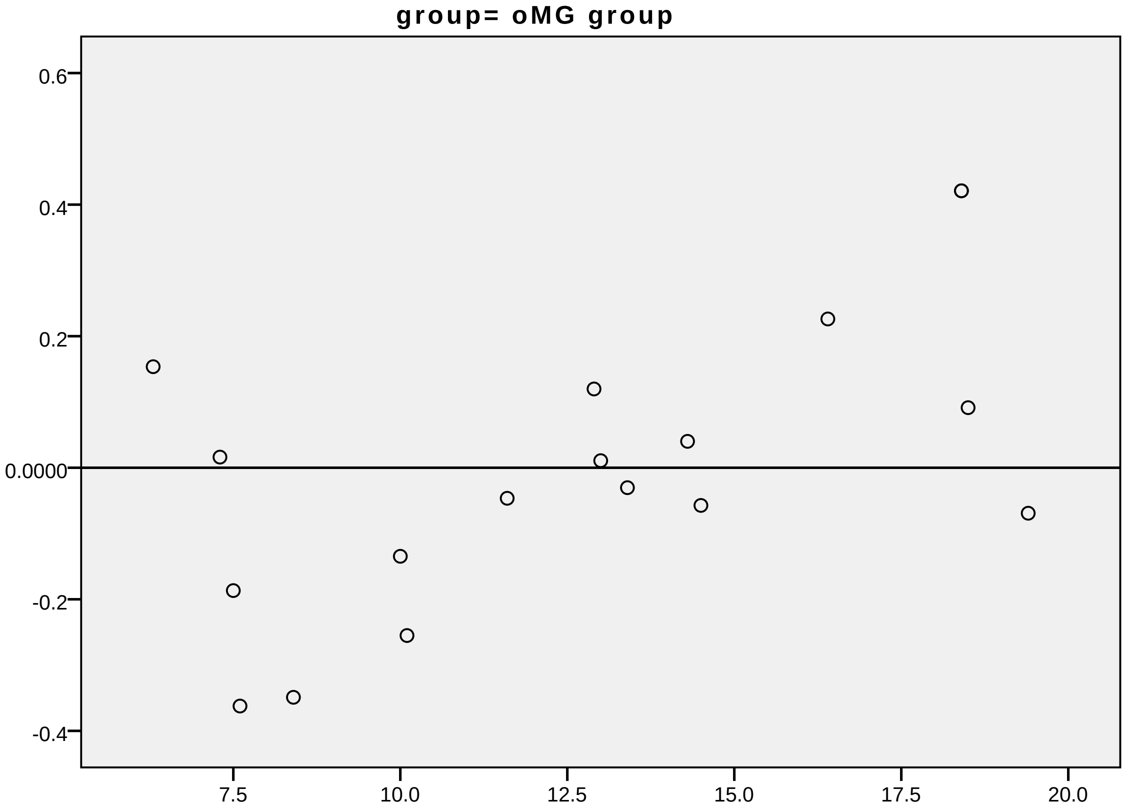

CD4Cd25 Q-Q

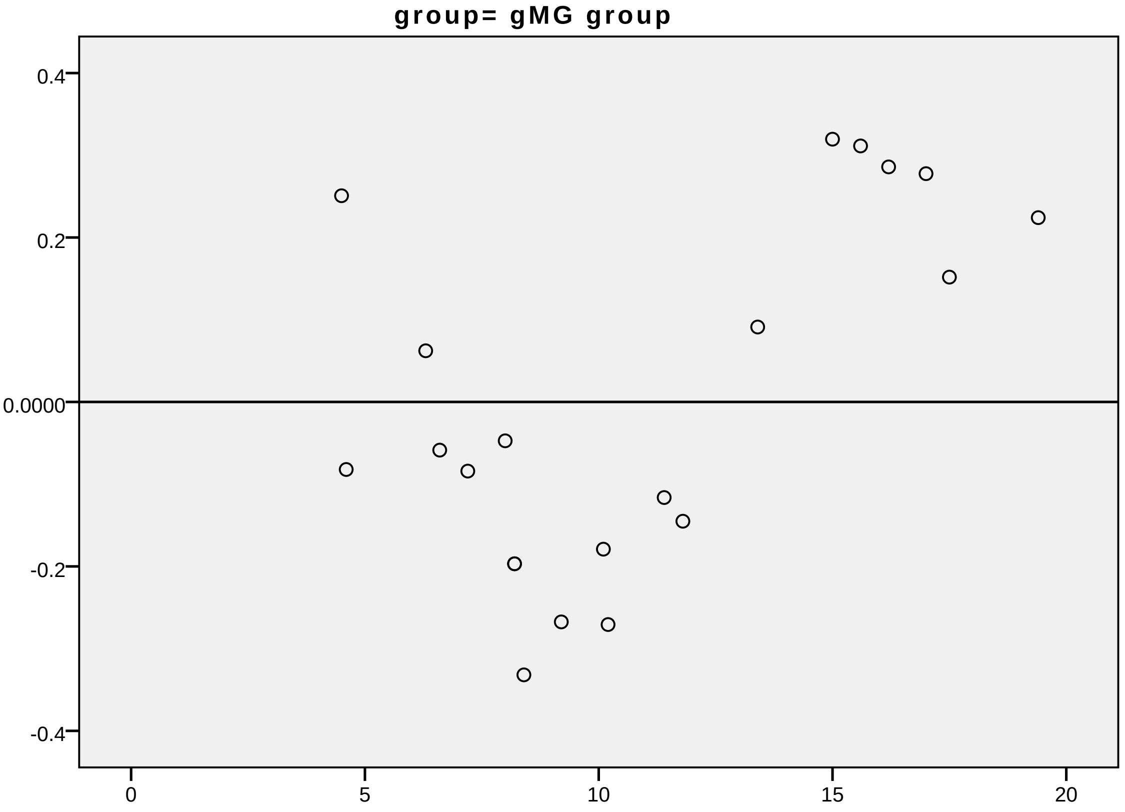

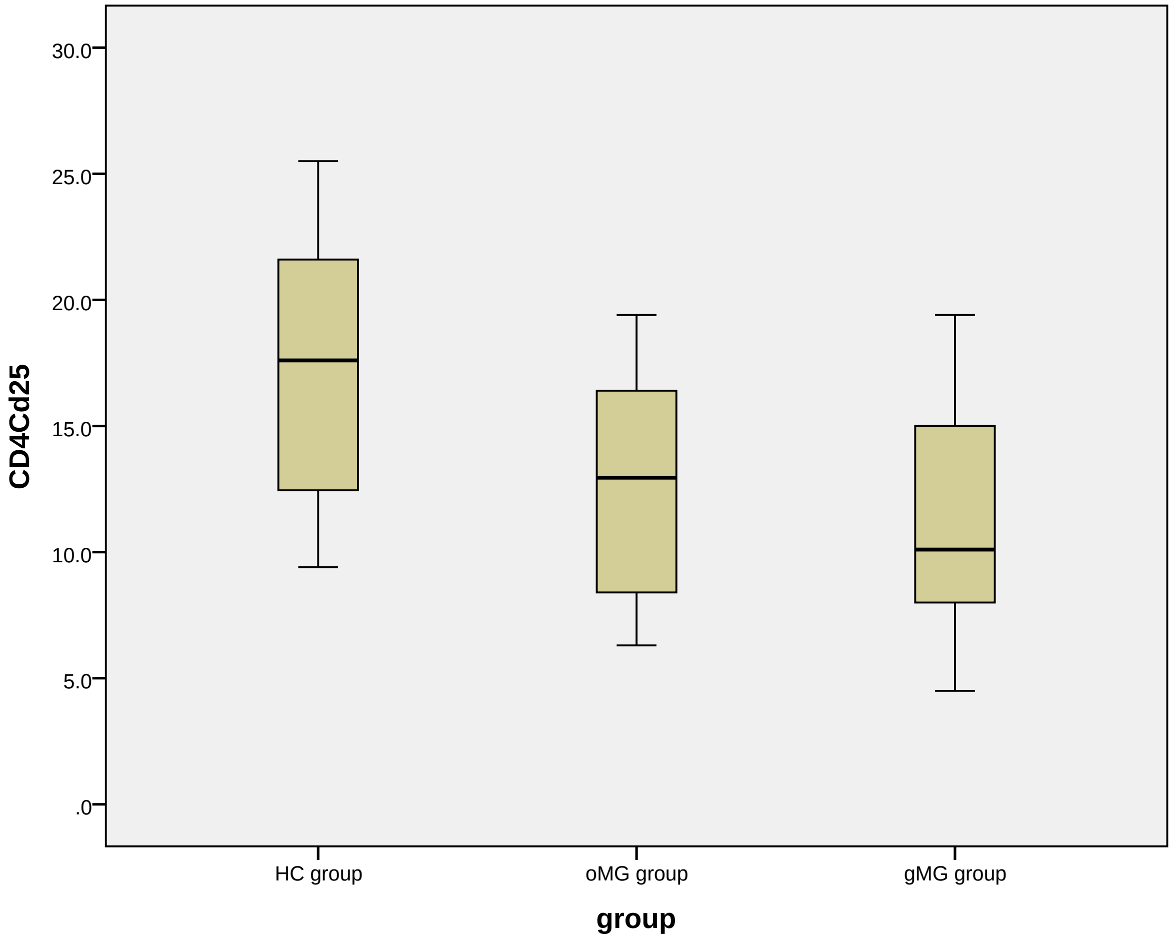


```
ONEWAY CD4Cd25 BY group
  /STATISTICS DESCRIPTIVES HOMOGENEITY WELCH
  /PLOT MEANS
  /MISSING ANALYSIS
  /POSTHOC=TUKEY GH ALPHA(0.05).
```

	16-1-2022 215943
	1
	<none>
	<none>
	<none>
N	58
	ONEWAY CD4Cd25 BY group<br>/STATISTICS DESCRIPTIVES HOMOGENEITY WELCH<br>/PLOT MEANS<br>/MISSING ANALYSIS<br>/POSTHOC=TUKEY GH ALPHA (0.05).
	00 00:00:00.125
	00 00:00:00.130

[1]

CD4Cd25

	N				9 5 %	
HC group	19	17.347	5.2805	1.2114	14.802	19.892
oMG group	18	12.667	4.3421	1.0235	10.507	14.826
gMG group	21	10.895	4.4417	.9693	8.873	12.917
	58	13.559	5.3852	.7071	12.143	14.975

CD4Cd25

HC group	9.4	25.5
oMG group	6.3	19.4
gMG group	4.5	19.4
	4.5	25.5

CD4Cd25

Levene	df1	df2	
.759	2	55	.473

ANOVA

CD4Cd25

		df		F	
	436.024	2	218.012	9.853	.000
	1216.997	55	22.127		
	1653.021	57			

CD4Cd25

	[a]	df1	df2	
Welch	8.660	2	36.039	.001

a . F

: C D 4 C d 2 5

	(I) group	(J) group	(I - J)		
Tukey HSD	HC group	oMG group	4.6807*	1.5472	.010
		gMG group	6.4521*	1.4894	.000
	oMG group	HC group	-4.6807*	1.5472	.010
		gMG group	1.7714	1.5109	.475
	gMG group	HC group	-6.4521*	1.4894	.000
		oMG group	-1.7714	1.5109	.475
Games-Howell	HC group	oMG group	4.6807*	1.5859	.015
		gMG group	6.4521*	1.5515	.001
	oMG group	HC group	-4.6807*	1.5859	.015
		gMG group	1.7714	1.4096	.428
	gMG group	HC group	-6.4521*	1.5515	.001
		oMG group	-1.7714	1.4096	.428

: C D 4 C d 2 5

	(I) group	(J) group	9 5 %	
Tukey HSD	HC group	oMG group	.954	8.408
		gMG group	2.865	10.040
	oMG group	HC group	-8.408	-.954
		gMG group	-1.868	5.411
	gMG group	HC group	-10.040	-2.865
		oMG group	-5.411	1.868
Games-Howell	HC group	oMG group	.796	8.565
		gMG group	2.657	10.247
	oMG group	HC group	-8.565	-.796
		gMG group	-1.673	5.216
	gMG group	HC group	-10.247	-2.657
		oMG group	-5.216	1.673

* . 0 . 0 5

CD4Cd25

	group	N	alpha = 0.05	
			1	2
Tukey HSD [a,b]	gMG group	21	10.895	
	oMG group	18	12.667	
	HC group	19		17.347
			.477	1.000

a . = 1 9 . 2 5 5
b . I

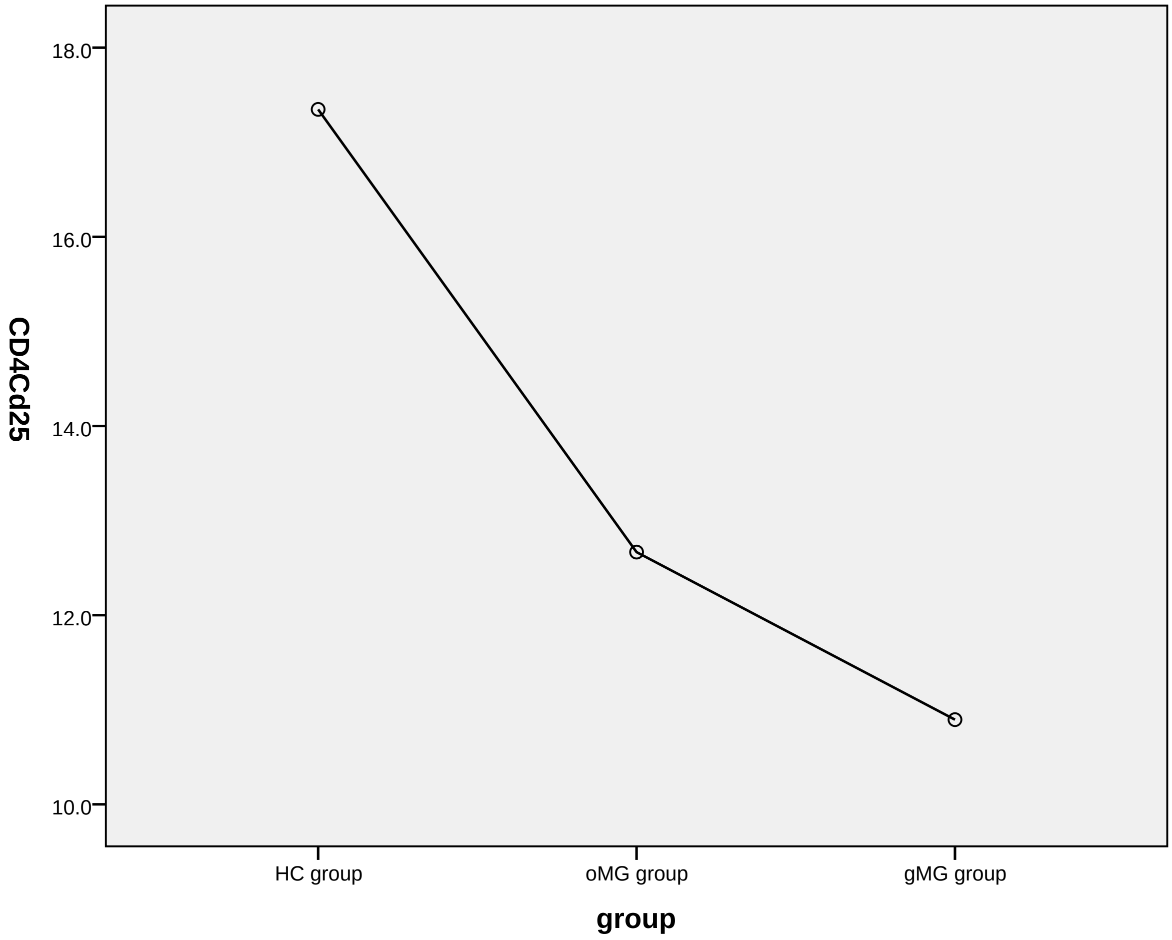


```
EXAMINE VARIABLES=CD25Foxp3 BY group
  /PLOT BOXPLOT NPPLOT
  /COMPARE GROUPS
  /STATISTICS NONE
  /CINTERVAL 95
  /MISSING LISTWISE
  /NOTOTAL.
```

	16-1-2022 215959
	1
	<none>
	<none>
	<none>
N	58
	EXAMINE VARIABLES=CD25Foxp3 BY group /PLOT BOXPLOT NPPLOT /COMPARE GROUPS /STATISTICS NONE /CINTERVAL 95 /MISSING LISTWISE /NOTOTAL.
	00 00:00:00.719
	00 00:00:00.719

[1]

group

	group	N		N		N	
CD25Foxp3	HC group	19	100.0%	0	.0%	19	100.0%
	oMG group	18	100.0%	0	.0%	18	100.0%
	gMG group	21	100.0%	0	.0%	21	100.0%

		Kolmogorov-Smirnov[a]			Shapiro-Wilk		
	group		df	Sig.		df	Sig.
CD25Foxp3	HC group	.156	19	.200*	.947	19	.354
	oMG group	.139	18	.200*	.954	18	.496
	gMG group	.115	21	.200*	.952	21	.375

a. Lilliefors
* .

CD25Foxp3

Q - Q

CD25Foxp3 Q-Q

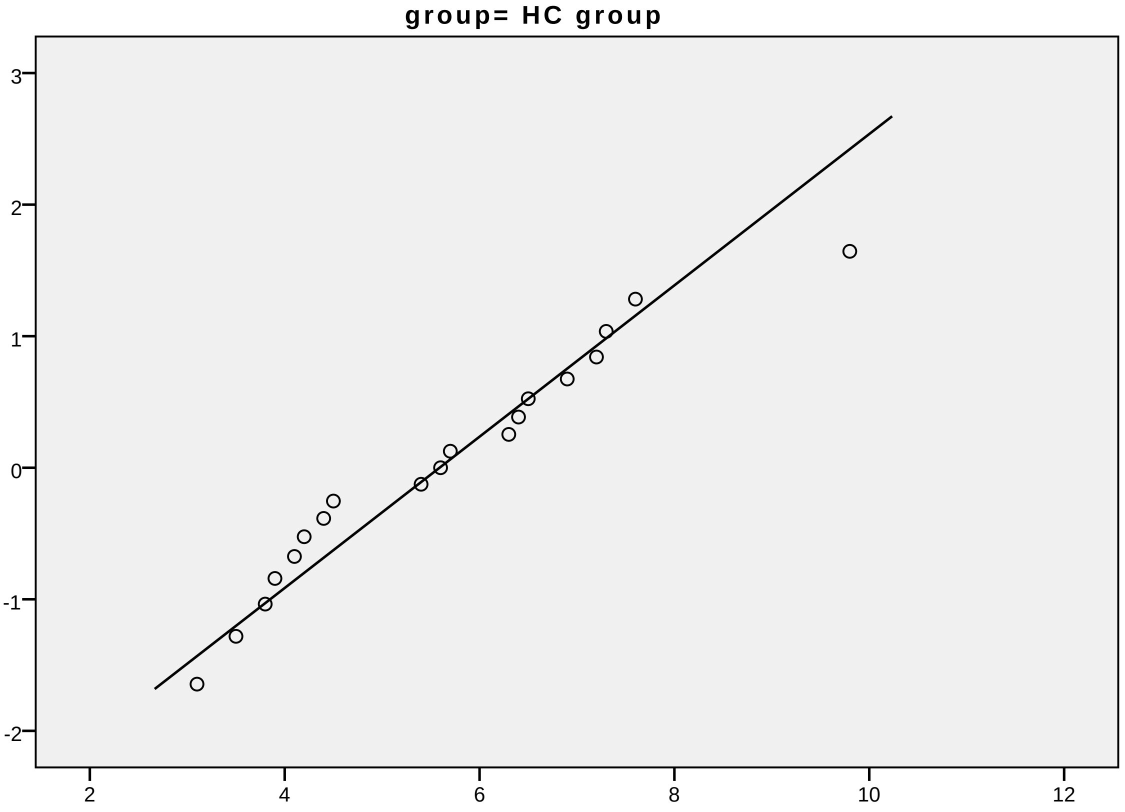

CD25Foxp3 Q-Q

group= oMG group

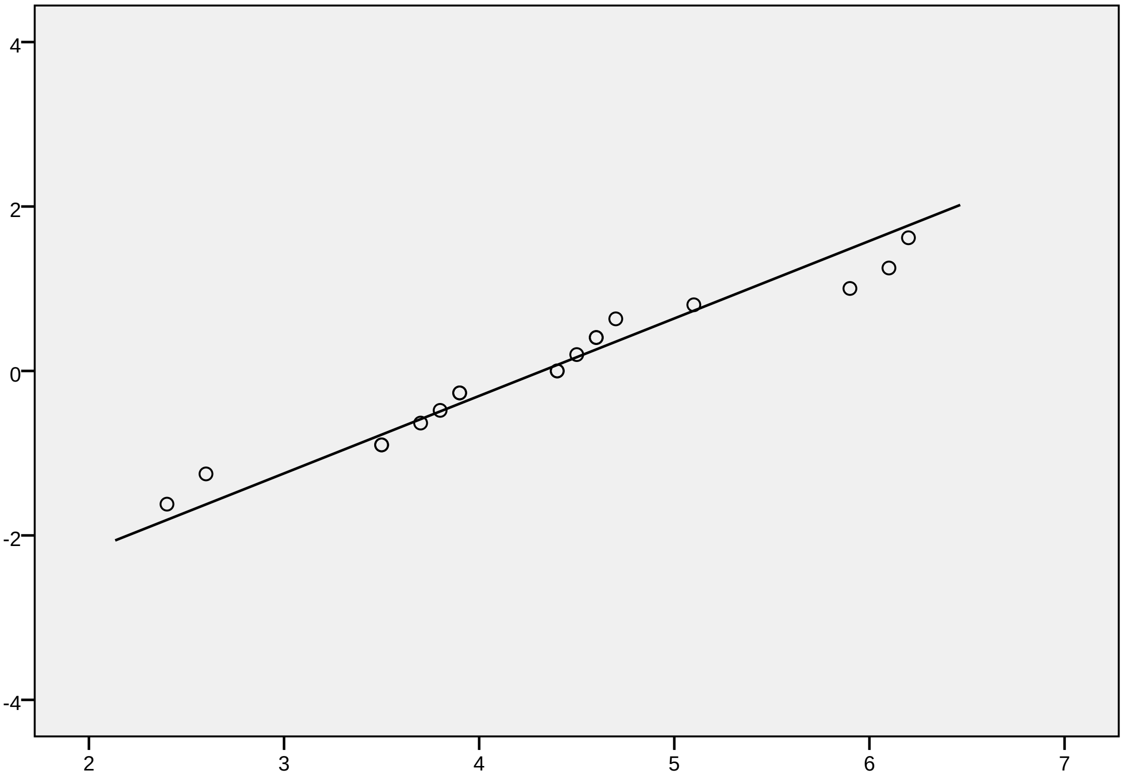

CD25Foxp3 Q-Q

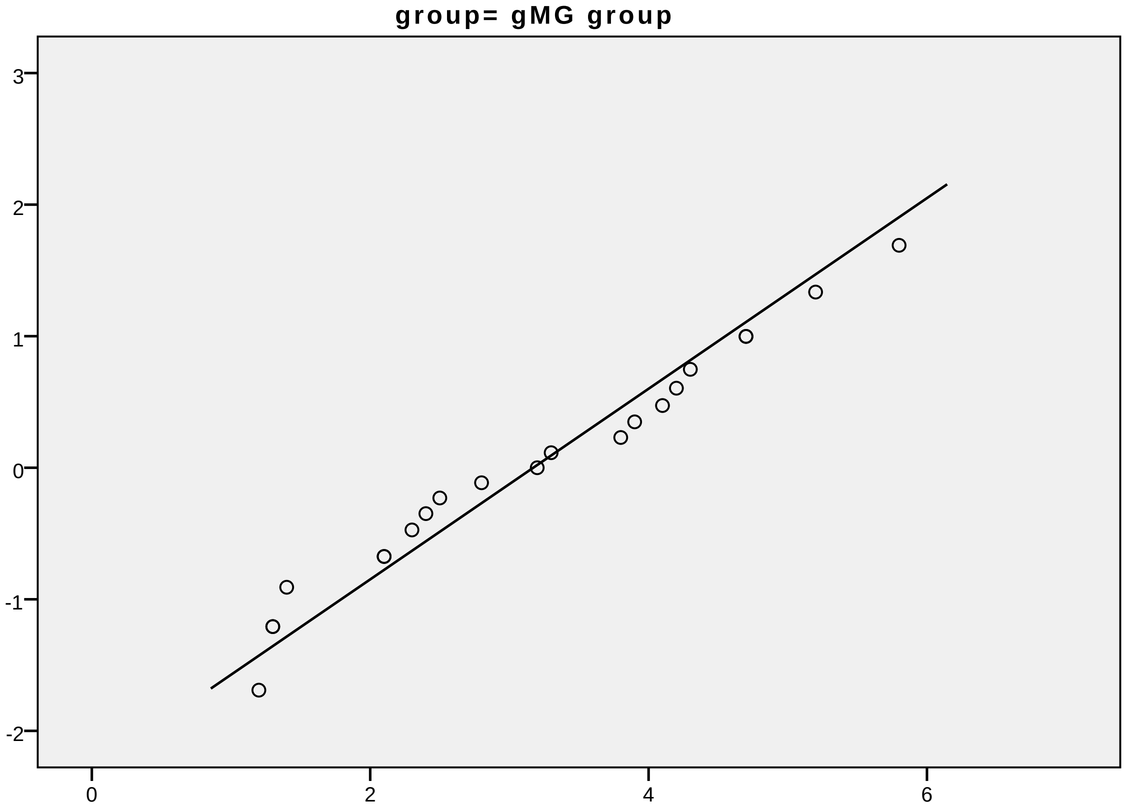

Q - Q

C D 2 5 F o x p 3 Q - Q

group= HC group

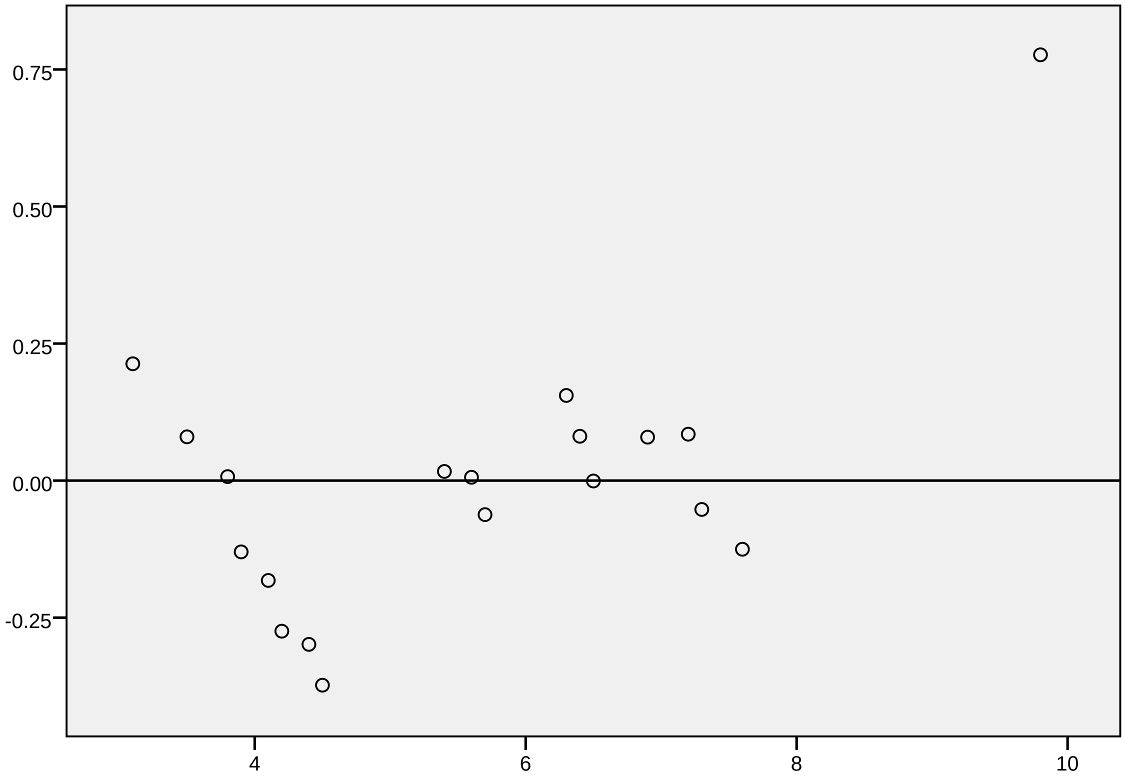

CD25Foxp3 Q-Q

group= oMG group

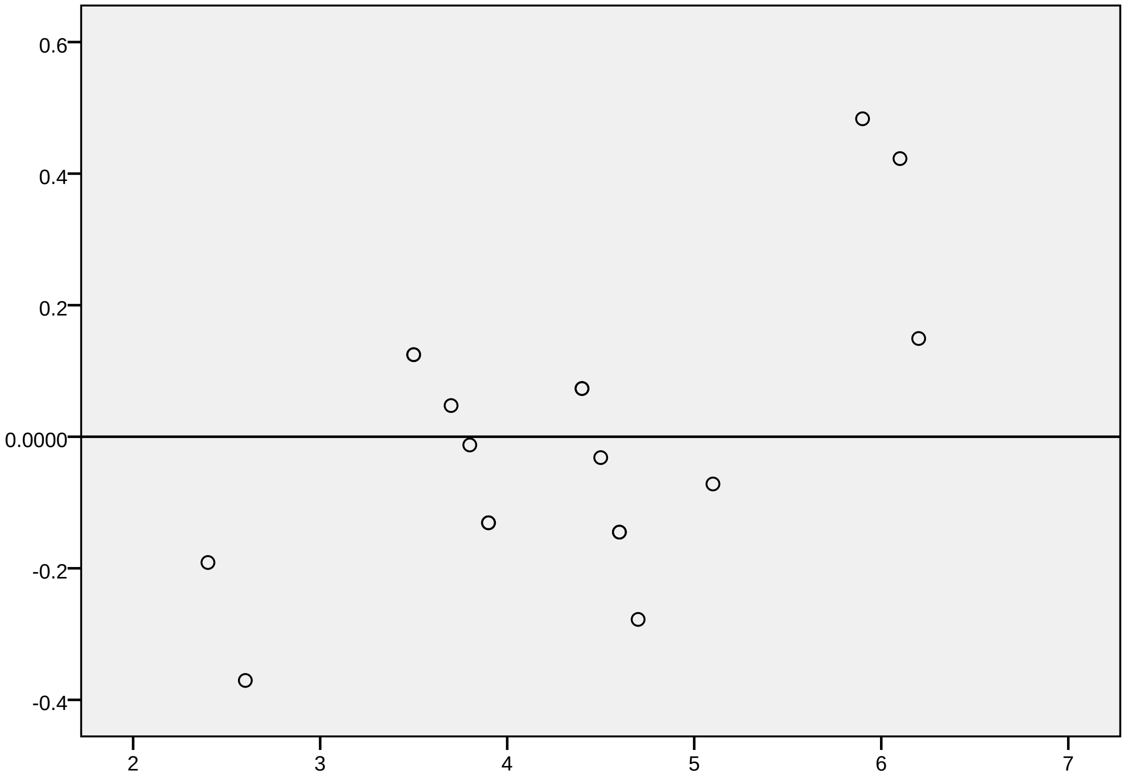

C D 2 5 F o x p 3 Q - Q

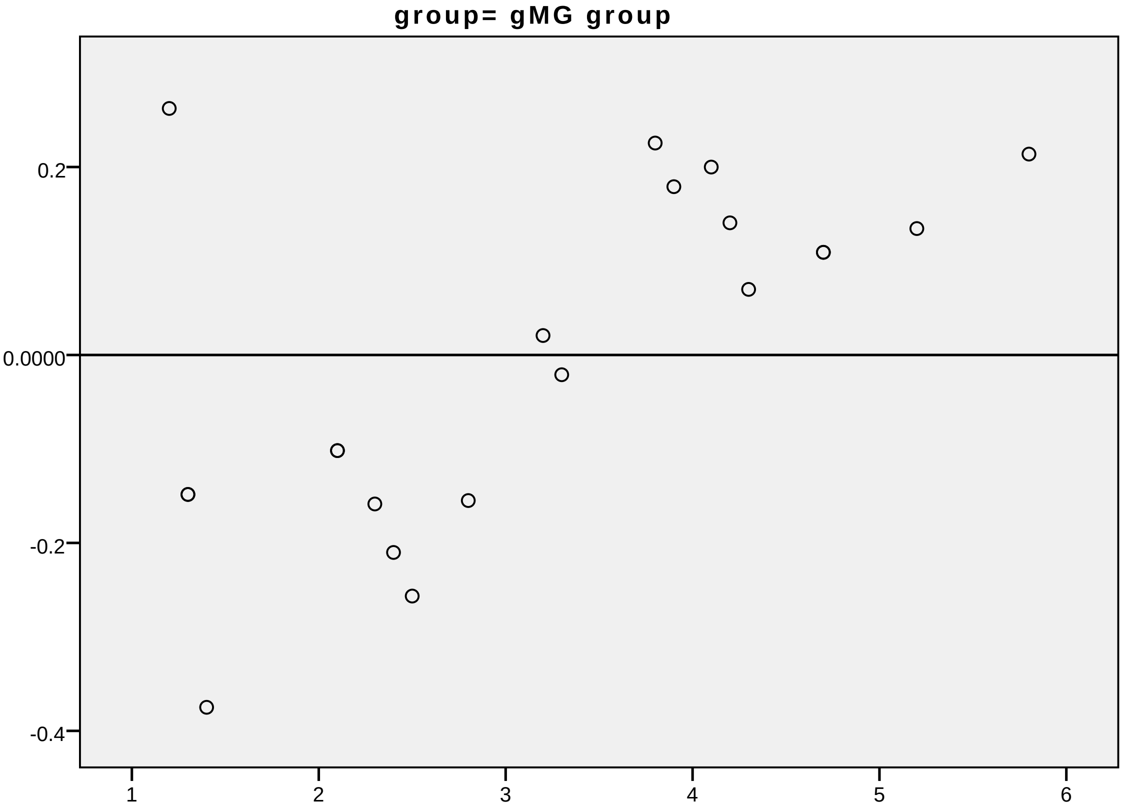

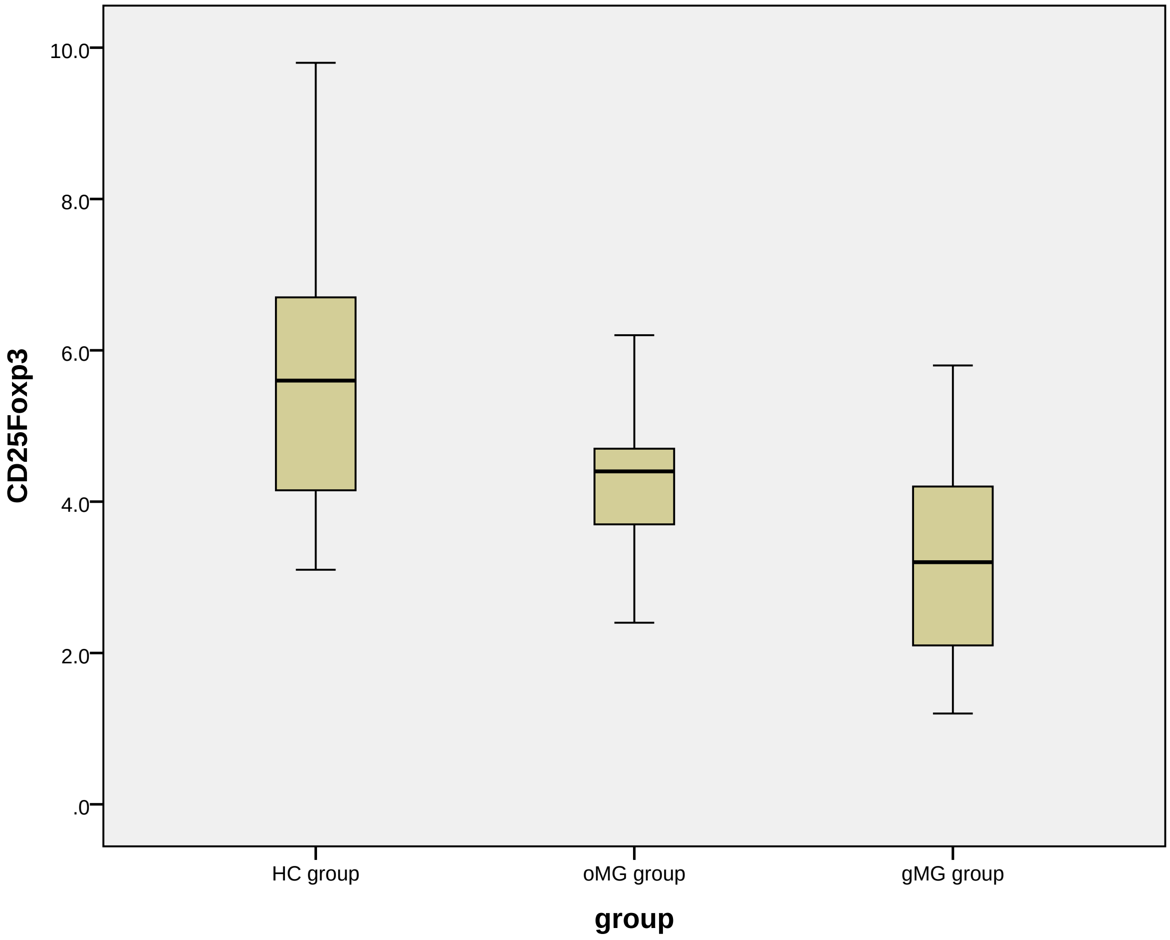


```
ONEWAY CD25Foxp3 BY group
  /STATISTICS DESCRIPTIVES HOMOGENEITY WELCH
  /PLOT MEANS
  /MISSING ANALYSIS
  /POSTHOC=TUKEY GH ALPHA(0.05).
```

	16-1-2022 220008
	1
	<none>
	<none>
	<none>
N	58
	ONEWAY CD25Foxp3 BY group /STATISTICS DESCRIPTIVES HOMOGENEITY WELCH /PLOT MEANS /MISSING ANALYSIS /POSTHOC=TUKEY GH ALPHA (0.05).
	00 00:00:00.125
	00 00:00:00.123

[1]

CD25Foxp3

	N				9 5 %	
HC group	19	5.589	1.7387	.3989	4.751	6.428
oMG group	18	4.322	1.0614	.2502	3.794	4.850
gMG group	21	3.171	1.3803	.3012	2.543	3.800
	58	4.321	1.7270	.2268	3.867	4.775

CD25Foxp3

HC group	3.1	9.8
oMG group	2.4	6.2
gMG group	1.2	5.8
	1.2	9.8

CD25Foxp3

Levene	df1	df2	
2.813	2	55	.069

ANOVA

CD25Foxp3

		df		F	
	58.323	2	29.162	14.363	.000
	111.672	55	2.030		
	169.995	57			

CD25Foxp3

	[a]	df1	df2	
Welch	11.790	2	35.637	.000

a . F

: C D 2 5 F o x p 3

	(I) group	(J) group	(I - J)		
Tukey HSD	HC group	oMG group	1.2673*	.4687	.024
		gMG group	2.4180*	.4512	.000
	oMG group	HC group	-1.2673*	.4687	.024
		gMG group	1.1508*	.4577	.039
	gMG group	HC group	-2.4180*	.4512	.000
		oMG group	-1.1508*	.4577	.039
Games-Howell	HC group	oMG group	1.2673*	.4709	.030
		gMG group	2.4180*	.4998	.000
	oMG group	HC group	-1.2673*	.4709	.030
		gMG group	1.1508*	.3915	.015
	gMG group	HC group	-2.4180*	.4998	.000
		oMG group	-1.1508*	.3915	.015

: C D 2 5 F o x p 3

	(I) group	(J) group	9 5 %	
Tukey HSD	HC group	oMG group	.138	2.396
		gMG group	1.331	3.505
	oMG group	HC group	-2.396	-.138
		gMG group	.048	2.253
	gMG group	HC group	-3.505	-1.331
		oMG group	-2.253	-.048
Games-Howell	HC group	oMG group	.107	2.428
		gMG group	1.194	3.642
	oMG group	HC group	-2.428	-.107
		gMG group	.194	2.107
	gMG group	HC group	-3.642	-1.194
		oMG group	-2.107	-.194

* . 0 . 0 5

CD25Foxp3

	group	N	a l p h a = 0 . 0 5		
			1	2	3
Tukey HSD[a,b]	gMG group	21	3.171		
	oMG group	18		4.322	
	HC group	19			5.589
			1.000	1.000	1.000

a . = 1 9 . 2 5 5
b . I

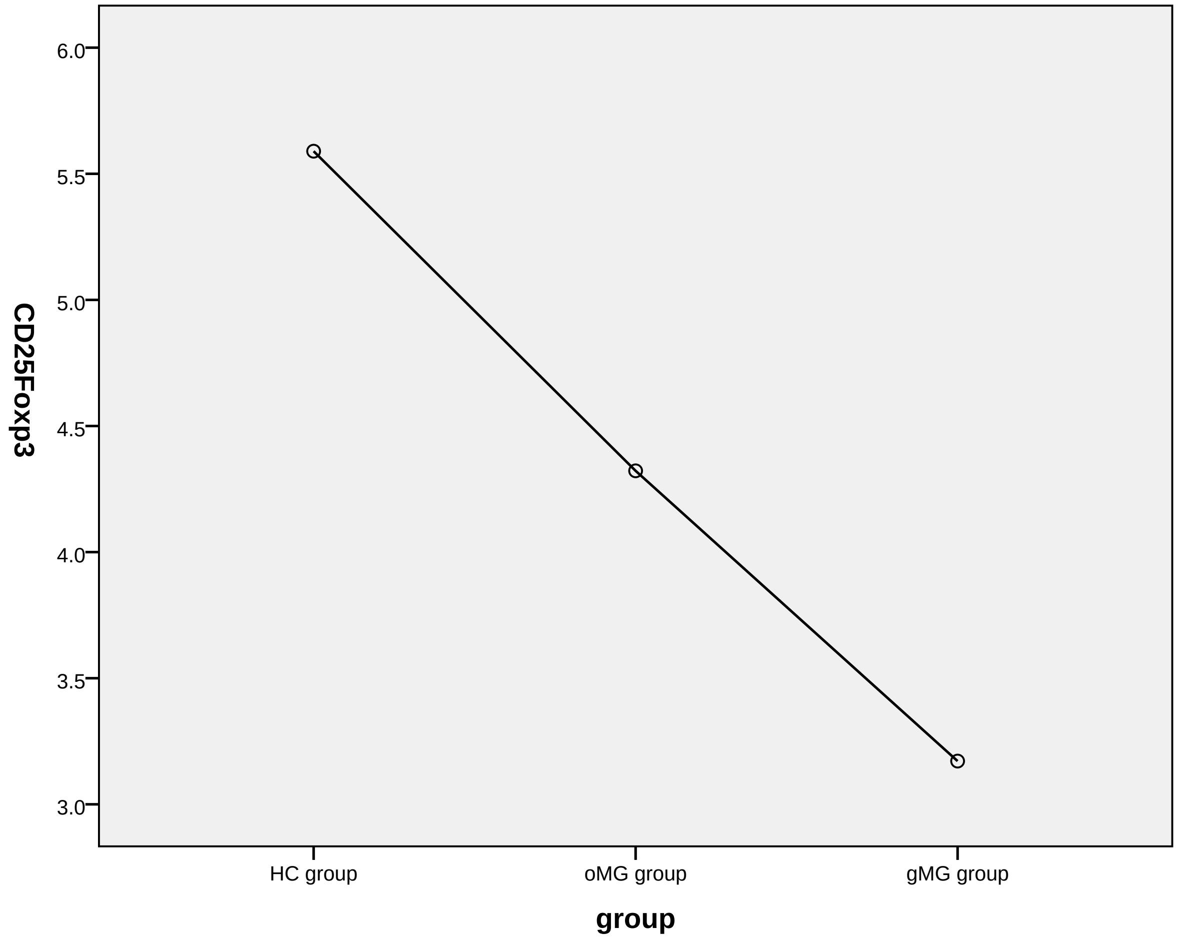


```
EXAMINE VARIABLES=rTreg BY group
  /PLOT BOXPLOT NPPLOT
  /COMPARE GROUPS
  /STATISTICS NONE
  /CINTERVAL 95
  /MISSING LISTWISE
  /NOTOTAL.
```

	16-1-2022 220019
	1
	<none>
	<none>
	<none>
N	58
	EXAMINE VARIABLES=rTreg BY group /PLOT BOXPLOT NPPLOT /COMPARE GROUPS /STATISTICS NONE /CINTERVAL 95 /MISSING LISTWISE /NOTOTAL.
	00 00:00:00.734
	00 00:00:00.711

[1]

group

	group	N		N		N	
rTreg	HC group	19	100.0%	0	.0%	19	100.0%
	oMG group	18	100.0%	0	.0%	18	100.0%
	gMG group	21	100.0%	0	.0%	21	100.0%

		Kolmogorov-Smirnov [a]			Shapiro-Wilk		
	group		df	Sig.		df	Sig.
rTreg	HC group	.141	19	.200*	.933	19	.198
	oMG group	.170	18	.183	.937	18	.261
	gMG group	.200	21	.029	.930	21	.137

a . Lilliefors
* .

rTreg

Q - Q

rTreg Q-Q

group= HC group

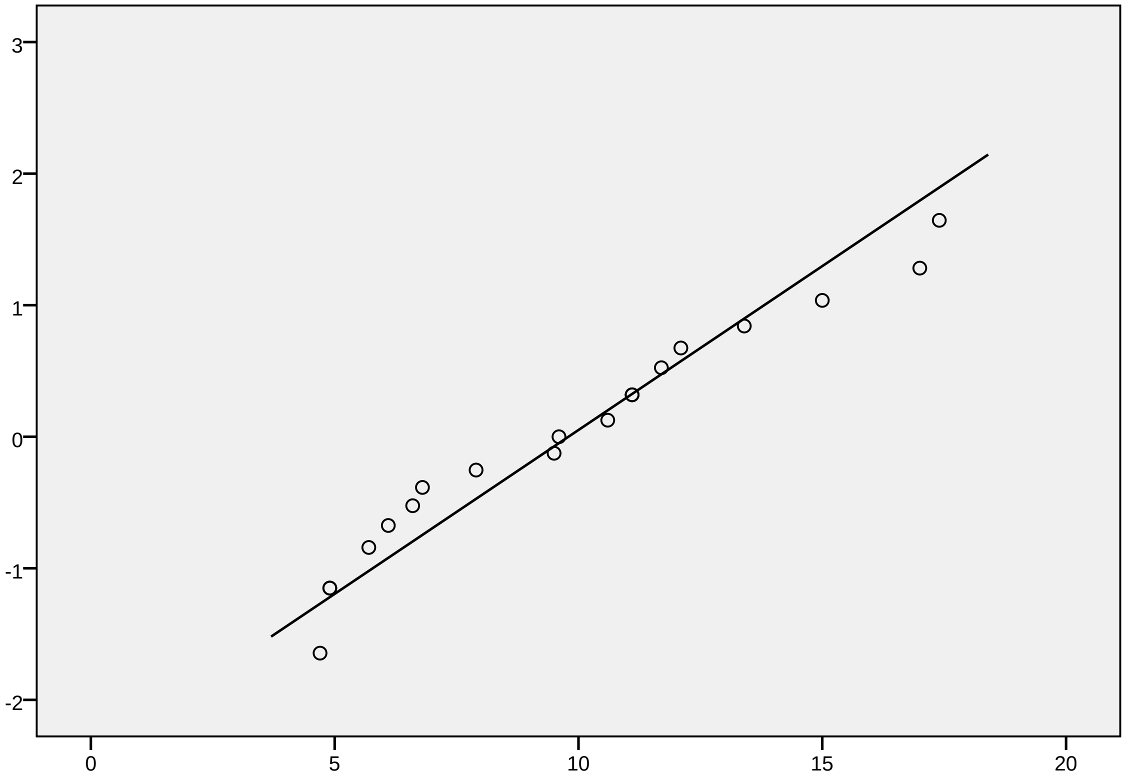

rTreg Q-Q

group= oMG group

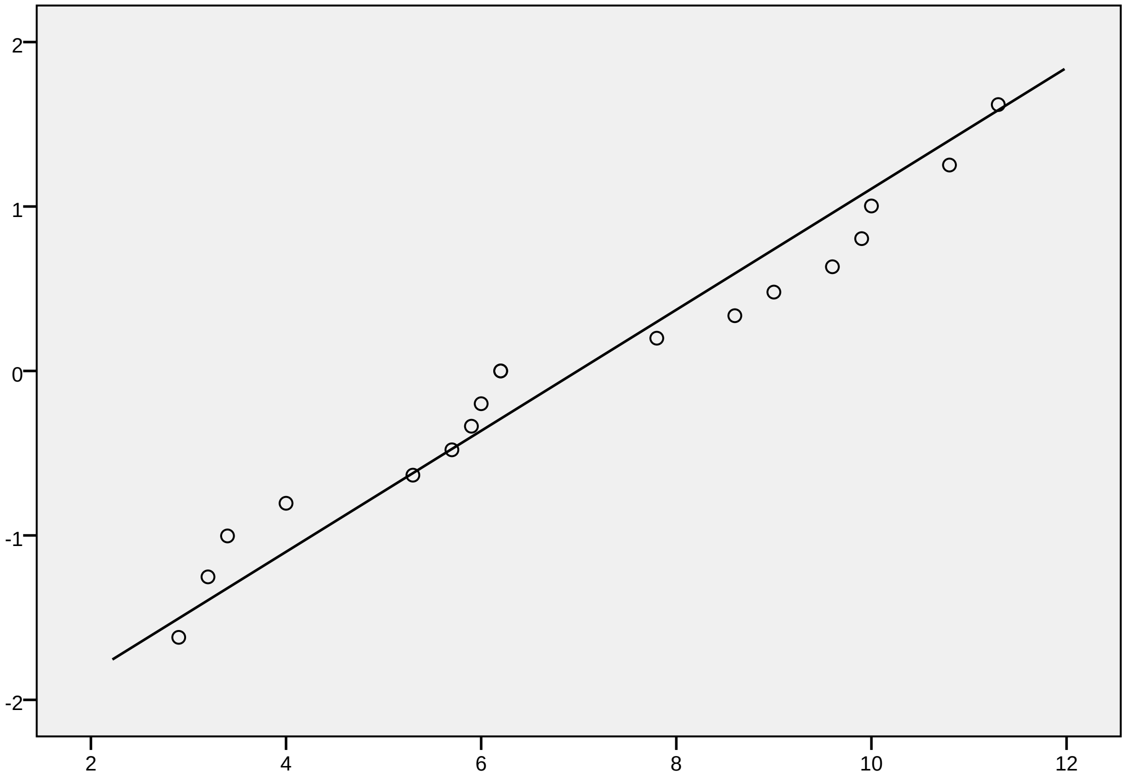

rTreg Q-Q

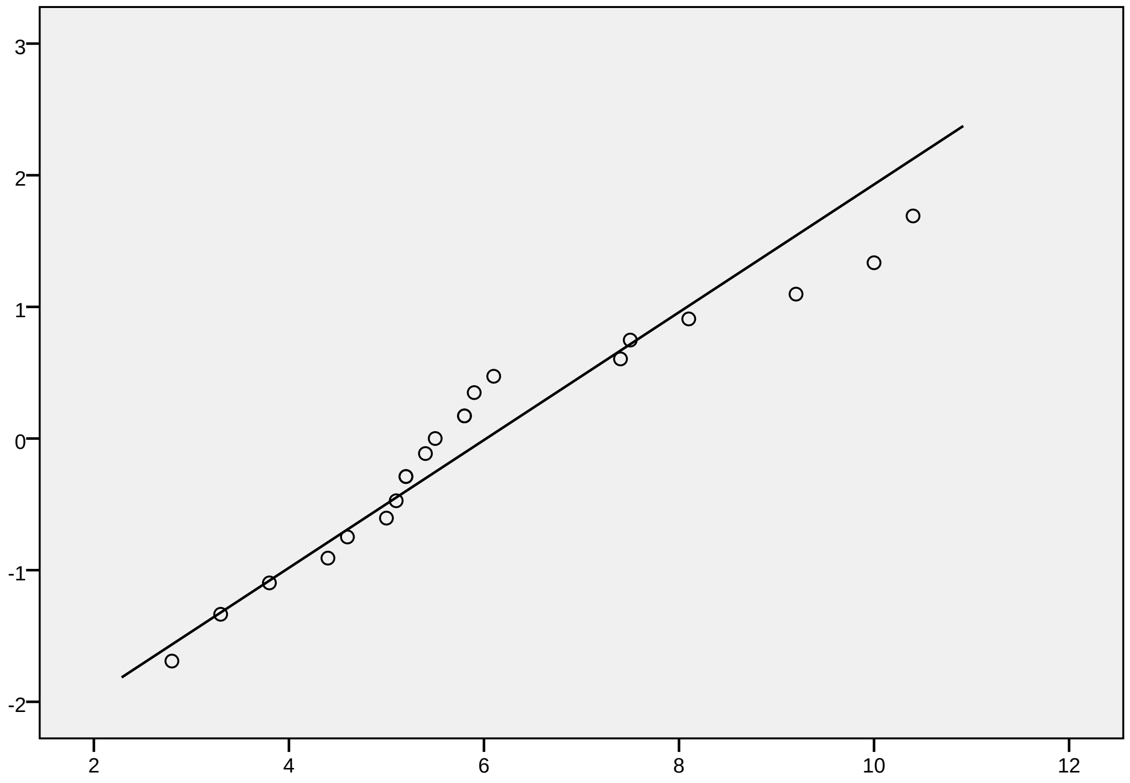

Q - Q

r T r e g Q - Q

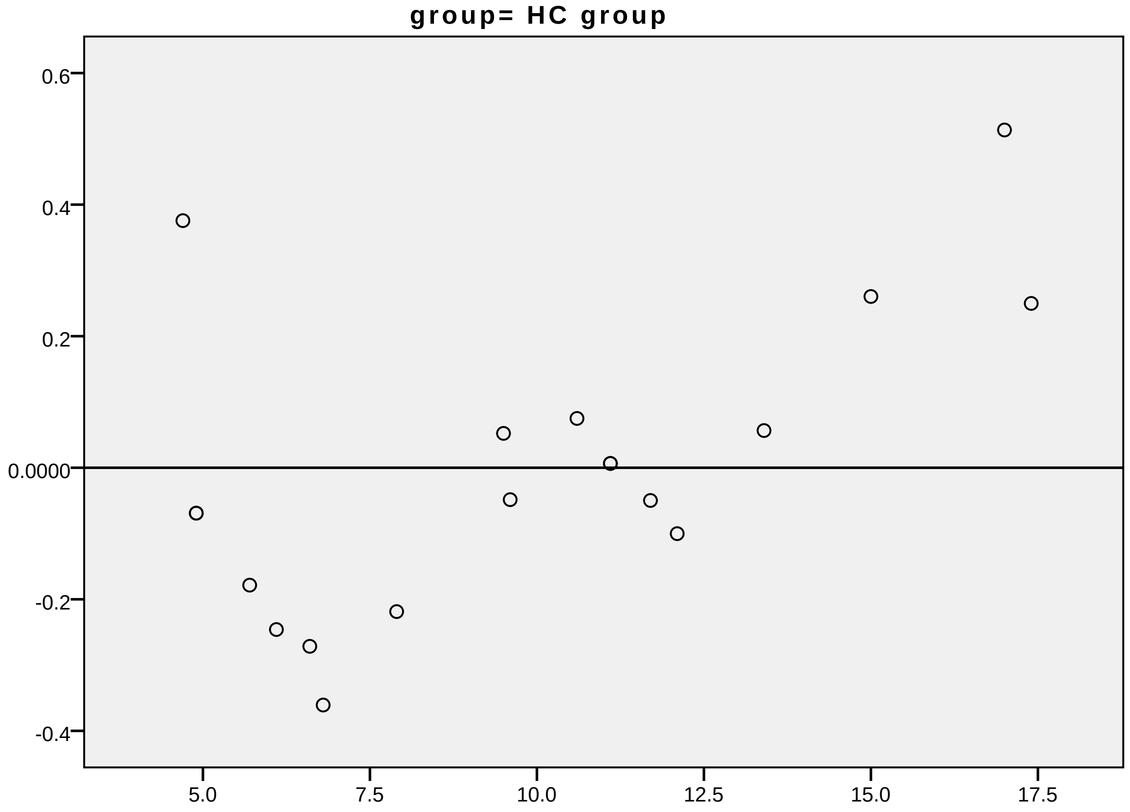

rTreg Q-Q

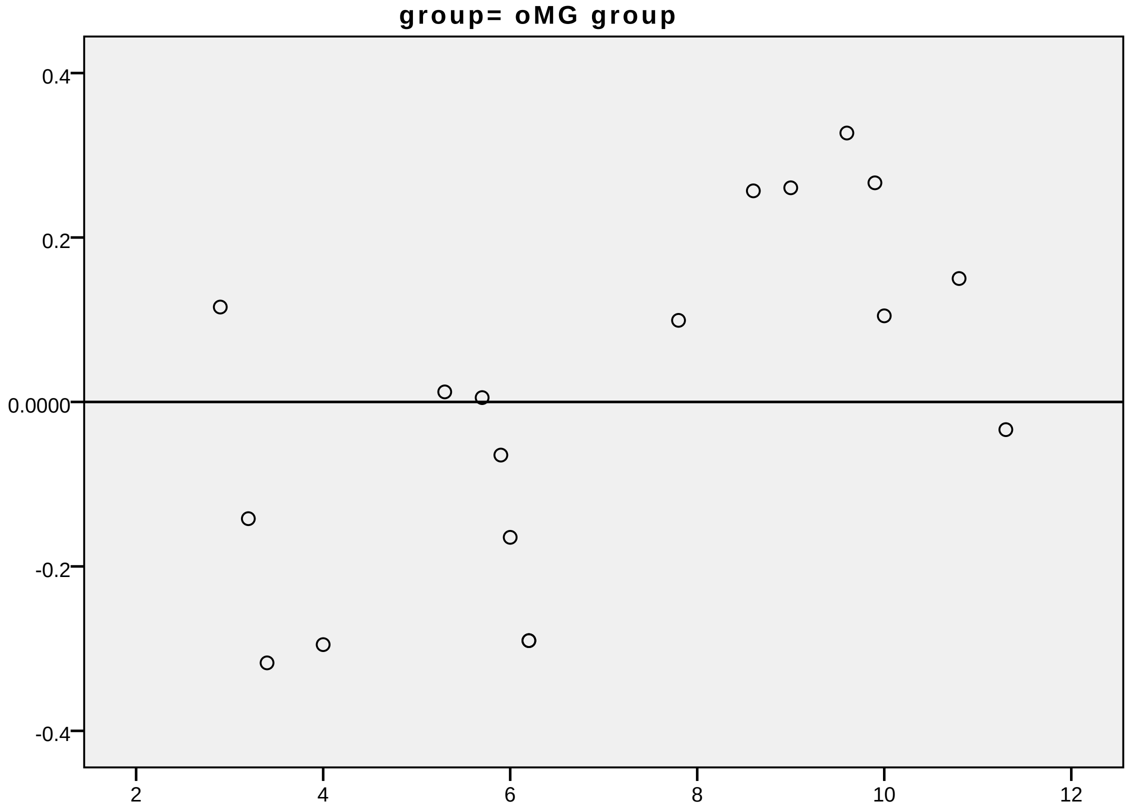

rTreg Q-Q

group= gMG group

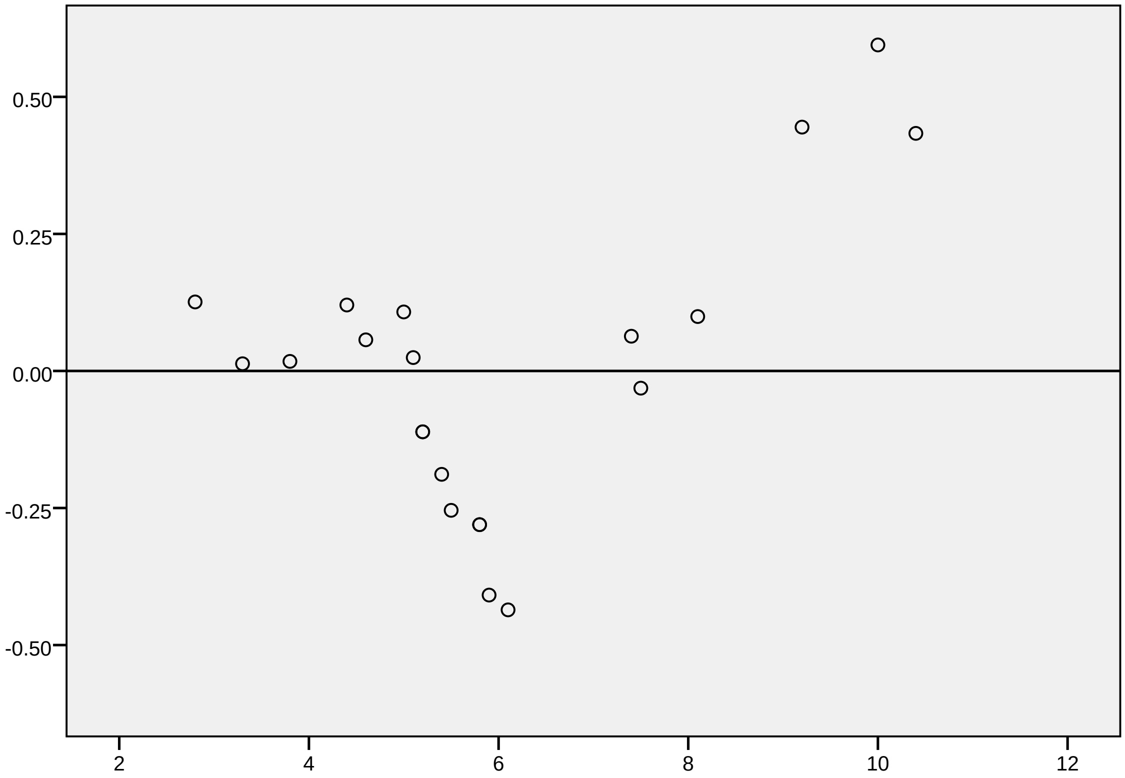

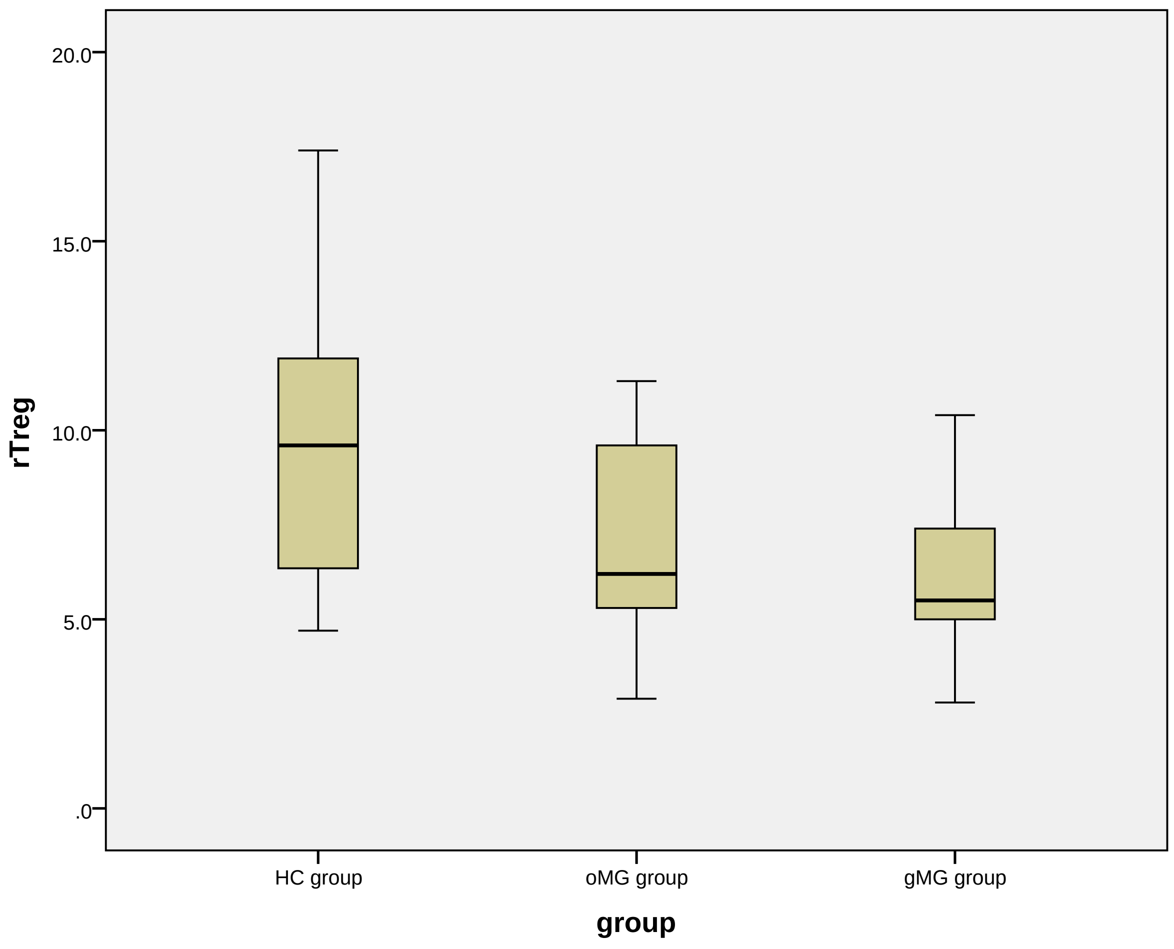


```
EXAMINE VARIABLES=nsTreg BY group
  /PLOT BOXPLOT NPPLOT
  /COMPARE GROUPS
  /STATISTICS NONE
  /CINTERVAL 95
  /MISSING LISTWISE
  /NOTOTAL.
```

	16-1-2022 220036
	1
	<none>
	<none>
	<none>
N	58
	EXAMINE VARIABLES=nsTreg BY group /PLOT BOXPLOT NPPLOT /COMPARE GROUPS /STATISTICS NONE /CINTERVAL 95 /MISSING LISTWISE /NOTOTAL.
	00 00:00:00.735
	00 00:00:00.708

[1]

group

	group	N		N		N	
nsTreg	HC group	19	100.0%	0	.0%	19	100.0%
	oMG group	18	100.0%	0	.0%	18	100.0%
	gMG group	21	100.0%	0	.0%	21	100.0%

		Kolmogorov-Smirnov[a]			Shapiro-Wilk		
	group		df	Sig.		df	Sig.
nsTreg	HC group	.118	19	.200*	.975	19	.876
	oMG group	.175	18	.153	.974	18	.867
	gMG group	.110	21	.200*	.953	21	.383

a. Lilliefors

*.

nsTreg

Q - Q

nsTreg Q-Q

group= HC group

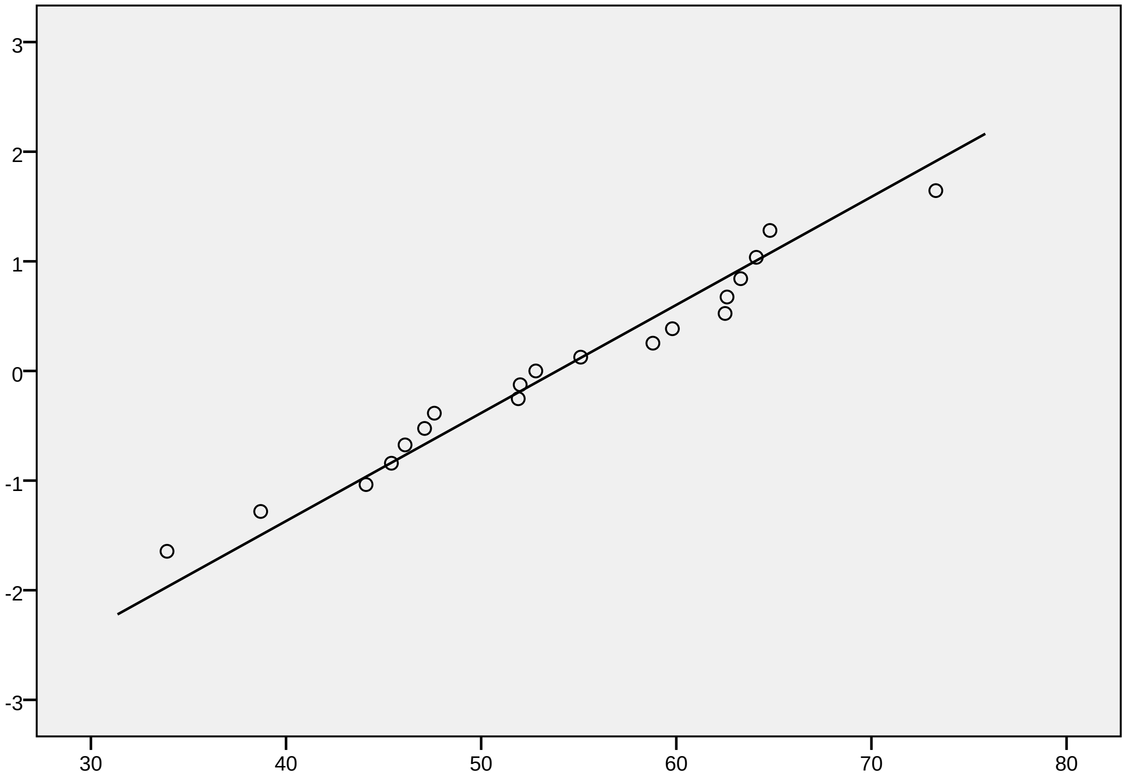

nsTreg Q-Q

group= oMG group

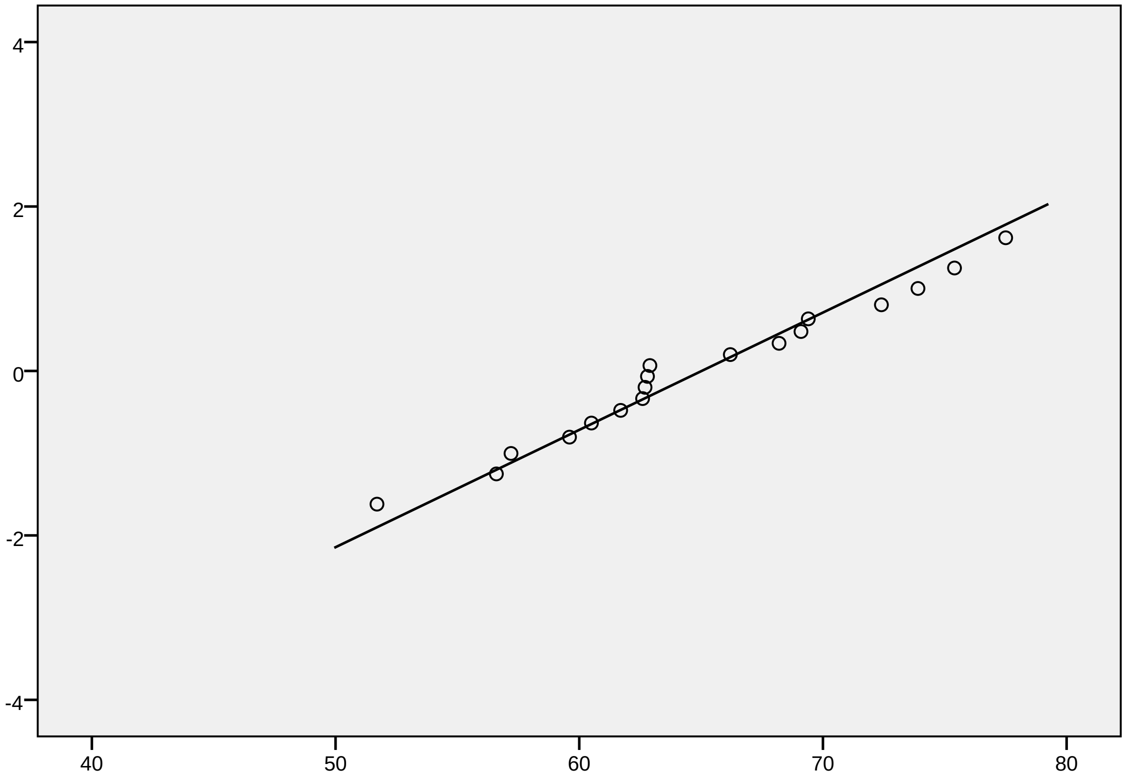

nsTreg Q-Q

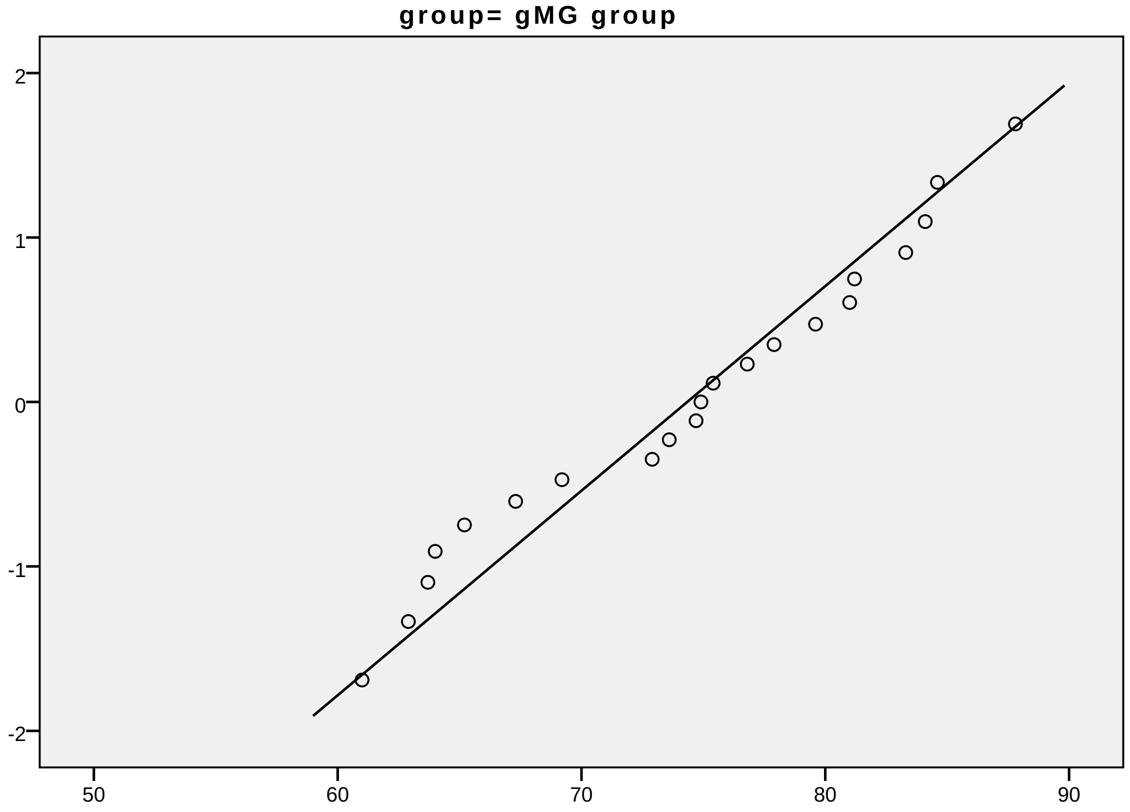

Q - Q

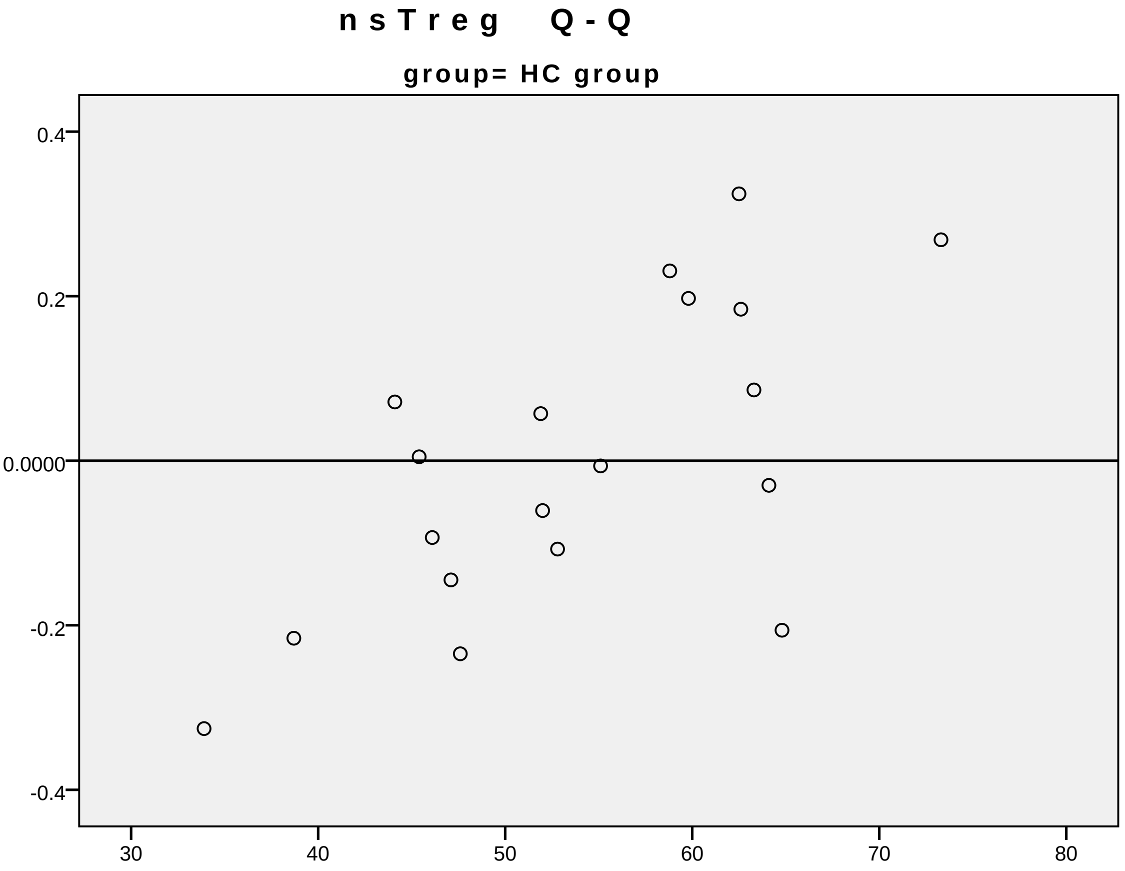
n s T r e g Q - Q
group= HC group
0.4
0.2
0.0000
-0.2
-0.4
30
40
50
60
70
80

n s T r e g Q - Q

group= oMG group

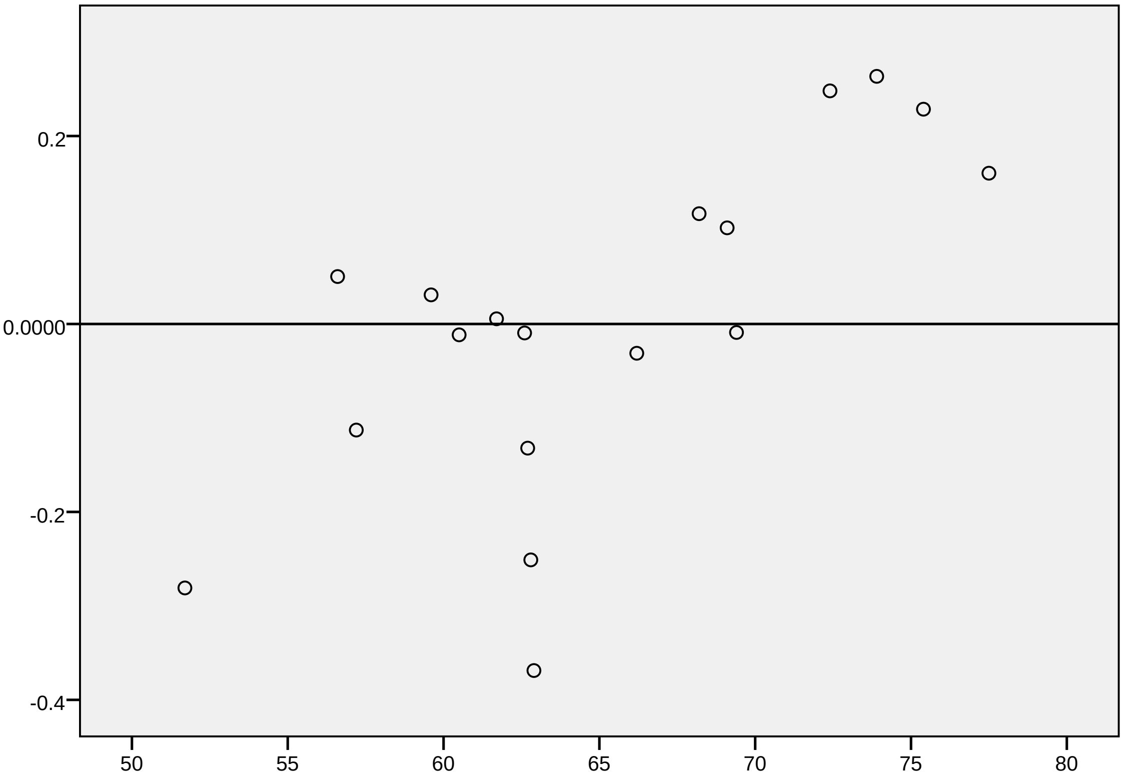

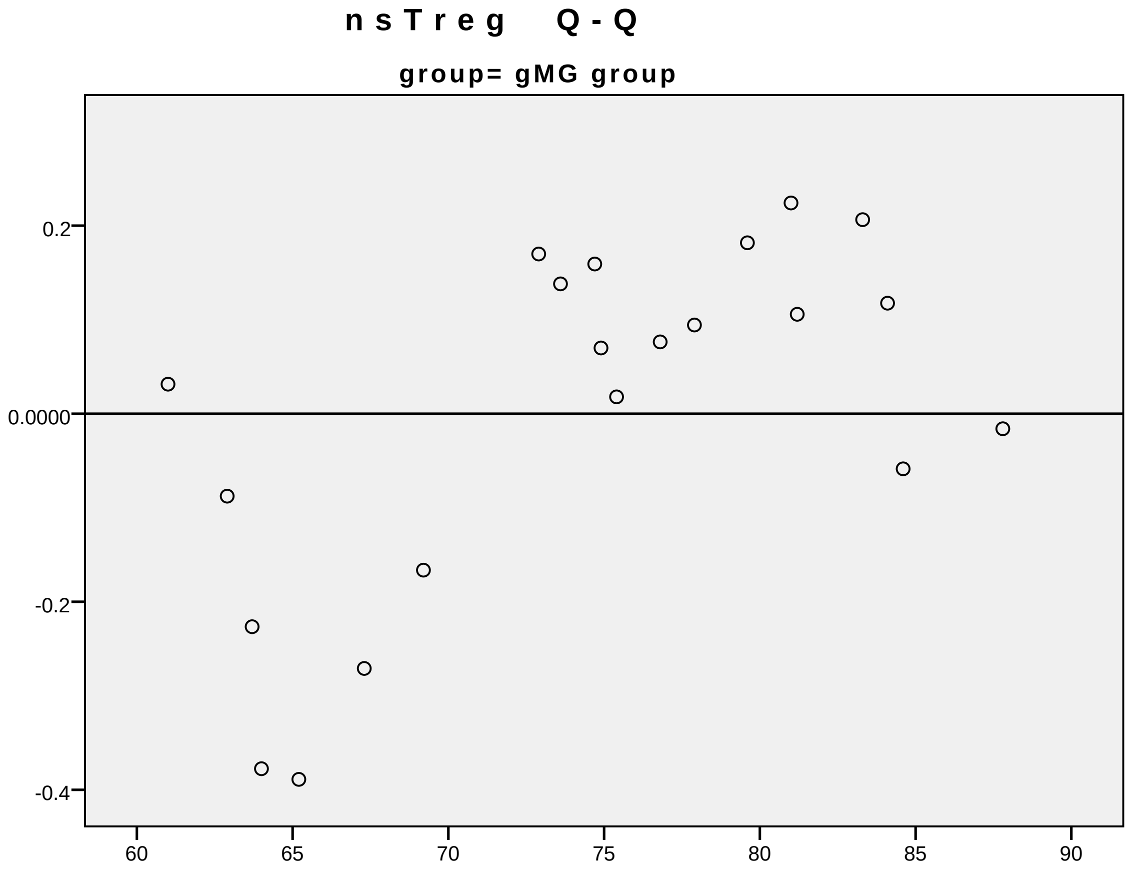
n s T r e g Q - Q
group= gMG group
0.2
0.0000
-0.2
-0.4
60
65
70
75
80
85
90

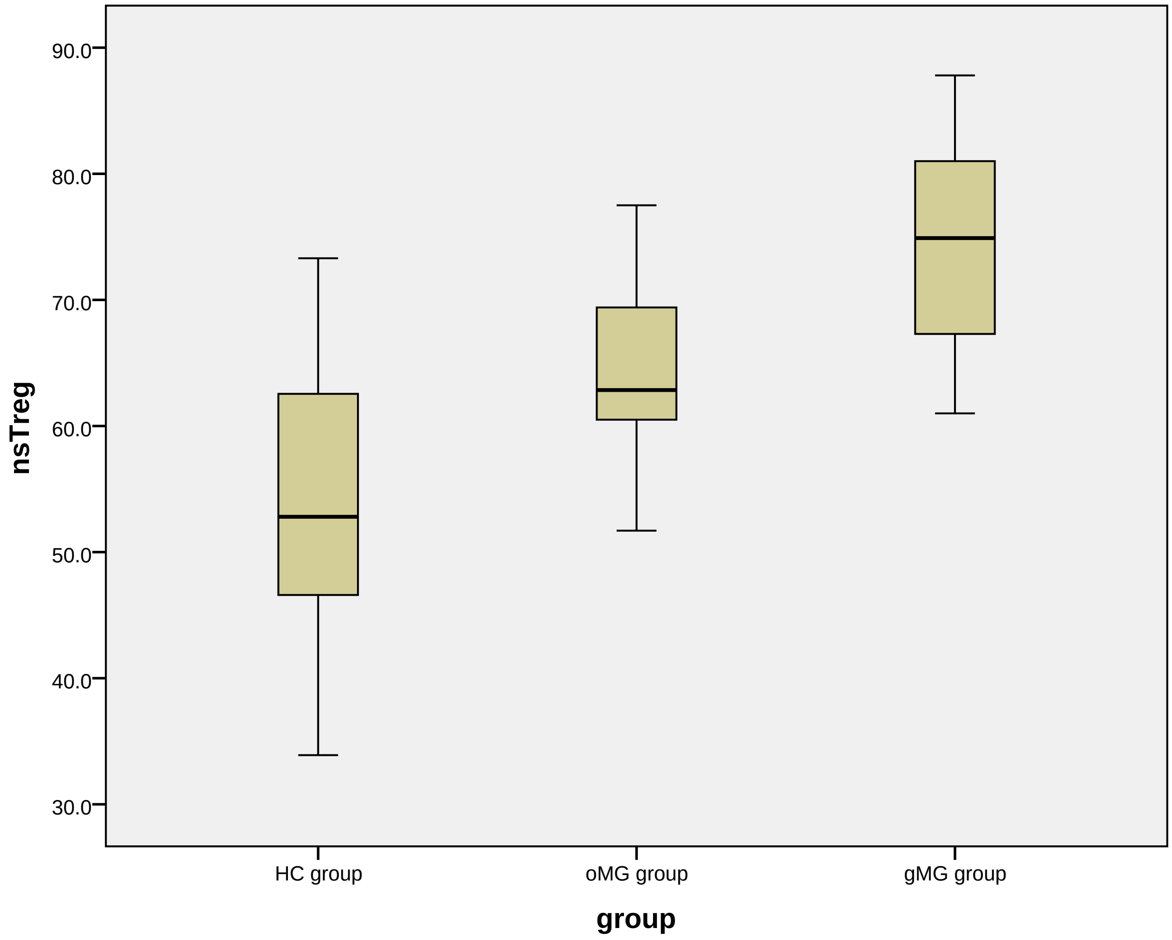


```
ONEWAY rTreg BY group
  /STATISTICS DESCRIPTIVES HOMOGENEITY WELCH
  /PLOT MEANS
  /MISSING ANALYSIS
  /POSTHOC=TUKEY GH ALPHA(0.05).
```

	16-1-2022 220103
	1
	<none>
	<none>
	<none>
N	58
	ONEWAY rTreg BY group /STATISTICS DESCRIPTIVES HOMOGENEITY WELCH /PLOT MEANS /MISSING ANALYSIS /POSTHOC=TUKEY GH ALPHA (0.05).
	00 00:00:00.109
	00 00:00:00.124

[1]

rTreg

	N				9 5 %	
HC group	19	9.795	4.0141	.9209	7.860	11.729
oMG group	18	6.989	2.7179	.6406	5.637	8.340
gMG group	21	6.024	2.0603	.4496	5.086	6.962
	58	7.559	3.3790	.4437	6.670	8.447

rTreg

HC group	4.7	17.4
oMG group	2.9	11.3
gMG group	2.8	10.4
	2.8	17.4

rTreg

Levene	df1	df2	
5.603	2	55	.006

ANOVA

rTreg

		df		F	
	150.315	2	75.158	8.259	.001
	500.505	55	9.100		
	650.821	57			

rTreg

	[a]	df1	df2	
Welch	6.686	2	33.195	.004

a . F

: r T r e g

	(I) group	(J) group	(I - J)		
Tukey HSD	HC group	oMG group	2.8058*	.9922	.018
		gMG group	3.7709*	.9551	.001
	oMG group	HC group	-2.8058*	.9922	.018
		gMG group	.9651	.9690	.583
	gMG group	HC group	-3.7709*	.9551	.001
		oMG group	-.9651	.9690	.583
Games-Howell	HC group	oMG group	2.8058*	1.1218	.045
		gMG group	3.7709*	1.0248	.003
	oMG group	HC group	-2.8058*	1.1218	.045
		gMG group	.9651	.7826	.443
	gMG group	HC group	-3.7709*	1.0248	.003
		oMG group	-.9651	.7826	.443

: r T r e g

	(I) group	(J) group	9 5 %	
Tukey HSD	HC group	oMG group	.416	5.196
		gMG group	1.470	6.072
	oMG group	HC group	-5.196	-.416
		gMG group	-1.369	3.299
	gMG group	HC group	-6.072	-1.470
		oMG group	-3.299	1.369
Games-Howell	HC group	oMG group	.048	5.564
		gMG group	1.226	6.316
	oMG group	HC group	-5.564	-.048
		gMG group	-.960	2.890
	gMG group	HC group	-6.316	-1.226
		oMG group	-2.890	.960

* . 0 . 0 5

rTreg

	group	N	alpha = 0.05	
			1	2
Tukey HSD[a,b]	gMG group	21	6.024	
	oMG group	18	6.989	
	HC group	19		9.795
			.585	1.000

a . = 1 9 . 2 5 5
b . I

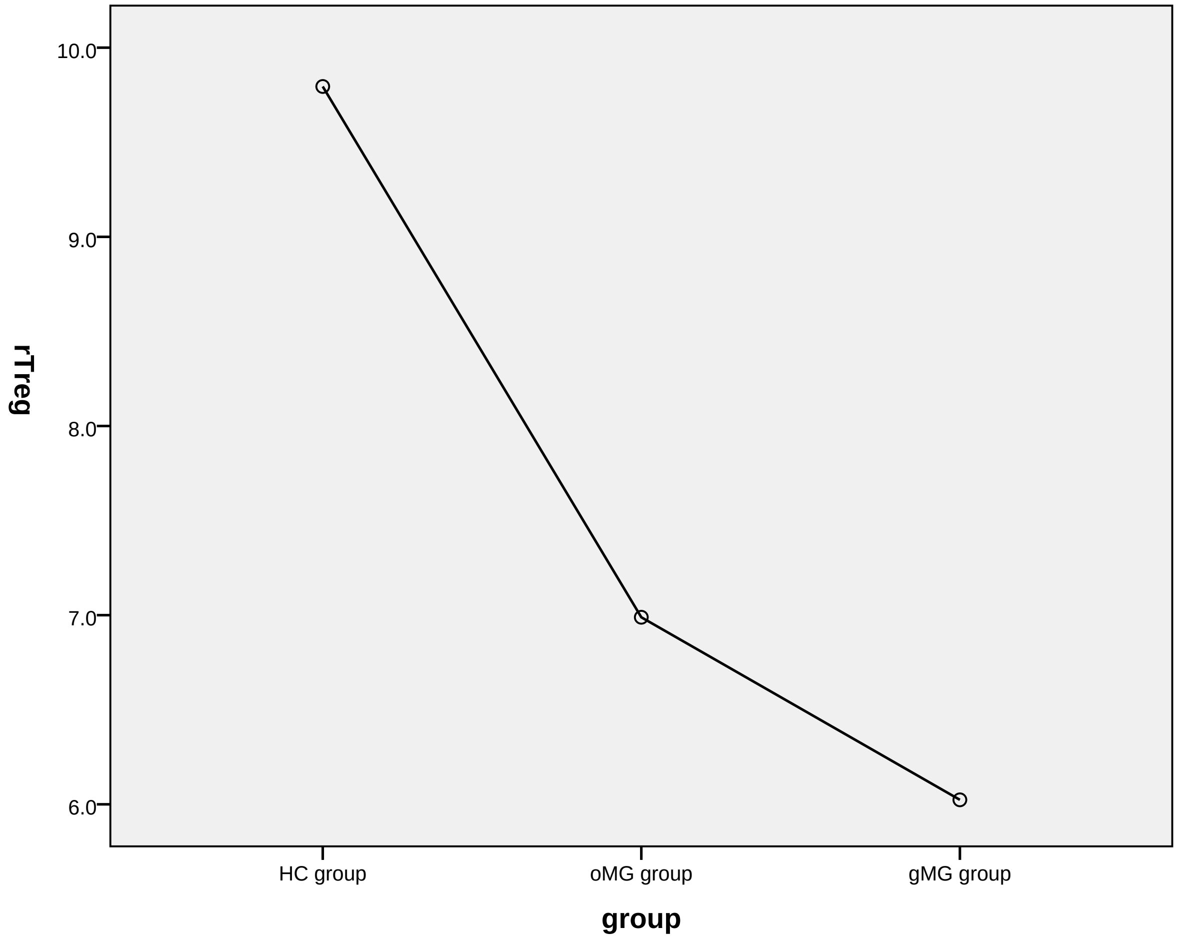


```
ONEWAY nsTreg BY group
  /STATISTICS DESCRIPTIVES HOMOGENEITY WELCH
  /PLOT MEANS
  /MISSING ANALYSIS
  /POSTHOC=TUKEY GH ALPHA(0.05).
```

	16-1-2022 220141
	1
	<none>
	<none>
	<none>
N	58
	ONEWAY nsTreg BY group<br>/STATISTICS DESCRIPTIVES HOMOGENEITY WELCH<br>/PLOT MEANS<br>/MISSING ANALYSIS<br>/POSTHOC=TUKEY GH ALPHA (0.05).
	00 00:00:00.125
	00 00:00:00.122

[1]

nsTreg

	N				9 5 %	
HC group	19	53.889	10.1445	2.3273	49.000	58.779
oMG group	18	65.022	7.0089	1.6520	61.537	68.508
gMG group	21	74.338	8.0388	1.7542	70.679	77.997
	58	64.748	11.9597	1.5704	61.604	67.893

nsTreg

HC group	33.9	73.3
oMG group	51.7	77.5
gMG group	61.0	87.8
	33.9	87.8

nsTreg

Levene	df1	df2	
1.556	2	55	.220

ANOVA

nsTreg

		df		F	
	4172.966	2	2086.483	28.834	.000
	3979.959	55	72.363		
	8152.925	57			

nsTreg

	[a]	df1	df2	
Welch	24.547	2	35.893	.000

a . F

: n s T r e g

	(I) group	(J) group	(I - J)		
Tukey HSD	HC group	oMG group	-11.1327*	2.7980	.001
		gMG group	-20.4486*	2.6934	.000
	oMG group	HC group	11.1327*	2.7980	.001
		gMG group	-9.3159*	2.7324	.003
	gMG group	HC group	20.4486*	2.6934	.000
		oMG group	9.3159*	2.7324	.003
Games-Howell	HC group	oMG group	-11.1327*	2.8540	.001
		gMG group	-20.4486*	2.9144	.000
	oMG group	HC group	11.1327*	2.8540	.001
		gMG group	-9.3159*	2.4096	.001
	gMG group	HC group	20.4486*	2.9144	.000
		oMG group	9.3159*	2.4096	.001

: n s T r e g

	(I) group	(J) group	9 5 %	
Tukey HSD	HC group	oMG group	-17.872	-4.393
		gMG group	-26.936	-13.961
	oMG group	HC group	4.393	17.872
		gMG group	-15.898	-2.734
	gMG group	HC group	13.961	26.936
		oMG group	2.734	15.898
Games-Howell	HC group	oMG group	-18.145	-4.120
		gMG group	-27.587	-13.310
	oMG group	HC group	4.120	18.145
		gMG group	-15.199	-3.433
	gMG group	HC group	13.310	27.587
		oMG group	3.433	15.199

* . 0 . 0 5

nsTreg

	group	N	alpha = 0.05 1	2	3
Tukey HSD[a,b]	HC group	19	53.889		
	oMG group	18		65.022	
	gMG group	21			74.338
			1.000	1.000	1.000

a . = 1 9 . 2 5 5
b . I

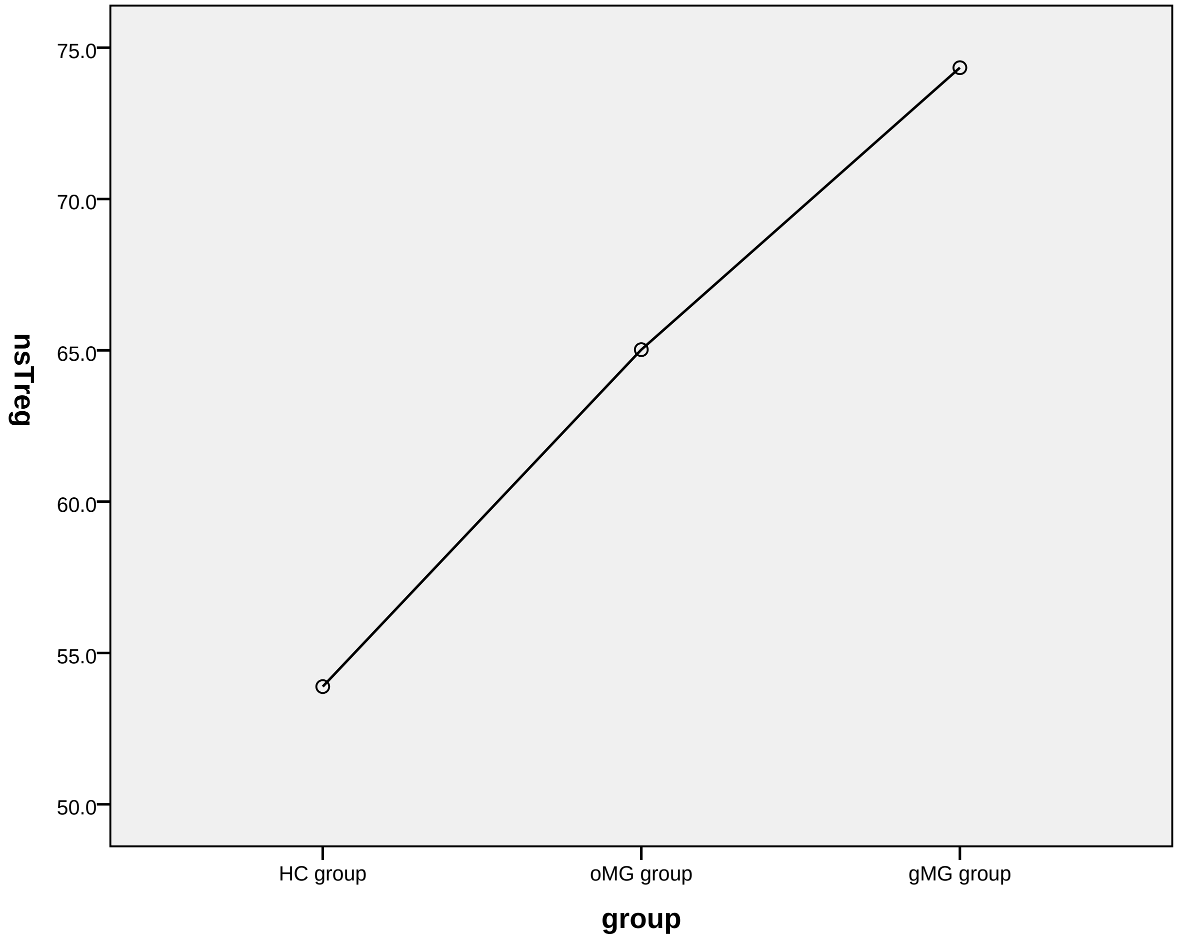


```
EXAMINE VARIABLES=aTreg BY group
  /PLOT BOXPLOT NPPLOT
  /COMPARE GROUPS
  /STATISTICS NONE
  /CINTERVAL 95
  /MISSING LISTWISE
  /NOTOTAL.
```

	16-1-2022 220216
	1
	<none>
	<none>
	<none>
N	58
	EXAMINE VARIABLES=aTreg BY group /PLOT BOXPLOT NPPLOT /COMPARE GROUPS /STATISTICS NONE /CINTERVAL 95 /MISSING LISTWISE /NOTOTAL.
	00 00:00:00.703
	00 00:00:00.695

[1]

group

	group	N		N		N	
aTreg	HC group	19	100.0%	0	.0%	19	100.0%
	oMG group	18	100.0%	0	.0%	18	100.0%
	gMG group	21	100.0%	0	.0%	21	100.0%

		Kolmogorov-Smirnov[a]			Shapiro-Wilk		
	group		df	Sig.		df	Sig.
aTreg	HC group	.111	19	.200*	.956	19	.503
	oMG group	.146	18	.200*	.919	18	.126
	gMG group	.172	21	.105	.930	21	.139

a. Lilliefors
*.

aTreg

Q - Q

aTreg Q-Q

group= HC group

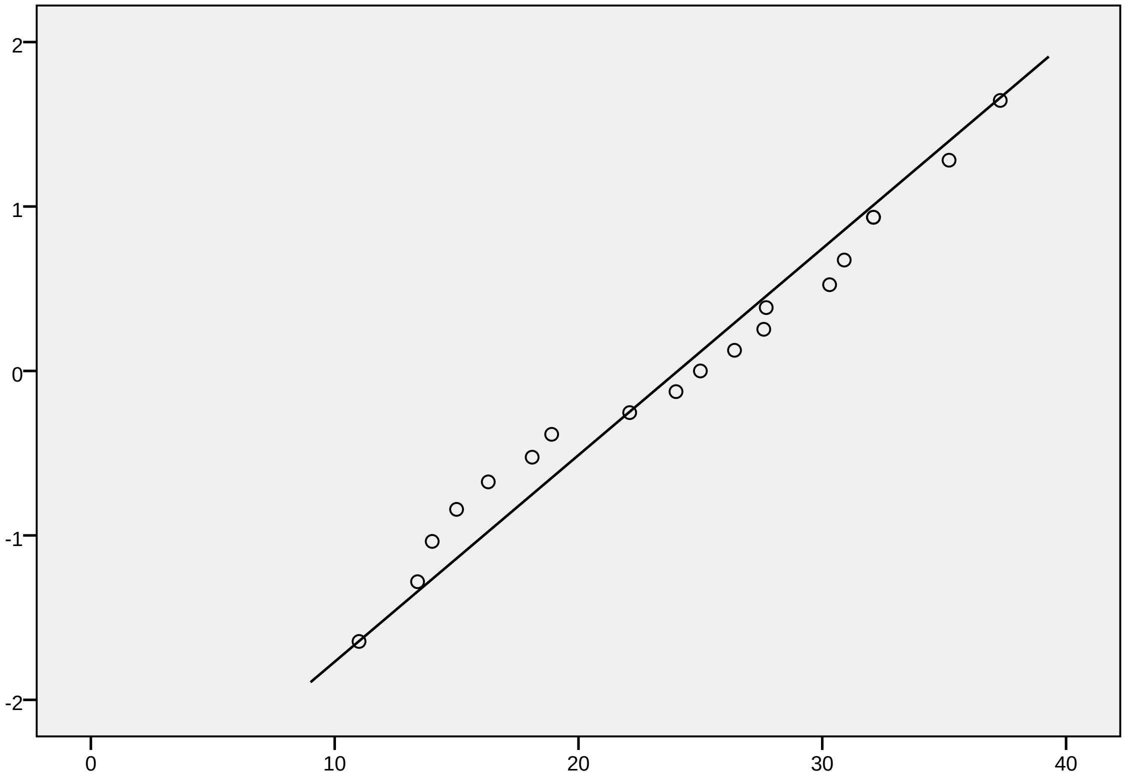

aTreg Q-Q

group= oMG group

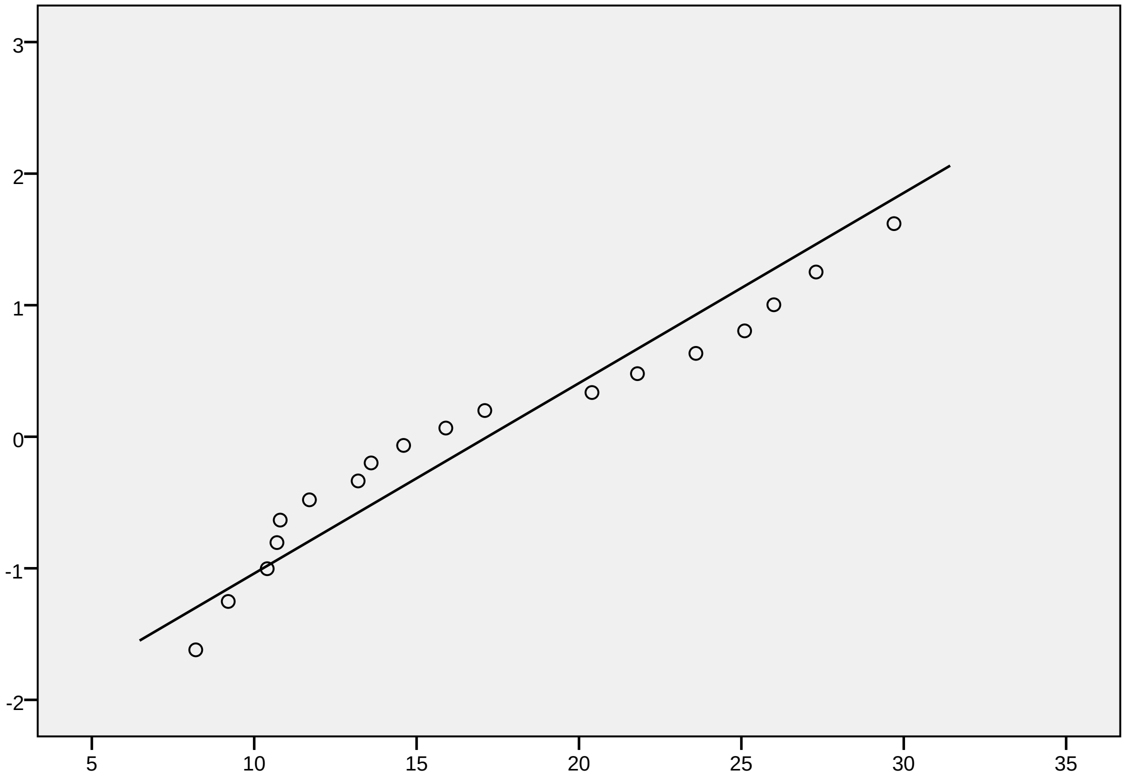

aTreg Q-Q

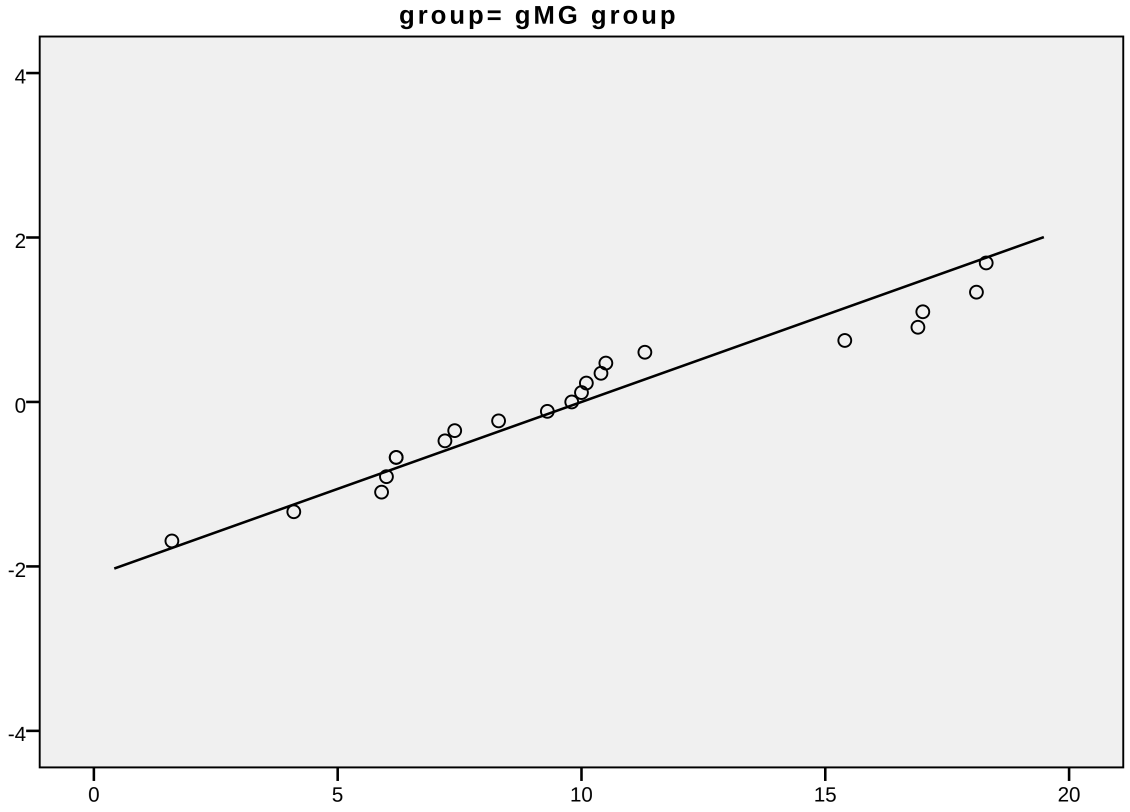

Q - Q

aTreg Q-Q

group= HC group

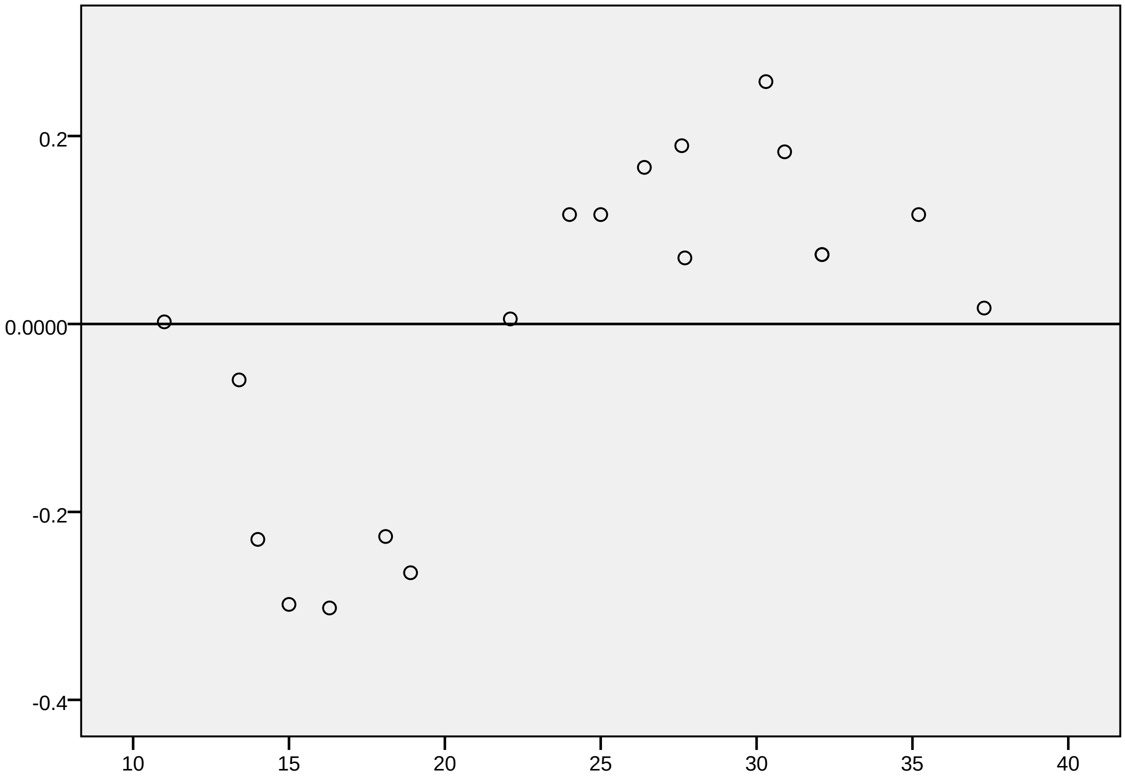

aTreg Q-Q

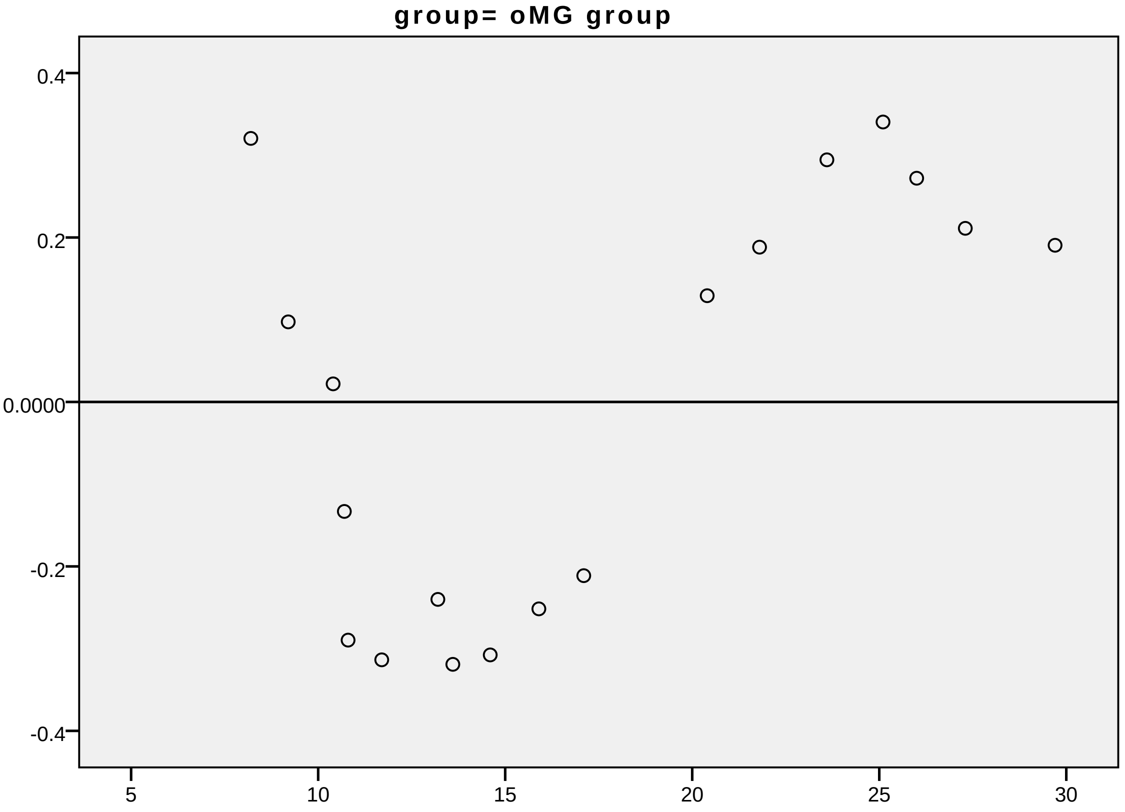

a T r e g   Q - Q

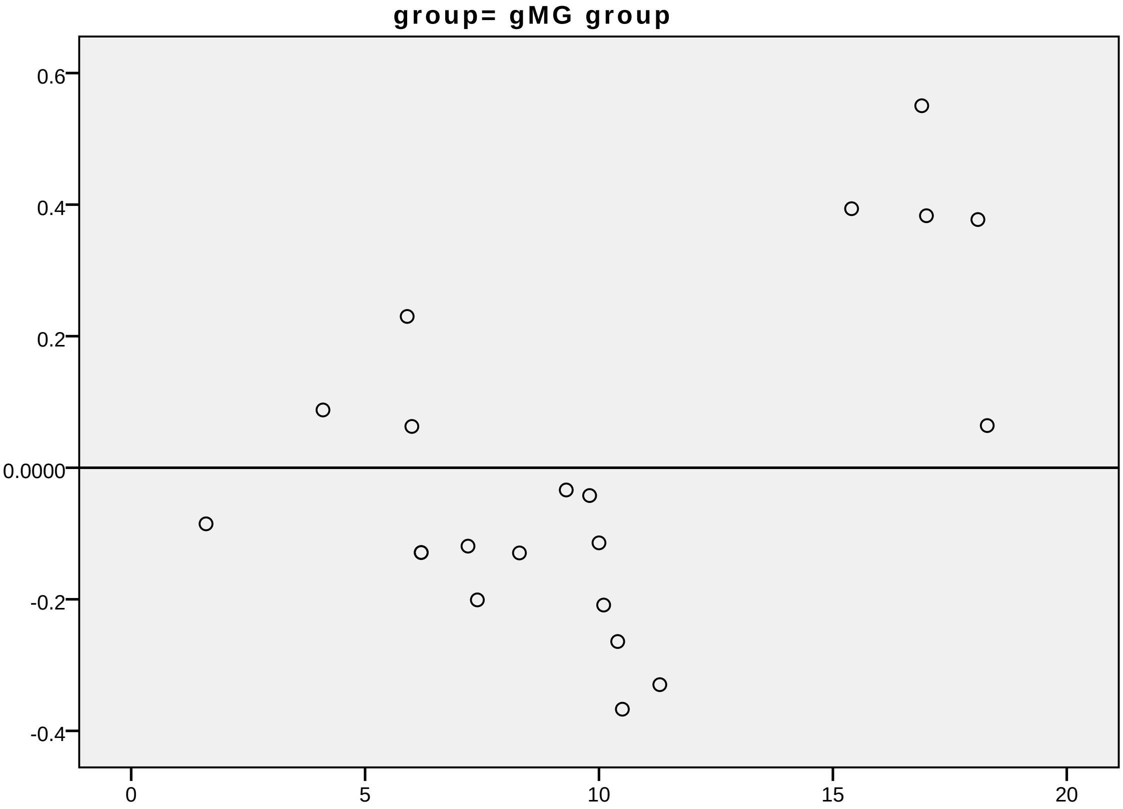

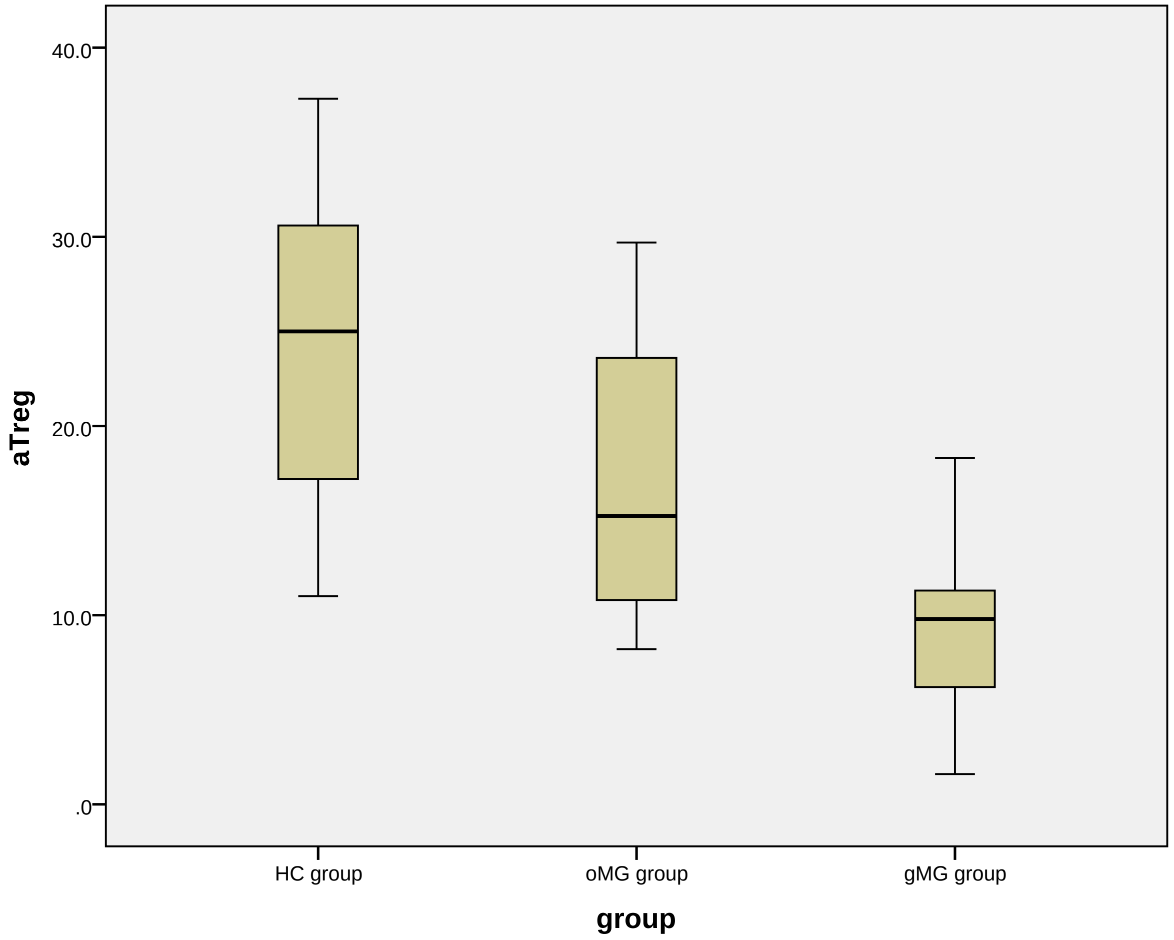


```
ONEWAY aTreg BY group
  /STATISTICS DESCRIPTIVES HOMOGENEITY WELCH
  /PLOT MEANS
  /MISSING ANALYSIS
  /POSTHOC=TUKEY GH ALPHA(0.05).
```

	16-1-2022 220226
	1
	<none>
	<none>
	<none>
N	58
	ONEWAY aTreg BY group /STATISTICS DESCRIPTIVES HOMOGENEITY WELCH /PLOT MEANS /MISSING ANALYSIS /POSTHOC=TUKEY GH ALPHA (0.05).
	00 00:00:00.141
	00 00:00:00.123

[1]

aTreg

	N				95 %	
HC group	19	24.074	7.9591	1.8259	20.238	27.910
oMG group	18	17.183	6.9137	1.6296	13.745	20.621
gMG group	21	10.000	4.7300	1.0322	7.847	12.153
	58	16.840	8.7651	1.1509	14.535	19.144

aTreg

HC group	11.0	37.3
oMG group	8.2	29.7
gMG group	1.6	18.3
	1.6	37.3

aTreg

Levene	df1	df2	
4.615	2	55	.014

ANOVA

aTreg

		df		F	
	1978.817	2	989.408	22.671	.000
	2400.282	55	43.641		
	4379.099	57			

aTreg

	[a]	df1	df2	
Welch	24.086	2	33.250	.000

a . F

: a T r e g

	(I) group	(J) group	(I - J)		
Tukey HSD	HC group	oMG group	6.8904*	2.1729	.007
		gMG group	14.0737*	2.0917	.000
	oMG group	HC group	-6.8904*	2.1729	.007
		gMG group	7.1833*	2.1220	.004
	gMG group	HC group	-14.0737*	2.0917	.000
		oMG group	-7.1833*	2.1220	.004
Games-Howell	HC group	oMG group	6.8904*	2.4474	.021
		gMG group	14.0737*	2.0975	.000
	oMG group	HC group	-6.8904*	2.4474	.021
		gMG group	7.1833*	1.9290	.002
	gMG group	HC group	-14.0737*	2.0975	.000
		oMG group	-7.1833*	1.9290	.002

: a T r e g

	(I) group	(J) group	9 5 %	
Tukey HSD	HC group	oMG group	1.656	12.124
		gMG group	9.035	19.112
	oMG group	HC group	-12.124	-1.656
		gMG group	2.072	12.295
	gMG group	HC group	-19.112	-9.035
		oMG group	-12.295	-2.072
Games-Howell	HC group	oMG group	.899	12.882
		gMG group	8.891	19.257
	oMG group	HC group	-12.882	-.899
		gMG group	2.423	11.944
	gMG group	HC group	-19.257	-8.891
		oMG group	-11.944	-2.423

* . 0 . 0 5

aTreg

	group	N	a l p h a = 0 . 0 5		
			1	2	3
Tukey HSD[a,b]	gMG group	21	10.000		
	oMG group	18		17.183	
	HC group	19			24.074
			1.000	1.000	1.000

a . = 1 9 . 2 5 5
b . I

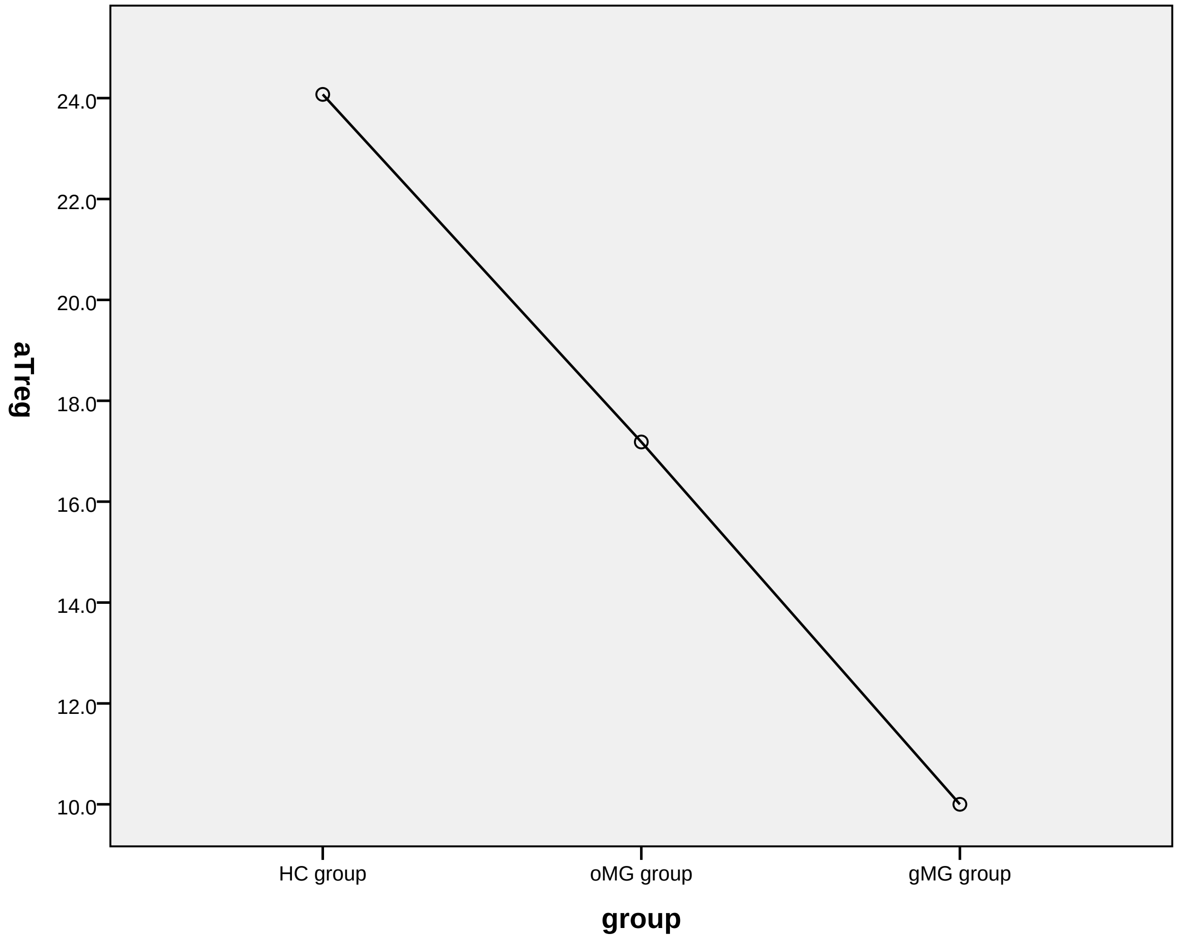


```
CROSSTABS
  /TABLES=sex BY group
  /FORMAT=AVALUE TABLES
  /STATISTICS=CHISQ
  /CELLS=EXPECTED
  /COUNT ROUND CELL.
```

	16-1-2022 220900
	1
	<none>
	<none>
	<none>
N	58
	CROSSTABS<br>/TABLES=sex BY group<br>/FORMAT=AVALUE TABLES<br>/STATISTICS=CHISQ<br>/CELLS=EXPECTED<br>/COUNT ROUND CELL.
	00 00:00:00.000
	00 00:00:00.001
	2
	174762

[1]

	N		N		N	
sex * group	58	100.0%	0	.0%	58	100.0%

sex* group

		group			
		HC group	oMG group	gMG group	
sex	0	8.8	8.4	9.8	27.0
	1	10.2	9.6	11.2	31.0
		19.0	18.0	21.0	58.0

		df	Sig. ( )
Pearson	.047[a]	2	.977
	.047	2	.977
	.000	1	.983
N	58		

a. 0 (.0%) 5
8.38

```
CROSSTABS
  /TABLES=sex BY group
  /FORMAT=AVALUE TABLES
  /STATISTICS=CHISQ
  /CELLS=COUNT COLUMN BPROP
  /COUNT ROUND CELL.
```

	16-1-2022 220948
	1
	<none>
	<none>
	<none>
N	58
	CROSSTABS<br>/TABLES=sex BY group<br>/FORMAT=AVALUE TABLES<br>/STATISTICS=CHISQ<br>/CELLS=COUNT COLUMN BPROP<br>/COUNT ROUND CELL.
	00 00:00:00.000
	00 00:00:00.001
	2
	174762

[1]

	N		N		N	
sex * group	58	100.0%	0	.0%	58	100.0%

sex* group

			group			
			HC group	oMG group	gMG group	
sex	0		9a	8a	10a	27
		group %	47.4%	44.4%	47.6%	46.6%
	1		10a	10a	11a	31
		group %	52.6%	55.6%	52.4%	53.4%
			19	18	21	58
		group %	100.0%	100.0%	100.0%	100.0%

group .05

		df	Sig. ( )
Pearson	.047[a]	2	.977
	.047	2	.977
	.000	1	.983
N	58		

a. 0 (.0%) 5
8.38
